# Valid gene expression normalization by RT-qPCR in studies on hPDL fibroblasts with focus on orthodontic tooth movement and periodontitis

**DOI:** 10.1038/s41598-017-15281-0

**Published:** 2017-11-07

**Authors:** Christian Kirschneck, Sarah Batschkus, Peter Proff, Josef Köstler, Gerrit Spanier, Agnes Schröder

**Affiliations:** 1 Department of Orthodontics, University Medical Centre of Regensburg, Regensburg, D-93053 Germany; 2 Department of Orthodontics, University of Goettingen, Goettingen, D-37075 Germany; 3 Institute of Microbiology and Hygiene, University Medical Centre of Regensburg, Regensburg, D-93053 Germany; 4 Department of Cranial and Maxillo-Facial Surgery, University Medical Centre of Regensburg, Regensburg, D-93053 Germany

## Abstract

Meaningful, reliable and valid mRNA expression analyses by real-time quantitative PCR (RT-qPCR) can only be achieved, if suitable reference genes are chosen for normalization and if appropriate RT-qPCR quality standards are met. Human periodontal ligament (hPDL) fibroblasts play a major mediating role in orthodontic tooth movement and periodontitis. Despite corresponding *in-vitro* gene expression studies being a focus of interest for many years, no information is available for hPDL fibroblasts on suitable reference genes, which are generally used in RT-qPCR experiments to normalize variability between samples. The aim of this study was to identify and validate suitable reference genes for normalization in untreated hPDL fibroblasts as well as experiments on orthodontic tooth movement or periodontitis (Aggregatibacter actinomycetemcomitans). We investigated the suitability of 13 candidate reference genes using four different algorithms (geNorm, NormFinder, comparative ΔC_q_ and BestKeeper) and ranked them according to their expression stability. Overall PPIB (peptidylprolyl isomerase A), TBP (TATA-box-binding protein) and RPL22 (ribosomal protein 22) were found to be most stably expressed with two genes in conjunction sufficient for reliable normalization. This study provides an accurate tool for quantitative gene expression analysis in hPDL fibroblasts according to the MIQE guidelines and shows that reference gene reliability is treatment-specific.

## Introduction

Orthodontics and periodontology are specialties of dentistry tending to the treatment of misaligned teeth/jaws and bacterially induced inflammation of the periodontal tissues (periodontitis), respectively, with several interactive associations existing^1^. In orthodontics mechanical forces applied to the teeth result in tensile and pressure zones within the periodontal ligament (PDL)^2^. PDL fibroblasts react to this mechanical strain with an increased synthesis of proinflammatory enzymes, cytokines and chemokines^2–4^, triggering osteoclastogenesis. Bacterial toxins from periodontal pathogens in periodontitis, such as the gram-negative *Aggregatibacter actinomycetemcomitans (Agac)*, the key pathogen in aggressive periodontitis^5^, can in a similar way stimulate PDL fibroblasts, which are thus essential both for mediating orthodontic tooth movement and bacterial periodontitis.

Real-time quantitative PCR (RT-qPCR) and DNA microarray analysis are the methods of choice to analyse transcription of cellular genes^6,7^. In contrast to microarray analysis, which allows expression profiling of a high number of genes, RT-qPCR enables a precise quantification of gene expression differences in physiological, pathological and various experimental states^8–10^. However, a reliable RT-qPCR setup is necessary to achieve valid results. To improve quality and reproducibility of RT-qPCR experiments, Bustin *et al*. published the MIQE guidelines^11^ in 2009, detailing the minimum information for publication of quantitative real-time PCR experiments. A view in current literature shows that many gene expression studies did not perform, consider or report important aspects such as RNA integrity, qPCR-efficiency, primer specifity or secondary structure analyses of primers and amplicons, thus limiting their scientific validity and reliability^6,7,12^. This is particularly the case in the field of dentistry, with RT-qPCR studies on cells of teeth and the surrounding periodontal tissue continuously increasing, particularly in orthodontics^12–18^ and periodontology^19–24^.

In RT-qPCR absolute quantification of gene expression is prone to errors due to intra- or interkinetic variations as well as variations in yield and efficiency during RNA isolation, reverse transcription and qPCR^8,25^. Therefore relative gene expression is usually calculated by normalization of a target gene expression to one or more reference genes^7^, which mostly regulate basic cellular functions and are deemed to be stably expressed in different experimental conditions as well as cell and tissue types^7,26,27^. But “perfect” reference genes do not exist^6,7,28,29^. Various studies have shown that the stability of reference genes can vary considerably between cell types, different tissues and even experimental conditions in the same specimen^6,7,28^. Thus an individual validation of suitable reference genes is required to allow a valid interpretation of relative gene expression data^8,25^. Otherwise relative gene expression of target genes may be over- or underestimated or even contrary to the expression actually occurring^30^. For various human cell types, tissues and experimental conditions valid reference genes have been identified^27,31–34^. However, no valid reference genes for gene expression studies on hPDL fibroblasts have been published so far despite many studies investigating periodontitis and orthodontic tooth movement using this cell type^3,12–23,35^. In most cases reference genes were not validated and normalization was performed using only one gene.

In the present study we wanted to introduce the MIQE guidelines^11^ to *in-vitro* experiments in the field of dentistry and to identify the ideal number and type of reference genes for qPCR gene expression studies on hPDL fibroblasts, particularly in experiments on orthodontic tooth movement and periodontitis, by determining the relative expression stability of 13 commonly used reference genes using four mathematical algorithms (geNorm^29^, NormFinder^36^, BestKeeper^37^, comparative ΔC_q_^31^). In addition, we investigated the conformity and thus reliability of these algorithms for bioinformatical analyses of reference gene stability.

## Results

### *In silico* analysis of primer and amplicon quality and suitability

We selected 13 candidate reference genes based on their frequent usage for normalization in gene expression studies with differing functions in cell metabolism to minimize co-regulation (Table 1)^7^. All primers were newly designed by the authors with NCBI PrimerBLAST^38^ according to the MIQE guidelines^11,39–41^ (Supplementary Table 1) to minimize risk of bias. Intron-flanking primer pairs to prevent a co-amplification of genomic DNA could be designed for all candidate reference genes except RNA18S5 as well as sufficient absence of hairpin structures and self-/cross-dimer formation confirmed at annealing temperature (∆G ≥ −3,5 kcal/mol^41^, BeaconDesigner™ Free Edition, Premier BioSoft International, Palo Alto, CA, USA) (Supplementary Data 1). Target amplicon sequences were chosen to range from 60 to 150 bp with a GC content of 35–65% (Table 1) and no secondary structures were found at annealing temperature^41^ (60 °C, Supplementary Data 1), determined by UNAFold (Integrated DNA Technologies Inc., Coralville, IA, USA). *In silico* specifity of constructed primers was corroborated by PrimerBLAST^38^ and cross-checked using the UCSC Genome Browser (University of California, CA, USA) (Supplementary Data 1). Using NCBI PrimerBLAST^38^ and PrimerCheck (SpliceCenter of Genomics and Bioinformatics Group, LMP, CCR, NCI), we could confirm that primers also targeted possible splicing and transcript variants (except for RNA18S5), whereas no pseudogenes, retropseudogenes or other homologs were found to be amplified (Table 1, Supplementary Data 1), except for RNA18S5 (co-amplification of RNA45S5). For four of the 13 to be investigated candidate reference genes (UBC, GUSB, ACTB, TUBB), it was not possible to design a specific primer pair meeting all specified quality criteria^11,39–41^, which is why they were exempted from further qPCR analysis (Table 1). A commercially available, non-intron-flanking primer pair for TUBB (PPH17836A-200, Qiagen, Hilden, Germany), however, was tested alongside the custom primers to comparatively assess primer specifity and relative gene expression stability.

**Table 1:** RT-qPCR gene, primer and target/amplicon information for the 13 investigated candidate references genes.

**Gene symbol**	**Gene name** (Homo sapiens)	**Gene function**	**Accession Number** (NCBI GeneBank)	**Chrom-osoma location** (length)	**5´-forward primer-3´** (length/T_m_/%GC/max. ∆G Hairpin &Self-Dimer/Self-Comp./Self-3’-Comp.)	**5´-reverse primer-3´** (length/T_m_/%GC/max. ∆G Hairpin &Self-Dimer/Self-Comp./Self-3’-Comp.)	**Primer Location** (max. ∆G Cross-Dimer)	**Amplicon** (length, %GC, T_m_, SSAT)	**Amplicon location** (bp of Start/Stop)	**Intron-flanking** (length)	**In silico qPCR specifity**	**Variants targeted (Tran-script /Splice)**
**GAPDH**	glyceraldehyde-3-phosphate dehydrogenase	enzyme in glycolysis and gluconeo-genesis	NM_002046.5	12p13.31 (1421 bp)	TGCCCTCAACGACCACTTTG (20 bp/59.4 °C/55.0%/−0.7/3/2)	CCACCACCCTGTTGCTGTAG (20 bp/61.4 °C/60.0%/0.0/4/2)	exon 8/9 (−2.4)	74 bp, 50.0%, 84.0 °C, no SSAT	1091/1164	Yes (104 bp)	Yes (BLAST /UCSC)	Yes
**PPIB**	peptidylprolyl isomerase A (cyclophilin B)	ER cyclosporine-binding protein	NM_000942.4	15q21-q22 (1045 bp)	TTCCATCGTGTAATCAAGGACTTC (24 bp/59.3 °C/41.7%/−1.3/4/2)	GCTCACCGTAGATGCTCTTTC (21 bp/59.8 °C/52.4%/−0.7/4/0)	exon ¾ (−2.1)	88 bp, 53.4%, 86.1 °C, no SSAT	446/533	Yes (3194 bp)	Yes (BLAST /UCSC)	Yes
**YWHAZ**	tyrosine 3-monoo-xygenase /tryptophan 5-monoo-xygenase activation protein, zeta	signal transduction, apoptotic pathways	NM_003406.3	8q23.1 (3003 bp)	AGGAGATTACTACCGTTACTTGGC(24 bp/61.0 °C/46%/0.0/4/2)	AGCTTCTTGGTATGCTTGTTGTG (23 bp/58.9 °C/43%/−3.0/4/0)	exon 8/9 (−2.2)	91 bp, 47.3%, 84.0 °C, no SSAT	504/572	Yes (617 bp)	Yes (BLAST /UCSC)	Yes
**POLR2A**	polymerase (RNA) II (DNA directed) polypeptide A, 220 kDa	transcription of DNA into mRNA	NM_000937.4	17p13.1 (6738 bp)	TCGCTTACTGTCTTCCTGTTGG (22 bp/60.3 °C/50.0%/0.0/3/0)	TGTGTTGGCAGTCACCTTCC (20 bp/59.4 °C/55.0%/−1.3/3./ 3)	exon 21/22 (−2.5)	108 bp, 53.7%, 87.8 °C, no SSAT	3798/3905	Yes (468 bp)	Yes (BLAST /UCSC)	Yes
**TBP**	TATA-box-binding protein	general transcription factor	NM_003194.4	6q27 (1921 bp)	CGGCTGTTTAACTTCGCTTCC (21bp/59.8 °C/52.4%/−0.8/5/0)	TGGGTTATCTTCACACGCCAAG (22 bp/60.3 °C/50.0%/−1.5/3/2)	exon ½ (−2.4)	86 bp, 51.2%, 85.6 °C, no SSAT	79/164	Yes (2418 bp)	Yes (BLAST /UCSC)	Yes
**RPL22**	ribosomal protein L22	translation of mRNA in protein	NM_000983.3	1p36.31 (2099 bp)	TGATTGCACCCACCCTGTAG (20 bp/59.4 °C/55.0%/−3.4/4/2)	GGTTCCCAGCTTTTCCGTTC (20 bp/59.4 °C/55.0%/−3.0/4/0)	exon 2/3 (−1.5)	98 bp, 44.9%, 83.8 °C, no SSAT	115/212	Yes (4597 bp)	Yes (BLAST /UCSC)	Yes
**EEF1A1**	eukaryotic translation elongation factor 1 alpha 1	enzymatic delivery of aminoacyl tRNAs to ribosome	NM_001402.5	6q14.1 (3528 bp)	CCTGCCTCTCCAGGATGTCTAC (22 bp/64.0 °C/59.1%/−3.0/5/2)	GGAGCAAAGGTGACCACCATAC (22 bp/62.1 °C/54.6%/−3.2/6/2)	exon 5/6 (−2.9)	105 bp, 52.4%, 86.5 °C, no SSAT	804/908	Yes (87 bp)	Yes (BLAST /UCSC)	Yes
**RPLP0**	ribosomal protein, large, P0	translation of mRNA in protein	NM_001002.3	12q24.2 (1229 bp)	GAAACTCTGCATTCTCGCTTCC(22 bp/60.3 °C/50.0%/−3.4/4/0)	GACTCGTTTGTACCCGTTGATG (22 bp/60.3 °C/50.0%/−2.0/4/0)	exon 6/7 (−1.8)	120 bp, 50.8%, 86.5 °C, no SSAT	803/921	Yes (1091 bp)	Yes (BLAST /UCSC)	Yes
**RNA18S5**	18S ribosomal 5	ribosomal RNA, translation of mRNA in protein	NR_003286.2	22p12 (1869bp)	AACTGCGAATGGCTCATTAAATCw (23 bp/57.1 °C/39.1%/−1.7/6/3)	GCCCGTCGGCATGTATTAG(19 bp/58.8 °C/57.9%/−2.4/5/1)	(−2.4)	103 bp, 46.6%, 83.7 °C, no SSAT	84/186	No (rRNA)	No(RNA45S5 also targeted)	—
**UBC**	ubiquitin C	maintains ubiquitin levels under stress (protein removal)	NM_021009.6	12q24.3 (2594 bp)	No specific primer pair meeting all criteria could be designed.	—	—	—	—	—	—	
**GUSB**	glucuro-nidase, beta	breakdown of glycosamin-oglycans in lysosomes	NM_000181.3	7q21.11 (2321 bp)	No specific primer pair meeting all criteria could be designed.	—	—	—	—	—	—	
**ACTB**	actin, beta	cytoskeletal structural protein	NM_001101.3	7p22 (1852bp)	No specific primer pair meeting all criteria could be designed.	—	—	—	—	—	—	
**TUBB**	tubulin, beta class I	cytoskeletal structural protein	NM_001293212.1	6p21.33 (2772 bp)	No specific primer pair meeting all criteria could be designed.	—	—	—	—	—	—	

ER = endoplasmic reticulum; T_m_ = melting temperature of primer/specific qPCR product (amplicon); %GC = guanine/cytosine content; bp = base pairs; Comp. = Complementarity; SSAT = secondary structure at annealing temperature.

### Primer specifity, RT-qPCR efficiencies and C_q_ expression levels

**Figure 1:**
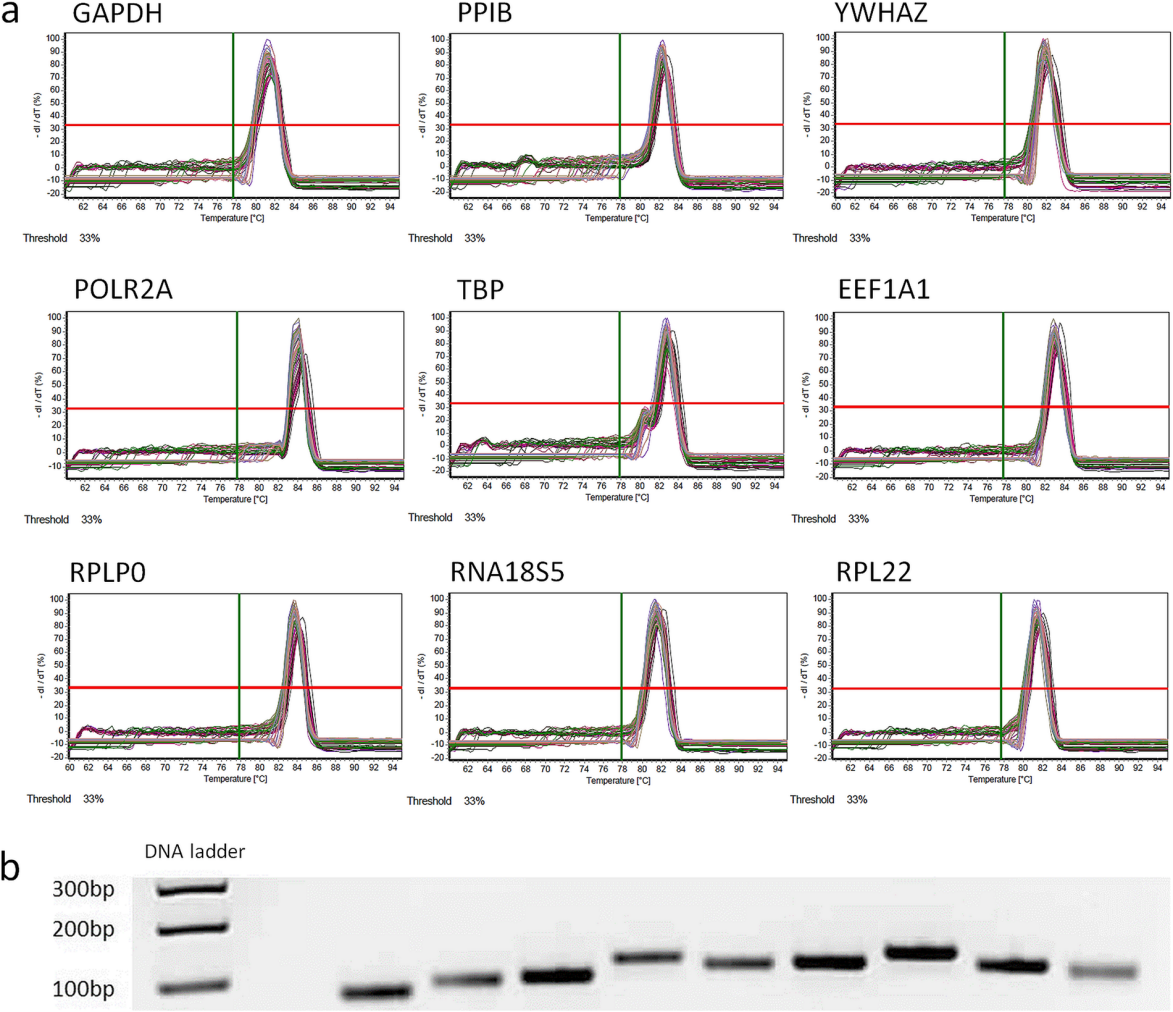
Specifity of RT-qPCR amplification as determined by (**a**) melting curve analysis and (**b**) agarose gel electrophoresis of RT-qPCR products. For each candidate reference gene/primer pair we found a single fluorescent band at the expected amplicon size. bp = base pairs. Gene names see Table 1. All RT-qPCR products were run concurrently and adjacently on the same gel, which was recorded with the gel documentation system Genoplex 2 (VWR International GmbH, Darmstadt, Germany) and its software GenoCapture (version 7.01, Synoptics Ltd., Cambridge, UK - automatic exposure, exposure time 80 ms, no binning, transillumination) as secure gel data (*.sgd) and exported as TIF image, which was inverted and cropped to encompass the relevant gel area. The uncropped original gel is provided as Supplementary Figure 2.

**Figure 2:**
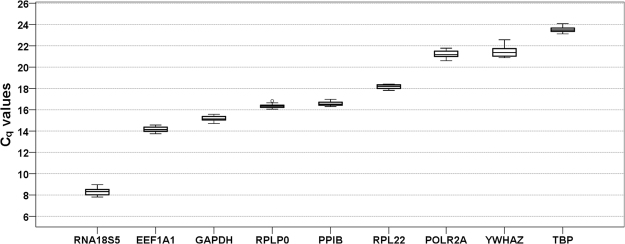
Expression levels of candidate reference genes across all experimental groups (n = 18). Values are presented as quantification cycle (C_q_, mean of triplicate technical replicates) as second derivative maximum of the fluorescence curve and are inversely proportional to the initial amount of cDNA. Genes are ordered from left (highest expression) to right (lowest expression) according to their mean C_q_ values. Gene names see Table 1. Boxplots show median, interquartile range (box) and data range (whiskers).

Primer specifity was confirmed by melting curve analysis (Fig. 1a, Supplementary Data 3) and agarose gel electrophoresis, which showed a single band at the expected molecular amplicon weight per primer pair (Fig. 1b). Primer (factor-specific) and amplification (sample-specific) efficiencies ranged from 91.7% to 100.3% (E_P_ , Table 2, Supplementary Data 4) and 87.3% to 113.4% (E_A_, Table 2) with a minimum coefficient of determination in the linear dynamic range (LDR) of 0.9949. The highest SD of the arithmetic mean of C_q_ of the three technical replicates among all samples (n = 18) for each candidate gene was 0.53 C_q_ (EEF1A1) with a mean SD of 0.08–0.28 for the individual candidate reference genes (technical reliability, Table 2). In addition biological variation of C_q_ values was limited within experimental groups for each gene with SD ranging from 0.05 to 0.22 (Supplementary Table 3), except for YWHAZ and RNA18S5 (SD 0.31–0.62). C_q_ values of the investigated reference genes, which inversely correspond to the initial amount of cDNA template, ranged from 8.30 to 23.52 cycles (Fig. 2, Supplementary Table 3) with lowest values observed for RNA18S5 and highest for POLR2A, YWHAZ and TBP. Specifity, efficiencies and C_q_ expression levels for the commercially available primer pair for TUBB are given in Supplementary Data 5.

**Table 2:** Primer efficiency (factor-specific) and coefficients of determination derived from a standard curve for each primer pair (6x log_10_ dilution of cDNA stock solution, random untreated sample) as well as technical repeatability (intraassay reliability, n = 18) and amplification efficiency (sample-specific), calculated using LinRegPCR software (http://LinRegPCR.HFRC.nl; n = 18 in triplets).

**Gene symbol**	**Slope**	**Primer efficiency E**_**P**_ **[%] (2** ^**E**^ **P** ^**/100%**^ **)**	**Coefficient of determination R** ^**2**^	**Intraassay reliability** SD of mean of C_q_* (mean, min./max.)	**Amplification Efficiency E**_**A**_ **[%] (2** ^**E**^ **A** ^**/100%**^ **)**
GAPDH	−3.480	93.8 (1.916)	0.9998	0.12 0.03/0.24	91.1 (1.880)
PPIB	−3.509	92.7 (1.902)	0.9996	0.13 0.01/0.29	90.6 (1.874)
YWHAZ	−3.488	93.5 (1.912)	0.9993	0.11 0.03/0.32	91.3 (1.883)
POLR2A	−3.520	92.3 (1.897)	0.9984	0.17 0.04/0.35	87.3 (1.832)
TBP	−3.538	91.7 (1.888)	0.9974	0.11 0.02/0.27	89.7 (1.862)
RPL22	−3.403	96.7 (1.955)	0.9949	0.13 0.02/0.33	90.1 (1.868)
EEF1A1	−3.315	100.3 (2.004)	0.9951	**0.28 0.19**/**0.53**	89.5 (1.860)
RPLP0	−3.509	92.7 (1.902)	0.9992	0.17 0.05/0.36	87.8 (1.838)
RNA18S5	−3.319	100.1 (2.002)	0.9974	0.08 0.03/0.20	113.4 (2.195)

^*^Of three technical replicates (triplet) among all biological replicates (n = 18). CI = confidence interval.

### Optimal number of reference genes for normalization

**Figure 3:**
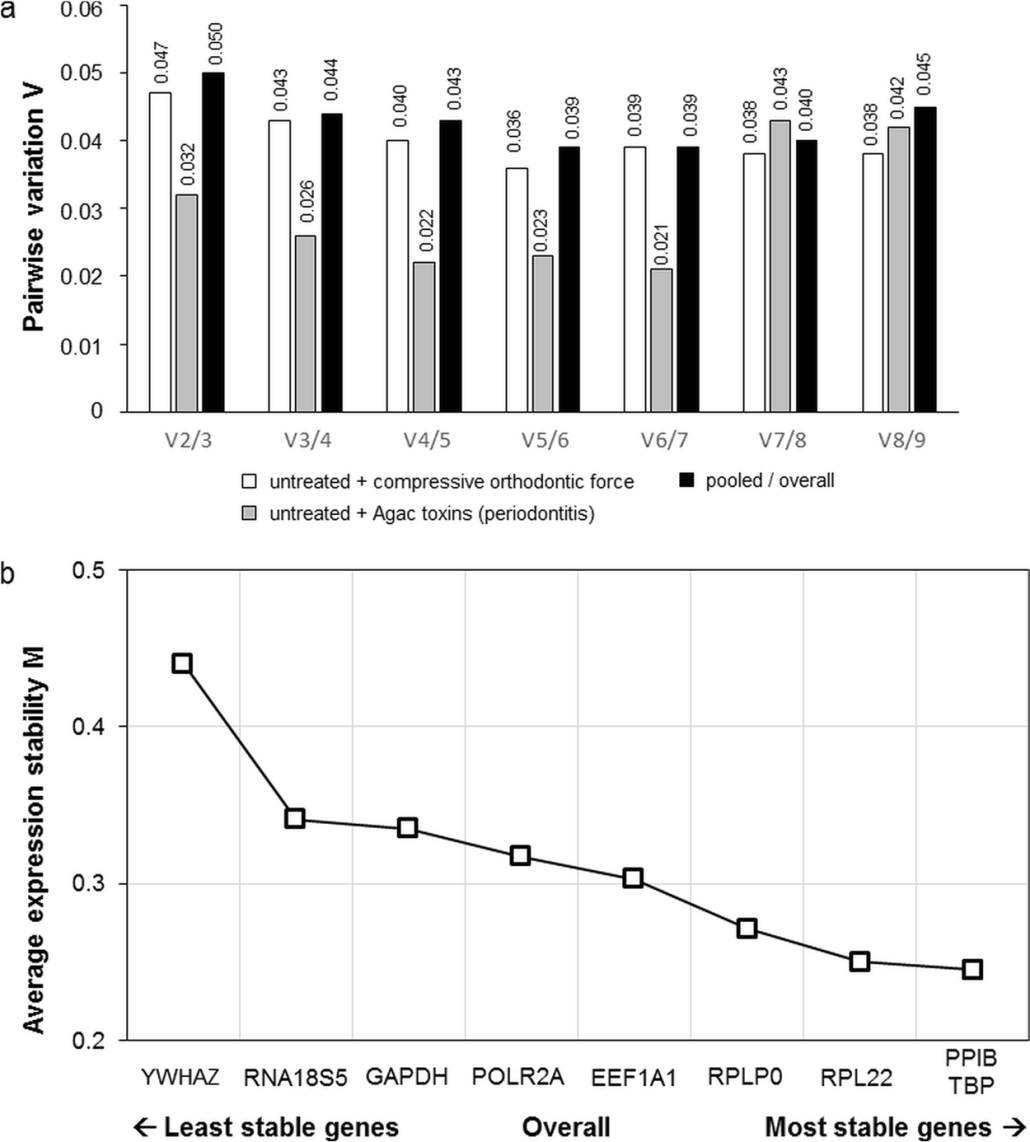
GeNorm expression stability analysis of the nine candidate reference genes, for which specific primers could be constructed. (**a**) Optimal number of reference genes for hPDL RT-qPCR data normalization in orthodontic studies (compressive orthodontic force vs. untreated control, n = 12), studies on periodontitis (Agac toxins vs. untreated control, n = 12) and pooled/overall (n = 18). (**b**) Average expression stability values of overall (pooled) specimens derived by stepwise exclusion of the least stable reference gene across all specimens and experimental conditions (n = 18). A smaller M value indicates a more stable gene expression. Gene names see Table 1.

geNorm analysis revealed that the use of two reference genes for normalization in RT-qPCR was adequate for studies in hPDL fibroblasts in all experimental conditions (Fig. 3a). Average pairwise variation V_n_/V_n+1_ after inclusion of a third reference gene was below 0.15 for all tested conditions (Fig. 3a).

### Relative stability of candidate reference genes

With geNorm the most stably expressed reference genes for the pooled/overall conditions were found to be PPIB and TBP (Table 3, Fig. 3b). When we analysed the conditions for experimental orthodontic tooth movement and for periodontitis separately with geNorm, PPIB and RPL22 were most stable in orthodontic setups, whereas TBP and PPIB were most stably expressed in experiments on periodontitis (Agac toxins, Table 3). NormFinder confirmed geNorm findings and also identified RPL22 and PPIB as most stable genes in combined control and compressive force conditions and PPIB and TBP for combined control and Agac toxin treatment as well as overall combined experimental conditions (Table 3). The comparative ΔC_q_ method^31^ was also in line with geNorm and NormFinder (Table 3). In contrast, the BestKeeper algorithm^37^ suggested RNA18S5 and YWHAZ for compressive force experiments, POLR2A and TBP for Agac toxins and RNA18S5 and TBP for combined experiments as the most stable reference genes (Table 3). Mean SD of mean C_q_ was ≤1 for each gene, as required for stable reference genes. For the three experimental groups (control, compressive orthodontic force, Agac toxins) separate stability rankings were also calculated and are given in Supplementary Table 4. When also considering RT-qPCR data obtained from the commercially available primer pair on TUBB, no influence on the top-ranking, most stable genes was detected (Supplementary Data 5).

**Table 3:** Reference gene stability ranking for hPDL experiments on orthodontic tooth movement (compressive orthodontic force vs. untreated control), experiments on periodontitis (Agac, toxins/bacterial lysate vs. untreated control) and pooled/overall experimental conditions as calculated by the algorithms geNorm, NormFinder, comparative ΔCq and BestKeeper.

**Rank**	**Total (of 4 methods)**		**geNorm**		**NormFinder**			**comparative deltaC**_**q**_		**BestKeeper**			
	Ranking order	Rank sum	Ranking order	Stability value (M)	Ranking order	Stability value (ρ_ig_/σ_i_)	Standard error	Ranking order	Stability value (mean SD of mean ∆C_q_)	Ranking order	Stability value (r)	SD (+/− C_q_)	CV (% C_q_)
**hPDL untreated + compressive orthodontic force (experiments on orthodontic tooth movement, n = 12)**													
1.)	**RPL22**	**6**	**RPL22**	**0.219**	**RPL22**	**0.035**	**0.027**	**RPL22**	**0.232**	**RNA18S5**	**0.910**	**0.259**	**3.110**
2.)	**PPIB**	**11**	**PPIB**	**0.232**	**PPIB**	**0.072**	**0.024**	**PPIB**	**0.246**	**YWHAZ**	**0.887**	**0.373**	**1.728**
3.)	***TBP***	***13***	***TBP***	***0.245***	***TBP***	***0.085***	***0.026***	***TBP***	***0.260***	***RPL22***	***0.842***	***0.121***	***0.665***
4.)	RNA18S5	19	RPLP0	0.255	RPLP0	0.110	0.029	RPLP0	0.268	***TBP***	***0.808***	***0.202***	***0.860***
5.)	RPLP0	19	EEF1A1	0.269	EEF1A1	0.131	0.033	EEF1A1	0.282	***PPIB***	***0.756***	***0.187***	***1.128***
6.)	EEF1A1	23	RNA18S5	0.305	RNA18S5	0.154	0.037	RNA18S5	0.311	POLR2A	0.660	0.357	1.681
7.)	YWHAZ	29	GAPDH	0.316	GAPDH	0.182	0.042	GAPDH	0.334	RPLP0	0.419	0.098	0.601
8.)	GAPDH	30	POLR2A	0.346	POLR2A	0.207	0.047	POLR2A	0.373	EEF1A1	0.388	0.114	0.814
9.)	POLR2A	30	YWHAZ	0.385	YWHAZ	0.238	0.053	YWHAZ	0.416	GAPDH	−0.139	0.117	0.776
**hPDL untreated + Agac toxins/bacterial lysate (experiments on periodontitis, n = 12)**													
1.)	**PPIB**	**6**	**PPIB**	**0.179**	**PPIB**	**0.037**	**0.022**	**PPIB**	**0.189**	**POLR2A**	**0.840**	**0.176**	**0.836**
2.)	**TBP**	**8**	**TBP**	**0.186**	**TBP**	**0.046**	**0.021**	**TBP**	**0.196**	**TBP**	**0.761**	**0.121**	**0.518**
3.)	POLR2A	14	EEF1A1	0.191	POLR2A	0.085	0.024	EEF1A1	0.196	***PPIB***	***0.717***	***0.099***	***0.602***
4.)	EEF1A1	15	POLR2A	0.212	GAPDH	0.086	0.024	***RPL22***	***0.222***	GAPDH	0.683	0.159	1.044
5.)	GAPDH	22	RPLP0	0.213	EEF1A1	0.086	0.024	RPLP0	0.224	RNA18S5	0.643	0.269	3.261
6.)	***RPL22***	***23***	***RPL22***	***0.213***	***RPL22***	***0.102***	***0.026***	POLR2A	0.225	EEF1A1	0.641	0.167	1.170
7.)	RPLP0	24	GAPDH	0.216	RPLP0	0.109	0.028	GAPDH	0.226	RPLP0	0.562	0.172	1.049
8.)	RNA18S5	29	RNA18S5	0.339	RNA18S5	0.208	0.046	RNA18S5	0.347	***RPL22***	***0.557***	***0.164***	***0.902***
9.)	YWHAZ	36	YWHAZ	0.399	YWHAZ	0.260	0.057	YWHAZ	0.425	YWHAZ	0.266	0.318	1.488
**hPDL pooled/overall (experiments on orthodontic tooth movement and periodontitis n = 18)**													
1.)	**PPIB**	**6**	**PPIB**	**0.241**	**PPIB**	**0.062**	**0.021**	**PPIB**	**0.254**	**RNA18S5**	**0.781**	**0.266**	**3.199**
2.)	**TBP**	**8**	**TBP**	**0.249**	**TBP**	**0.073**	**0.021**	**TBP**	**0.263**	**TBP**	**0.751**	**0.173**	**0.735**
3.)	***RPL22***	***14***	***RPL22***	***0.250***	***RPL22***	***0.085***	***0.022***	***RPL22***	***0.263***	***PPIB***	***0.699***	***0.158***	***0.955***
4.)	RPLP0	19	RPLP0	0.271	RPLP0	0.122	0.026	RPLP0	0.287	POLR2A	0.638	0.280	1.320
5.)	POLR2A	22	EEF1A1	0.303	EEF1A1	0.161	0.031	EEF1A1	0.313	YWHAZ	0.638	0.381	1.777
6.)	EEF1A1	23	POLR2A	0.317	POLR2A	0.168	0.032	POLR2A	0.340	RPL22	0.596	0.141	0.776
7.)	RNA18S5	23	GAPDH	0.335	RNA18S5	0.182	0.034	RNA18S5	0.350	RPLP0	0.429	0.149	0.913
8.)	YWHAZ	31	RNA18S5	0.341	GAPDH	0.187	0.035	GAPDH	0.353	EEF1A1	0.399	0.181	1.277
9.)	GAPDH	32	YWHAZ	0.440	YWHAZ	0.280	0.049	YWHAZ	0.472	GAPDH	0.206	0.189	1.245

A higher rank denotes lower expression stability C_q_ = quantification cycle; SD = standard deviation; CV = coefficient of variation; r = Pearson’s correlation coefficient.

### Conformity of mathematical algorithms for reference gene stability analysis

**Figure 4:**
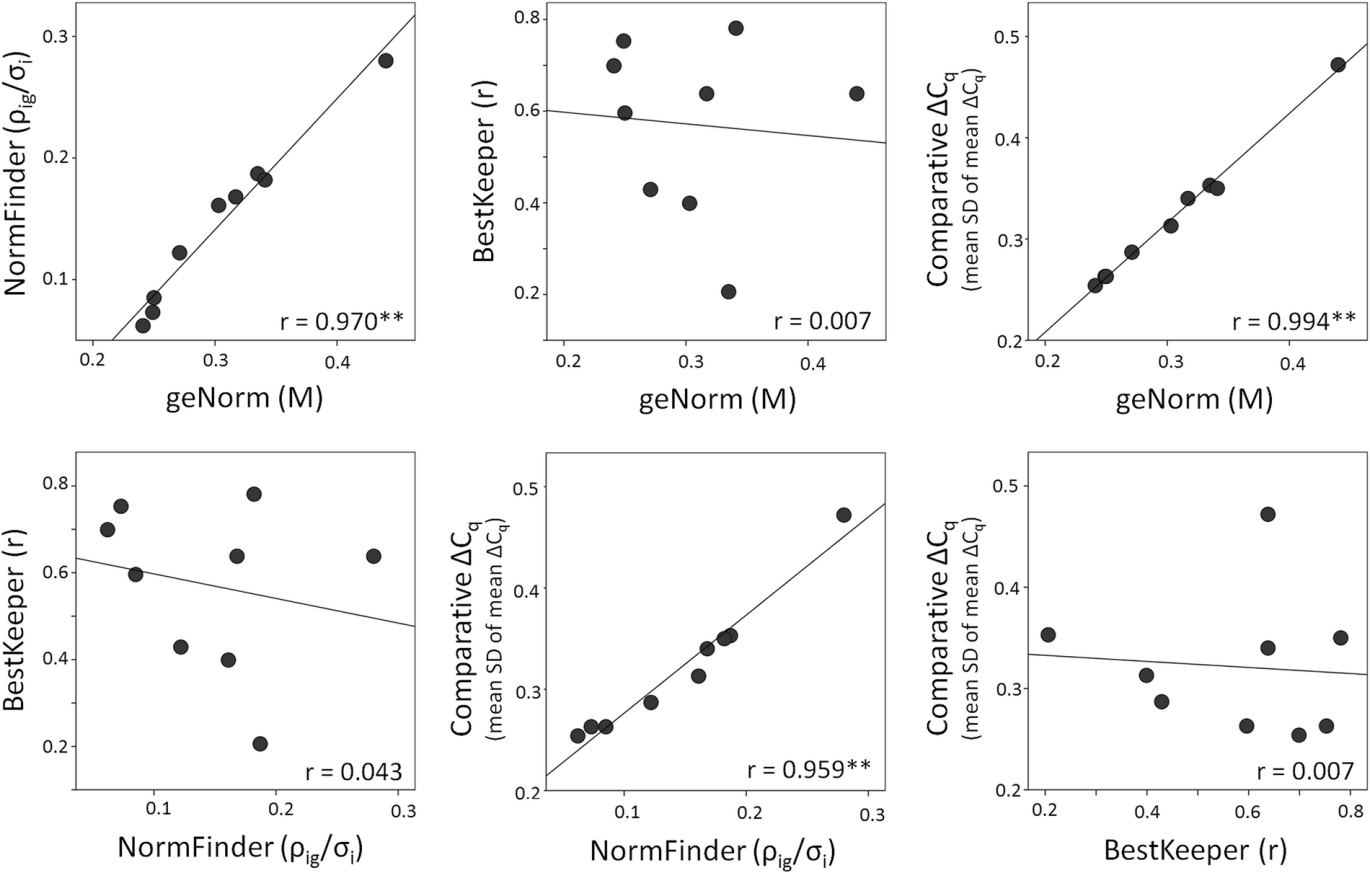
Correlation matrix of the stability values of the four different algorithms used for reference gene evaluation (geNorm, NormFinder, BestKeeper, comparative ΔC_q_). Scatterplots visualize bivariate correlations of the overall stability values of the nine assessed candidate reference genes as computed by two different algorithms including a linear regression line. r = Pearson’s correlation coefficient; **p ≤ 0.01.

Bivariate correlations of the pooled/overall (n = 18) gene ranking of the individual algorithms are presented in Fig. 4. geNorm, NormFinder and comparative ΔC_q_ showed significant and pronounced gene ranking correlations. By contrast BestKeeper ranking did not correlate significantly with the other three tested algorithms (Fig. 4).

## Discussion

In general, PPIB, TBP and RPL22 performed best as reference genes with the highest stability values and good primer and amplification efficiency and reliability throughout for all experimental conditions and algorithms (mostly ranking top three). In addition, these genes have different cellular functions thus avoiding co-regulation^7^. PPIB is a protein binding cyclosporine in the endoplasmic reticulum, which plays a major role in the folding of collagen type I^42^ and was recently found to be associated pathological conditions, such as osteogenesis imperfecta^43^, which may also affect the periodontal apparatus. In contrast, TBP is a TATA-box-binding protein, which is required for the initiation of transcription by RNA polymerase II^44^, and RPL22 is a ribosomal protein^45,46^, which is involved in the control of morphogenesis by regulating Smad2 mRNA splicing^47^. Only subtle differences in their relative stability were detected among the three genes. A notable exception is RPL22 in experiments with Agac toxins (periodontitis), ranked as more unstable by all algorithms, indicating a regulation by Agac toxins. Also in orthodontic experiments BestKeeper found RNA18S5 and YWHAZ to be more stable than RPL22, TBP and PPIB. RNA18S5 and YWHAZ, however, are not suited as reference genes for hPDL fibroblasts, as discussed later. In a previous animal study on Fisher344 rats^10^, we also found PPIB to be one of the two most stably expressed reference genes for a conglomerate of dental-periodontal tissue. The second most stable reference gene identified (YWHAZ), however, did not perform well in the present study, which could be attributable to the difference in species.

Based on the results of this study, PPIB and RPL22 or TBP are thus recommended to be used in cell culture experiments with hPDL fibroblasts isolated from young and healthy donors treated with compressive force, as they are the most stably expressed reference genes under these conditions. PPIB and TBP are most stably expressed in hPDL fibroblasts stimulated with Agac toxins and should therefore be used for *in vitro* experiments on periodontitis. Other pre-existing pathological conditions such as osteogenesis imperfecta as well as the age of hPDL donors may also affect the performance of candidate reference genes^7,48^. The results of this study can thus only be safely generalized to hPDL cells from young and generally healthy donors, whereas different gene stability rankings may be expected for hPDL cells from older donors or during pathological conditions, as evidenced by the observed reduced stability of RPL22 in experimental periodontitis.

For evaluation of reference gene stability, four algorithms were used. geNorm^29^ calculates the average pairwise C_q_ variation of a one candidate reference gene with all other genes, which is given as expression stability M. The conceptual idea behind geNorm is the supposed constancy of the expression ratio of two ideal (stable) reference genes in all samples and experimental conditions^29^. Genes with higher M values are associated with a greater average pairwise variation in gene expression and should thus be excluded for normalization, since they indicate expression ratio inconstancy and thus expression instability^29^.

Despite generally increased normalization reliability^7^, the usage of a plethora of reference genes is time- and cost-demanding. Thus it is neither practical nor common to use more reference genes than necessary^49^. A reliable identification of the minimally necessary number of reference genes without risking distinct bias on target gene expression is therefore essential and could be achieved with the geNorm algorithm by calculating the average pairwise variation between normalization factors of n and n + 1 candidate genes (V_n_/V_n+1_)^29^. Since variation was not substantial after addition of a third reference gene (cut-off value V ≤ 0.15), no additional stabilizing effect was achieved for normalization, which is why two reference genes should be sufficient for normalization throughout^29^.

The NormFinder algorithm^36^ determines intra- and intergroup variation, creating a combined stability value for each candidate reference gene using a model-based approach^36^ with lower stability values associated with higher expression stability. The comparative ΔC_q_ method compares the relative expression of gene pairs within each biological replicate and ranks reference genes according to the mean standard deviation of the mean ∆C_q_ differences of the respective gene from all other genes assessed with a lower SD indicating a more stable gene expression^31^. The underlying conceptual idea is that if ΔC_q_ values between two assessed genes show variation between different samples, the expression level of one or both genes is bound to vary^31^. By performing repeated pairwise comparisons for all candidate gene combinations, the gene pairs with least variability and thus highest stability can be determined^31^.

BestKeeper^37^ determines stability based on the standard deviation (SD) of C_q_ means of each candidate reference gene as well as Pearson’s correlation coefficient r by pairwise bivariate correlations of C_q_ values of each gene with a “BestKeeper Index” as geometric mean of the individual C_q_ values of all reference genes with SD ≤ 1 (genes with SD > 1 are excluded as unsuitable/unstable). Higher r values, indicating a higher contribution of the respective gene to the “Index”, can thus be interpreted as more stably expressed genes.

The different stability values for individual candidate reference genes show that both orthodontic force application as well as bacterial Agac toxins have a distinct influence on the gene expression of basic cell metabolism, confirming that complex cellular-biological processes occur during both conditions directly affecting basic cell metabolism at a transcriptional level^2,3,5,10^. Interestingly, some of the more popular reference genes used previously, particularly in orthodontic and periodontitis experiments on hPDL fibroblasts^12–17^, performed with lower stability than anticipated. These include traditional and often used reference genes such as β-actin^33^ (ACTB), glucuronidase beta (GUSB), ubiquitin C (UBC) and tubulin beta class I (TUBB), for which no specific primer pair could be constructed according to the MIQE guidelines^11^. Several of them are commercially available, but these primers not in line with MIQE guidelines, as was the case with the comparatively tested primer pair for TUBB, which was gene-specific, but not intron-spanning/-flanking, allowing a co-amplification of genomic DNA, if no DNAse treatment is performed. Since sequences of these commercially available primers are often not published for corroboration, other problems such as secondary structure formation of primers and amplicons at annealing might be present as well, which is why we chose to exclude these genes and primers in our principal analysis. Furthermore the frequently used reference genes 18S-rRNA (RNA18S5)^8^, POLR2A^15–17,50^ and GAPDH^13,14^ also showed limited expression stability. Thus the usage of these common reference genes should be reconsidered in future gene expression studies on hPDL fibroblasts. In addition, both RNA18S5 and EEF1A1 show quite high absolute expression levels, which could pose a problem in relative quantification, since the expression levels of reference genes should approximate those of target genes for reliable results^7^. The usage of ribosomal RNA genes as reference has also been discouraged due to various other associated problems^7^. EEF1A1 did perform well in gene rankings for individual experimental groups, but showed both the highest technical as well as biological variation among all genes tested. YWHAZ also showed high biological variation and reduced stability, limiting its suitability.

The results indicate that RNA samples were of sufficient quality for RT-qPCR analysis. High intraassay and biological reliability as well as sufficient precision of the obtained data^39^ could be confirmed. Protein-free and intact RNA were indicated by purity and integrity assessment of total RNA. If protein contamination is present, it could result in an inhibition of the reverse transcription and qPCR reaction, thus leading to biased C_q_ values^40^. Primer efficiency (from standard curve^11,37^) ranged between 91.7% and 100.3% and amplification efficiency (from individual kinetic curves^51^, LinRegPCR^52–54^) between 83.2% and 119.3%. Thus formation of primer dimers, which can cause an efficiency beyond 100%^39^, was mostly at an acceptable level. Only for RNA18S5 we found primer and amplification efficiencies over 100%. This overestimation of efficiency can most likely be attributed to inhibitor traces in the RNA sample, which were further diluted for efficiency analyses, as well as the very low C_q_ values for RNA18S5, which make RNA18S5 an unsuitable reference gene, since absolute expression of reference genes should be similar to that of target genes^7,10^. Amplification efficiencies distinctly below 90% for POLR2A and RPLP0 also indicate limited suitability of these genes for normalization. Primer specifity as confirmed *in silico* and *in vitro* successfully prevented the co-amplification of pseudo-genes and homologues.

Although various studies on other tissues and species also used several algorithms to assess reference gene stability^52,55,56^, others only considered one or two algorithms^27,34^. We thus comparatively examined the various available statistical stability algorithms regarding their conformity to determine, whether the combined usage of several algorithms has advantages in reference gene stability determination. Our study showed a significant and high correlation between geNorm, NormFinder and comparative ΔC_q_ algorithms as confirmed by the similar stability rankings of genes observed, which indicate that these could be used interchangeably. However, no significant correlations were found with the BestKeeper algorithm. Several reasons for this discrepancy of BestKeeper rankings to the rankings produced by the other algorithms can be assumed. BestKeeper was not particularly created to produce rankings of reference genes, but rather focuses on general suitability in a sequential two-step assessment (standard deviation of mean C_q_ and then correlation coefficient r). The three similarly performing algorithms are based on either performing pairwise comparisons of individual candidate reference genes with linear quantities (geNorm)/raw ΔC_q_ values (comparative ΔC_q_ method), ranking genes according to their expression profile similarity^36^, or a model-based approach using linear quantities (NormFinder), which is considered more robust, since it is less influenced by co-regulation of candidate reference genes^36^. By contrast, BestKeeper performs correlations of candidate genes with a single “BestKeeper Index”, identical for all correlations and not created from all candidate genes, but a selection hereof, pre-excluding those with higher C_q_ standard deviations than 1. Furthermore BestKeeper is based on raw C_q_ values instead of linear quantities, which are used by geNorm and NormFinder.

## Conclusion

Using four different mathematical algorithms (geNorm, NormFinder, comparative ΔC_q_ and BestKeeper) PPIB, TBP and RPL22 were identified as the most stable, reliable and suitable reference genes for normalization in relative RT-qPCR gene expression studies on human periodontal ligament fibroblasts, particularly in studies on orthodontic tooth movement (PPIB/RPL22) and periodontitis (Aggregatibacter actinomycetemcomitans, PPIB/TBP). Two reference genes were found to be sufficient for reliable normalization throughout. Many traditional and frequently used reference genes such as RNA18S5, POLR2A or GAPDH showed limited suitability and should be avoided in future experiments. The same is true for reference genes, for which no specific primers could be designed according to pre-specified quality criteria as described by the MIQE guidelines (ACTB, GUSB, UBC, TUBB). BestKeeper produced distinctly different stability rankings compared to the other algorithms, thus suggesting a rank-sum approach for stability evaluation.

## Materials and Methods

### *In vitro* cell culture experiments

Primary human periodontal ligament (hPDL) fibroblasts were cultivated from periodontal connective tissue isolated from the middle root section of human teeth free of decay, which had been freshly extracted for medical reasons at the authors’ dental facility. A pool of hPDL cell lines from four different patients was used (1 male, 3 female, age: 16–23 years). Collection and usage of hPDL fibroblasts from discarded patient biomaterial were approved by the ethics committee of the University of Regensburg, Germany (approval number 12-170-0150), and all experiments were carried out in accordance with the relevant guidelines and regulations. Informed consent was obtained from all participants and/or their legal guardian/s. Tissue samples were grown in 6-well cell-culture-plates until proliferation of adherently growing hPDL fibroblasts under normal cell culture conditions (37 °C, 5% CO_2_, water-saturated) in full media consisting of DMEM high glucose (D5796, Sigma–Aldrich^®^, Munich, Germany), 10% FCS (P30–3306, PAN-Biotech, Aidenbach, Germany), 1% L-glutamine (SH30034.01, GE-Healthcare-Europe, Munich, Germany), 100 µM ascorbic acid (A8960, Sigma-Aldrich^®^) and 1% antibiotics/antimycotics (A5955, Sigma-Aldrich^®^). hPDL fibroblasts were identified by their spindle-shaped morphology and hPDL-specific marker gene expression^12,57,58^ (Supplementary Table 5 and Supplementary Fig. 1). Until use they were then frozen in liquid nitrogen (90% FCS, 10% DMSO, freezing 1 °C/minute).

**Figure 5:**
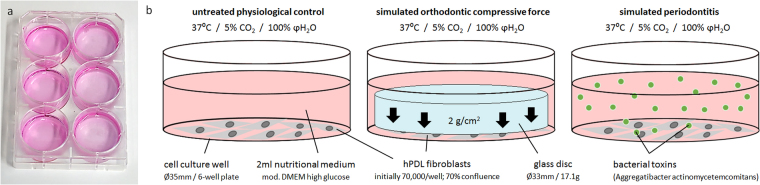
Experimental setup for the hPDL fibroblast experiments. (**a**) 6-well cell culture plate with untreated controls and simulated periodontitis (left side) as well as simulated orthodontic compressive force (right side). (**b**) Experimental conditions tested (physiological cell culture conditions): untreated physiological controls (adherently growing hPDL cells at 70% confluence in full medium), simulated orthodontic compressive force of 2 g/cm^2^ applied by a 17.1 g glass disc, simulated periodontitis by adding bacterial lysate (toxins) of Aggregatibacter actinomycetemcomitans to the medium.

hPDL fibroblasts of the 6^th^ passage^24^, quantified with Beckman Coulter Counter Z2™ (Beckman Coulter GmbH, Krefeld, Germany) according to the manufacturer’s recommendations, were randomly seeded onto 6-well cell-culture-plates at an initial density of 70,000 cells per well (Fig. 5a). We prepared three different experimental groups at 70% confluency (Fig. 5b) (37 °C, 5% CO_2_, 100% water-saturated, 2 ml DMEM/well) with 6 biological replicates (samples) each:

- Untreated (physiological conditions, n = 6) - incubated for 48 h;
- Compressive orthodontic force (n = 6) - mechanical stimulation for 24 h (2 g/cm^2^ pressure, sterilized glass plate of defined weight and size) according to Kanzaki *et al*.^3^ and Kirschneck *et al*.^15,16^ after a pre-incubation phase of 24 h;
- Periodontitis (n = 6) - according to Proff *et al*.^17^, hPDL fibroblasts were incubated for 48 h with bacterial lysate of heat-inactivated *Aggregatibacter actinomycetemcomitans* (Agac, 10^7^ cells/ml, DSM11123, German Collection of Microorganisms and Cell Cultures, Braunschweig, Germany), which was prepared as described before^12,17^.

### Isolation and purity assessment of total RNA

After washing the hPDL fibroblasts twice with phosphate-buffered saline, total RNA was extracted by applying peqGOLD TriFast™ (1 ml/well, PEQLAB-Biotechnology GmbH, Erlangen, Germany) and further processing according to the manufacturer’s instructions^10,15,16^. No DNAse treatment was performed, as all used primers were intron-flanking. We eluted the resulting RNA pellet in nuclease-free water (25 µl, T143, Carl-Roth GmbH, Karlsruhe, Germany) with immediate cooling on ice. To assess purity and quantity of the eluted total RNA, we determined optical density (OD) photometrically at 280 nm and 260 nm (NanoDrop ND-2000, Thermo-Fisher Scientific Inc., Waltham, MA, USA) with 1 OD_260nm_ equalling 40 ng/µl total RNA^10^. An OD_260nm/280nm_ ratio of >1.8 was considered protein-free RNA^27,40^. Mean concentration of extracted RNA (n = 18) was calculated from its optical density at 260 nm obtained with NanoDrop as 358.2 ng/µl (SD 104.7/Min. 218.6/Max. 495.4 - divergent results in capillary electrophoresis, Supplementary Data 2) with a mean NanoDrop OD_260nm/280nm_ ratio of 1.90 (SD 0.03/Min. 1.82/Max. 1.96).We measured RNA integrity with an Agilent 2100 Bioanalyzer (Agilent Technologies Inc. Santa Clara, CA, USA) according to the manufacturer’s protocol. RIN values ranged from 9.50 to 10 (mean 9.85, SD 0.15) indicating absence of RNA degradation^59^ (Supplementary Data 2). Integrity of total RNA was confirmed by the non-proprietary 28 S/18 S ratio of ribosomal RNA in gel electrophoresis, which ranged from 1.6 to 1.8 (mean 1.71, SD 0.09; Supplementary Data 2). Reverse transcription negative control (-RT) and negative NTC reactions confirmed sufficient absence of genomic DNA, contamination and primer dimers with measured C_q_ values substantially higher than those of target samples (Supplementary Table 1).

### Reverse transcription (cDNA synthesis)

To synthesize cDNA, we transcribed a standardized quantity of 1 µg RNA per sample using a random hexamer primer (0.1 nmol, 1 µl, SO142, Life Technologies GmbH, Darmstadt, Germany), an oligo-dT18 primer (0.1 nmol, 1 µl, SO131, Life Technologies), 5 × M-MLV-buffer (4 µl, M1705, Promega, Fitchburg, WI, USA) and dNTP mix (40 nmol, 1 µl–10 nmol/dNTP, Roti^®^-Mix PCR3, L785.2, Carl-Roth GmbH) ad 20 µl nuclease-free H_2_O (T143, Carl-Roth GmbH). After incubation (3 min, 70 °C) the mixture was quickly cooled on ice. We then added reverse transcriptase (200 U, 1 µl, M1705, Promega) and an RNase inhibitor (40 U, 1 µl, EO0381, Life Technologies), continued incubation (37 °C, 60 min) and heat-inactivated the reverse transcriptase (95 °C, 2 min). To minimize experimental variations, synthesis of cDNA, which was stored at −20 °C until use, was performed concurrently for all samples.

### Quantitative real-time polymerase chain reaction (RT-qPCR)

Primer design was based on the official gene nucleotide sequences from the NCBI Nucleotide database (GeneBank, National Centre for Biotechnology Information, Bethesda MD, USA). They were constructed with NCBI PrimerBLAST^38^ considering the final concentration of qPCR components according to optimized criteria^11,39–41^. Primers received no terminal or other modifications and were synthesized and purified by Eurofins MWG Operon LLC (Huntsville, AL, USA; High Purity Salt Free Purification HPSF^®^). For qPCR amplification we used a Mastercycler^®^ ep realplex-S thermocycler (Eppendorf AG, Hamburg, Germany) in conjunction with 96 well PCR plates (TW-MT, 712282, Biozym Scientific GmbH, Hessisch Oldendorf, Germany) and BZO Seal Filmcover sheeting (712350, Biozym Scientific GmbH). Into each well SYBR^®^Green JumpStart™ Taq ReadyMix™ (7.5 µl, Sigma–Aldrich^®^, S4438), consisting of Tris–HCl (20 mM, pH 8.3), KCl (100 mM), MgCl_2_ (7 mM), dNTPs (0.4 mM per dATP, dCTP, dGTP, dTTP), stabilizers, Taq-DNA-polymerase (0.05 U/µl), JumpStart™ Taq antibody and SYBR^®^Green I, as well as the respective cDNA solution (1.5 µl, dilution 1:10) and the respective primer pair (7.5 pmol, 0.75 µl–3.75 pmol/primer) were pipetted ad 15 µl nuclease-free H_2_O (T143, Carl-Roth GmbH). We amplified the cDNA in triplets (three technical replicates) per candidate reference gene and biological replicate (sample) and on the same qPCR plate in 45 cycles (initial heat activation 95 °C/5 min, per cycle 95 °C/10 s denaturation, 60 °C/8 s annealing, 72 °C/8 s extension, Supplementary Data 3), resulting in 6 (samples) × 3 (experimental conditions) × 3 (technical replicates) analysed PCR reactions (C_q_ values) per candidate reference gene. At the end of each extension step SYBR^®^Green I fluorescence was measured at 521 nm.

### Amplification and primer efficiency and validation

RT-qPCR efficiency over all samples (n = 18)^37^ was calculated both sample-specific^51^ (amplification efficiency E_A_, LinRegPCR software^53,54,60^, http://LinRegPCR.HFRC.nl) and factor-specific (primer efficiency E_P_^61^) according to the MIQE guidelines^11,39,40^. For primer efficiency determination a 6x log_10_ serial dilution series of a random cDNA sample (untreated group) was amplified in triplet (three technical replicates per dilution level) for each candidate reference gene and the limit of detection (LOD) as the highest dilution, at which 95% (all three) of the technical replicates are detectable, was determined. Standard curves were created by linear regression of the resulting C_q_ values with the relative cDNA dilution (Supplementary Data 4) within the linear dynamic range (LDR) and the corresponding coefficients of determination r^2^ as well as qPCR primer efficiencies (E_P_) derived from the slope of the standard curve: E_P_ = (10^−1/slope^ − 1) × 100%. Only primer pairs with a linear relation between C_q_ and the log-transformed cDNA copy number (r^2^ > 0.98) were considered as possible valid reference gene candidates^39^. In addition, only efficiencies E within the range of 90–110% were deemed acceptable. Specific amplification of target reference genes was assessed by agarose gel electrophoreses (single band, correct size)^39^ and a specific peak in melting curve analysis (95 °C/15 s, 60 °C/15 s, then continuous temperature increase to 95 °C with fluorescence measurement for 20 min, Supplementary Data 3). For agarose gel electrophoresis each qPCR product (7 µl) was mixed with sucrose loading buffer (3 µl) and loaded on a 1.5% agarose gel, which was prepared with Gel Red (6 µl, 41003, Biotrend Chemikalien GmbH, Köln, Germany). The amplification products were separated parallel to a 100 bp DNA ladder at 120 V for 40 min in TAE buffer. Fluorescent bands were visualized by the gel documentation system Genoplex 2 and its software GenoSoft (VWR International GmbH, Darmstadt, Germany). Intraassay reliability was assessed per gene via the standard deviation of the arithmetic mean of C_q_ of the technical replicates (triplets) across all biological replicates (samples). Repeatability was deemed sufficient, if maximum SD was <0.553 C_q_^10^. For each primer pair and qPCR run we also tested a no-template-control (NTC) without cDNA and a -RT control (cDNA synthesis without reverse transcriptase added) on the same plate to exclude possible bias by primer dimers, contaminating or genomic DNA.

### Data analysis and statistics

C_q_ values, defined as the second derivative maximum of the fluorescence signal curve, were calculated with the realplex software (version 2.2, Eppendorf AG, CalqPlex algorithm, Automatic Baseline, Drift Correction On). An arithmetic mean of each C_q_ triplett per gene and sample was used for further analysis. The stability of each candidate gene was calculated with four different mathematical algorithms: geNorm^29^, NormFinder^36^, BestKeeper^37^ and the comparative ΔC_q_ method^31^. Stability calculations were done with the official Microsoft-Excel-based software applets for geNorm, NormFinder and BestKeeper according to developers’ instructions. For the comparative ΔC_q_ method manual calculations were performed^31^. The geNorm and NormFinder algorithms require the transformation of the raw C_q_ data to linear scale expression quantities Q^10,26^ corresponding to the qPCR (primer) efficiency (E) of each gene: Q = E_P_^−(Cqmin-Cqsample)^. The genes were ranked according to their stability values (geNorm: M, NormFinder: ρ_ig_/σ_i_, deltaCT: mean SD of ∆C_q_; BestKeeper: Pearson’s r) for each algorithm and experimental condition as well as combined conditions and a rank sum of all algorithms calculated per gene for final stability assessment with the smallest rank sum indicating the most stable reference gene (Table 3). The geNorm algorithm allowed a calculation of the ideal number of reference genes for reliable RT-qPCR normalization^29^. If pairwise variation (V_n_/V_n + 1_) between two sets of reference genes with one set including an additional reference gene was ≤0.15, this additional gene was deemed unnecessary for normalization (Fig. 3a). To assess ranking variations between algorithms, we used IBM SPSS Statistics^®^ 23 (IBM, Armonk, NY, USA) to create a correlation matrix of bivariate correlations (Pearson´s correlation coefficient r, two-sided, normality confirmed by Shapiro-Wilk tests and histogram evaluation) of the overall pooled stability values as calculated by two respective algorithms.

### Data availability statement

All datasets are publically available either as supplementary information to this article or upon request from the corresponding author. RT-qPCR experiments are in agreement with the MIQE (Minimum Information for Publication of Quantitative Real-Time PCR Experiments) guidelines^11^. The MIQE checklist (http://www.rdml.org/miqe) is provided as supplementary information (Supplementary Table 1).

## Electronic supplementary material

Supplementary Information
