# Supplementary material for: Valid gene expression normalization by RT-qPCR in studies on hPDL fibroblasts with focus on orthodontic tooth movement and periodontitis
Source: Sci Rep. 2017 Nov 7;7:14751. doi: 10.1038/s41598-017-15281-0 (PMC5677027; doi:10.1038/s41598-017-15281-0)
Supplement: Supplementary file 1 — Supplementary Information [file 41598_2017_15281_MOESM1_ESM.pdf]

Valid gene expression normalization by RT-qPCR in studies on hPDL fibroblasts with focus on orthodontic tooth movement and periodontitis

Christian Kirschneck[1,*], Sarah Batschkus[2], Peter Proff[1], Josef Köstler[3], Gerrit Spanier[4], and Agnes Schröder[1]

[1]Department of Orthodontics, University Medical Centre of Regensburg, Regensburg, D-93053, Germany

[2]Department of Orthodontics, University of Goettingen, Goettingen, D-37075, Germany

[3]Institute of Microbiology and Hygiene, University Medical Centre of Regensburg, Regensburg, D-93053, Germany

[4]Department of Cranial and Maxillo-Facial Surgery, University Medical Centre of Regensburg, Regensburg, D-93053, Germany

*christian.kirschneck@ukr.de; Tel.: +49 941 944 6093; Fac.: +49 941 944 6169

Supplementary Information

Contents

Page

Supplementary Table 1 – MIQE checklist ………………………………………………………………………….. 3

Supplementary Table 2 – RNA quantity and quality ………………………………………………………………. 10

Supplementary Table 3 – Raw C_q values of RT-qPCR ………………………………………………………… 11

Supplementary Table 4 – Gene stability ranking for individual experimental groups …………………………. 12

Supplementary Table 5 – Marker genes, primers and amplicons used for hPDL characterization ………….. 13

Supplementary Figure 1 – Characterisation of hPDL fibroblasts ……………………………………………….... 14

Supplementary Figure 2 – Uncropped original gel of RT-qPCR products (amplification specifity) ………….. 15

Supplementary Data 1 – Splice variants and secondary structure analysis of amplicons and primers …….. 16

Supplementary Data 2 – RNA integrity ……………………………………..…………………………….... 60

Supplementary Data 3 – Amplification plot and Melting curve analysis (RT-qPCR) ………………………….. 86

Supplementary Data 4 – qPCR primer efficiency ………………………………..…………………………….. 92

Supplementary Data 5 – TUBB RT-qPCR, specifity and efficiency ……………………………………………... 101

Supplementary Table 1. MIQE checklist for authors, reviewers and editors. E = essential information; D = desirable information.

Item to check	Importance	Description how item was addressed in study / article
**Experimental design**		
Definition of experimental and control groups	E	Control group: untreated hPDL fibroblasts (physiological conditions); Experimental groups: hPDL fibroblasts treated with compressive orthodontic force (model for orthodontic tooth movement) or bacterial lysate of Aggregatibacter actinomycetemcomitans (Agac, model for bacterial periodontitis) for 24h. For details see materials and methods and Figure 5.
Number within each group	E	n = 6
Assay carried out by the core or investigator´s laboratory?	D	All assays were carried out in investigators' laboratory.
Acknowledgment of authors´ contributions	D	C.K. conceived the idea of the study/study design as well as designed/validated the used primer pairs. S.B., P.P. and A.S. contributed to discussion and study design. A.S. and C.K. conducted the experiments. A.S., C.K. and S.B. analysed the results. J.K. produced and contributed the Agac bacterial lysate. G.S. provided the primary hPDL fibroblasts. C.K. and A.S. wrote the manuscript and created the figures, tables and the supplementary material. All authors reviewed the manuscript.
**Sample**		
Description	E	Primary human periodontal ligament fibroblasts (hPDL) were cultivated from periodontal connective tissue isolated from the middle root section of human teeth free of decay, which had been freshly extracted for medical reasons. A pool of hPDL cell lines from four different patients was used (1 male, 3 female, age: 16-23 years). Cells were identified by means of hPDL-specific marker gene expression and their spindle-shaped morphology (Supplementary Table 5 and Supplementary Figure). Ethical consent was obtained from the local ethics committee (12-170-0150).
Volume/mass of sample processed	D	Varying size of tissue sample / number of hPDL fibroblasts extracted. 70.000 cells were finally seeded per well / biological replicate for the experiments.
Microdissection or macrodissection	E	Microdissection
Processing procedure	E	Tissue samples were grown in 6-well cell culture plates until proliferation of adherently growing hPDL under normal cell culture conditions (37°C, 5% CO_2, water-saturated) in full media, then trypsinized and further cultivated and passaged until the 6th passage.
If frozen, how and how quickly?	E	Until use hPDL fibroblasts were frozen in liquid nitrogen (90% FCS, 10% DMSO, freezing 1°C/minute in cryo-box with isopropanol).
If fixed, with what and how quickly?	E	Not fixed.

Sample storage conditions and duration	E	Samples were directly isolated and cultivated under cell culture conditions in cell culture flasks and plates (37°C, 5% CO_2, water-saturated) in full media consisting of DMEM high glucose (D5796, Sigma–Aldrich®, S4438, St. Louis, MI, USA), 10% FCS (P30-3306, PAN-Biotech, Aidenbach, Germany), 1% L-glutamine (SH30034.01, GE Healthcare Europe, Munich, Germany), 100 µM ascorbic acid (A8960, Sigma-Aldrich, Munich, Germany) and 1% antibiotics/antimycotics (A5955, Sigma–Aldrich®, S4438).
**Nucleid acid extraction**		
Procedure and/or instrumentation	E	After washing the cells twice with sterile phosphate-buffered saline, total RNA from hPDL cells was extracted by applying peqGOLD TriFast™ and further processing according to the manufacturer's instructions. We eluted the resulting RNA pellet in nuclease-free water (25µl) with immediate ice-cooling.
Name of kit and details of any modifications	E	peqGOLD TriFast™ (1 ml / well, PEQLAB Biotechnology GmbH, Erlangen, Germany). We followed the manufacturer´s protocol exactly.
Source of additional reagents used	D	Chloroform (EMSURE®, 1.02445.1000; Merck KGaA, Darmstadt, Germany), 2-Propanol (20842.330, VWR International GmbH, Darmstadt, Germany), Ethanol (32205, Sigma-Aldrich, Munich, Germany); RNase-free water (T143, Bioscience-Grade, Carl Roth GmbH & Co. KG, Karlsruhe, Germany)
Details of DNase or RNase treatment	E	1 µg of RNA was treated with 40 U of RNase inhibitor (EO0381, Life Technologies) in a 22 µl final volume for cDNA synthesis. No DNAse treatment was performed.
Contamination assessment (DNA or RNA)	E	For each primer pair and qPCR run we also tested a no-template-control (NTC) without cDNA and a -RT control (cDNA synthesis without enzyme reverse transcriptase added) on the same plate to exclude possible bias by primer dimers, contaminating or genomic DNA.
Nucleic acid quantification	E	RNA concentration was determined by measuring the absorbance at 260 nm UV light with 1 OD_{260nm} equalling 40 ng/µl total RNA. OD = optical density
Instrument and method	E	NanoDrop ND-2000 UV spectrophotometer (Thermo Fisher Scientific, Waltham, MA, USA)
Purity (A260/A280)	D	RNA purity was determined by measuring the absorbance ratio $OD_{260nm/280nm}$ as well as $OD_{260nm/230nm}$. An $OD_{260nm/280nm}$ ratio of >1.8 was considered protein-free RNA, and an $OD_{260nm/230nm}$ ratio of >2.0 phenol-/ethanol-free RNA (Supplementary Table 2).
Yield	D	RNA yield was calculated as the amount of RNA obtained (µg) per well. **Mean yield:** 358.2 ng/µl x 2000 µl/well = 716.4 µg/well; **Min./Max. yield:** 218.6 / 495.4 ng/µl x 2000 µl/well = 437.2 / 990.8 µg/well (Supplementary Table 2).
RNA integrity: method/instrument	E	RNA integrity was determined with an Agilent 2100 Bioanalyzer (Agilent Technologies Inc. Santa Clara, CA, USA) according to the manufacturer's protocol (Supplementary Data 2).
RIN/RQI or C_q of 3´ and 5´ transcripts	E	RIN values ranged from 9.5 to 10 (mean 9.85, SD 0.15), indicating an absence of RNA degradation (Supplementary Data 2).
Electrophoresis traces	D	Electrophoresis traces were determined with an Agilent 2100 Bioanalyzer (Agilent Technologies Inc. Santa Clara, CA, USA) according to the manufacturer's protocol (Supplementary Data 2).

Inhibition testing (C_q dilutions, spike, or other)	E	For evaluation of qPCR and primer efficiency as well as absence of inhibitors a $\log_{10}$ serial dilution series of a random cDNA sample from the untreated group was amplified in triplet for each candidate reference gene and the limit of detection (LOD) as the highest dilution, at which 95% (all three) of the technical replicates are detectable (C_q values), was determined. A standard curve was created by linear regression of the resulting C_q values with the relative dilution within the linear dynamic range (LDR) and the coefficient of determination r^2 as well as qPCR reaction efficiencies (E) with 95% confidence intervals were determined from the slope of the standard curve: E = ($10^{-1/slope}$-1) x 100%. Only primer pairs with a linear relation between C_q and log-transformed cDNA copy number (r^2 >0.98) were considered as possible valid reference gene candidates. In addition, only efficiencies E within the range of 90-110% were deemed acceptable. (Table 2, Supplementary Data 4)
**Reverse transcription**		
Complete reaction conditions	E	To synthesize cDNA, we transcribed a standardized quantity of 1µg RNA per sample using a random hexamer primer (0.1 nmol, 1 µl, SO142, Life Technologies), an oligo-dT18 primer (0.1 nmol, 1 µl, SO131, Life Technologies, Thermo Fisher Scientific Inc.), 5× M-MLV-buffer (4 µl, M1705, Promega, Fitchburg, WI, USA) and dNTP mix (40 nmol, 1 µl, 10 nmol/dNTP, Roti®-Mix PCR3, L785.2) ad 20 µl nuclease-free H_2O (Roth BioScience Grade T143, Carl Roth GmbH & Co. KG). After incubation for 3 min at 70°C the mixture was quickly cooled on ice (RNA denaturation). We then added reverse transcriptase (200 U, 1 µl, M1705, Promega) and an RNase inhibitor (40 U, 1 µl, EO0381, Life Technologies), continued incubation at 37°C for 60 min and inactivated the reverse transcriptase by heat (95°C, 2 min). To minimize experimental variations, synthesis of cDNA, which was stored at −20°C until use, was performed concurrently for all samples.
Amount of RNA and reaction volume	E	**Amount of RNA:** 1 µg; **Reaction volume:** 22 µl
Priming oligonucleotide (if using GSP) and concentration	E	0.1 nmol random hexamer primer; 0.1 nmol oligo-dT18 primer
Reverse transcriptase and concentration	E	Reverse transcriptase (200 U, 1 µl, M1705, Promega) in a final concentration of 9.1 U/µl (200 U / 22 µl)
Temperature and time	E	3 min at 70°C; 60 min at 37°C; 2 min at 95°C
Manufacturer of reagents and catalogue numbers	D	Specified in “Complete reaction conditions”.
C_q with → without reverse transcription	D	The signal of the amplification plot without reverse transcriptase was very late and there was a high C_q value difference between the -RT control and all cDNA samples.<br>**GAPDH:** 15→29; **PPIB:** 16→34; **YWHAZ:** 22→32; **POLR2A:** 21→none; **TBP:** 23→35; **EEF1A1:** 14→27; **RPLP0:** 17→32; **RNA18S5:** 9→30; RPL22: 18→30
Storage conditions of cDNA	D	-20°C
**qPCR protocol**		
Complete reaction conditions	E	For qPCR amplification we used a Mastercycler® ep realplex-S thermocycler (Eppendorf AG, Hamburg, Germany) in conjunction with 96 well PCR plates (TW-MT, 712282, Biozym Scientific GmbH, Hessisch Oldendorf, Germany) and BZO Seal Filmcover sheeting (712350, Biozym Scientific GmbH). Into each well SYBR®Green JumpStart™ Taq ReadyMix™ (7.5 µl, Sigma–Aldrich®, S4438, St. Louis, MI, USA), consisting of Tris–HCl (20 mM, pH 8.3), KCl (100 mM), $MgCl_2$ (7 mM), dNTPs (0.4 mM per dATP,

		dCTP, dGTP, dTTP), stabilizers, Taq-DNA-polymerase (0.05 U/µl), JumpStart Taq antibody and SYBR Green I, as well as the respective cDNA-solution (1.5 µl, dilution 1:10) and the respective primer pair (7.5 pmol, 0.75 µl - 3.75 pmol/primer) were pipetted ad 15 µl nuclease-free H_2O (BioScience Grade T143, Carl Roth GmbH & Co. KG). A master-mix of all components except the cDNA solution was created to minimize technical errors during manual pipetting. We then amplified the cDNA in triplets (technical replicates) per candidate reference gene in 45 cycles (initial heat activation 95°C/5 min, per cycle 95°C/10 s denaturation, 60°C/8 s annealing, 72°C/8 s extension). At the end of each extension step SYBR Green I fluorescence was measured at 521 nm. For each biological replicate all genes were amplified in triplet on the same qPCR plate to minimize biasing effects of possible inter-run variations on relative reference gene stability assessment.
Reaction volume and amount of cDNA/DNA	E	**Reaction volume:** 15 µl; **Amount of cDNA:** 1.5 µl of an 1:10 dilution of the cDNA stock solution
Primer, (probe), Mg_2, and dNTP concentrations	E	3.75 pmol/primer; 3.5 mM $MgCl_2$; 0.2 mM dNTP; 50 mM KCl
Polymerase identity and concentration	E	Taq-DNA polymerase in a final concentration of 0.025 U/µl (SYBR®Green JumpStart™ Taq ReadyMix™, Sigma–Aldrich®, S4438, St. Louis, MI, USA)
Buffer/kit identity and manufacturer	E	SYBR®Green JumpStart™ Taq ReadyMix™ (Sigma–Aldrich®, S4438, St. Louis, MI, USA)
Exact chemical composition of the buffer	D	20 mM Tris–HCl, pH 8.3, final concentration 10 mM
Additives (SYBR Green I, DMSO, and so forth)	E	SYBR Green I, stabilizers, JumpStart Taq antibody, KCl , $MgCl_2$
Manufacturer of plates/tubes and catalogue number	D	96 well PCR plates (TW-MT, 712282, Biozym Scientific GmbH, Hessisch Oldendorf, Germany) in combination with BZO Seal Filmcover sheeting (712350, Biozym Scientific GmbH)
Complete thermocycling parameters	E	Initial heat activation 95°C/5min; per cycle 95°C/10s denaturation, 60°C/8s annealing, 72°C/8s extension
Reaction setup (manual/robotic)	D	manual
Manufacturer of qPCR instrument	D	Mastercycler® ep realplex-S thermocycler (Eppendorf AG, Hamburg, Germany)
**qPCR validation**		
Evidence of optimization	D	Primer optimization is evidenced by melting curve analysis and agarose gel electrophoresis (specifity), qPCR efficiency, technical reliability and in silico secondary structure analysis of primers and amplicons. Melting temperatures T_m of primers as validated by the manufacturer Eurofins MWG Operon LLC (Huntsville, AL, USA; High Purity Salt Free Purification HPSF®) are provided in Table 1.
Specifity (gel, sequence, melt or digest)	E	Specific amplification of target reference genes was assessed by agarose gel electrophoreses (single band, correct size) and a specific peak in melting curve analysis (95°C for 15s, 60°C for 15s, then continuous temperature increase to 95°C and fluorescence measurement for 20 min). For each primer pair and qPCR run we also tested a no-template-control (NTC) without cDNA and a -RT control (cDNA synthesis without enzyme reverse transcriptase added) on the same plate to exclude possible bias by unspecific amplification (primer dimers, contaminating or genomic DNA). (Figure 1, Supplementary Data 3).

For SYBR Green I, C_q of the NTC	E	The signal of the amplification plot during efficiency analysis for standard curve generation was very late and there was a high C_q value difference between the negative control and all cDNA dilutions. **GAPDH:** 40; **PPIB:** 36; **YWHAZ:** none; **POLR2A:** 37; **TBP:** none; **EEF1A1:** none; **RPLP0:** 40; **RNA18S5:** 35; **RPL22:** none. (Supplementary Data 4)
Calibration curves with slope and y intercept	E	**GAPDH:** y=1E+9$e^{-0.659x}$, slope: -3.480; **PPIB:** y=5E+9$e^{-0.651x}$, slope: -3.508; **YWHAZ:** y=6E+9$e^{-0.651x}$, slope: -3.488; **POLR2A:** y=4E+10$e^{-0.651x}$, slope: -3.520; **TBP:** y=3E+12$e^{-0.649x}$, slope: -3.538; **EEF1A1:** y=7E+9$e^{-0.685x}$, slope: -3.315; **RPLP0:** y=2E+9$e^{-0.646x}$, slope: -3.509; **RNA18S5:** y=2E+6$e^{0.677x}$, slope: -3.319; **RPL22:** y=1E+10$e^{-0.671x}$, slope: -3.403. (Supplementary Data 4)
PCR efficiency calculated from slope	E	**GAPDH:** 93.8%; **PPIB:** 92.8%; **YWHAZ:** 93.5%; **POLR2A:** 92.3%; **TBP:** 91.7%; **EEF1A1:** 100.3%; **RPLP0:** 92.7%; **RNA18S5:** 100.1%; **RPL22:** 96.7%. (Table 2, Supplementary Data 4)
CIs for PCR efficiency or SE	D	CIs of qPCR efficiencies E were calculated for all genes tested and are given in Supplementary Data 4.
R^2 of calibration curve	E	**GAPDH:** 0.9998; **PPIB:** 0.9996; **YWHAZ:** 0.9993; **POLR2A:** 0.9984; **TBP:** 0.9974; **EEF1A1:** 0.9951; **RPLP0:** 0.9992; **RNA18S5:** 0.9974; **RPL22:** 0.9949. (Table 2, Supplementary Data 4)
Linear dynamic range (LDR)	E	The linear dynamic range (LDR) included the used 1:10 cDNA dilution in all cases and ranged from 3x $\log_{10}$ (cDNA stock dilution 1:10 – 1:10^3) to 6x $\log_{10}$ (cDNA stock dilution 1:10 – 1:10^6) for the individual genes (primer pairs), see Supplementary Data 4. Standard curves were calculated only considering dilutions within the LDR. (Supplementary Data 4)
C_q variation at LOD	E	**GAPDH:** SD=0.952; **PPIB:** SD=1.77; **YWHAZ:** SD=1.696; **POLR2A:** SD=1.004; **TBP:** SD=0.561; **EEF1A1:** SD=0.405; **RPLP0:** SD=0.176; **RNA18S5:** SD=0.000; **RPL22:** SD=0.202. (Supplementary Data 4)
CIs throughout range	D	CIs of C_q were calculated throughout the dilution range for all genes tested and are given in Supplementary Data 4.
Evidence for LOD	E	Not detectable C_q value for ≥ 1 of the technical replicates (triplet) at the corresponding cDNA dilution level indicates LOD at the previous, more concentrated dilution level. LOD for all genes (primer pairs) detected at a cDNA quantity equivalent to ≤1 pg RNA, except for TBP with an LOD of 100 pg RNA equivalent (weak signal at 10 pg and 1 pg). (Supplementary Data 4)
If multiplex, efficiency and LOD of each assay	E	Not applicable.
**Data analysis**		
qPCR analysis program (source, version)	E	Mastercycler ep realplex software, version 2.2 (Eppendorf AG, Hamburg, Germany)
Method of C_q determination	E	Second derivative maximum method (CalqPlex algorithm, Automatic Baseline, Drift Correction On)
Outlier identification and disposition	E	For analysis none of the C_q values was discarded.
Results for NTCs	E	The signal of the amplification plot was very late and there was a high C_q value difference between the negative control and all cDNA samples. **GAPDH:** 33; **PPIB:** 35; **YWHAZ:** 36; **POLR2A:** 36; **TBP:** none; **EEF1A1:** 35; **RPLP0:** none; **RNA18S5:** 35; **RPL22:** none.

Justification of number and choice of reference genes	E	Aim of this study - identification of optimal number and choice of reference genes for hPDL fibroblasts under physiological conditions, in a model for orthodontic tooth movement and a model for bacterial periodontitis.
Description of normalization method	E	Samples were not normalized, since apart from the reference genes no target genes were quantified.
Number and concordance of biological replicates	D	N = 1 (pool of hPDL fibroblasts from 4 different patients); n = 6 (pool cells seeded in 6 different wells per experimental group as biological replicates).
Number and stage (RT or qPCR) of technical replicates	E	qPCR reactions were performed in triplets (technical replicates n = 3).
Repeatability (intraassay variation)	E	The maximum SD (of the mean) across all biological replicates (n=18) of the means of C_q from the three technical replicates was ≤0.553 in all instances.<br>**GAPDH:** 0.24; **PPIB:** 0.29; **YWHAZ:** 0.32; **POLR2A:** 0.35; **TBP:** 0.27; **EEF1A1:** 0.53; **RPLP0:** 0.36; **RNA18S5:** 0.20; **RPL22:** 0.33. (Table 2)
Reproducibility (interassay variation, CV)	D	High biological reproducibility was achieved as evidenced by the low SD of raw C_q values for all genes and experimental groups tested (see Figure 2, Supplementary Table 3).
Power analysis	D	The number of biological replicates (n = 6) was based on previous studies and corresponds to the number of replicates generally used in cell culture RT-qPCR experiments.
Statistical methods for results significance	E	All biological samples (n = 6) were measured in triplicate (n = 3) and an arithmetic mean of each C_q triplett used for further analysis. The stability of each candidate was calculated with four different mathematical algorithms: geNorm, NormFinder, BestKeeper and the comparative ΔC_q method. Stability calculations were done with the official Microsoft-Excel-based software applets for geNorm, NormFinder and BestKeeper according to developers' instructions. For the comparative ΔC_q method manual calculations were performed. The geNorm and NormFinder algorithms require the transformation of the raw C_q data to linear scale expression quantities Q corresponding to the qPCR efficiency (E) of each gene: $Q = E^{-(Cqmin-Cqsample)}$ with the lowest C_q value corresponding to a quantity of 1 for each candidate reference gene. The genes were ranked according to their stability values (geNorm: M, NormFinder: ρ_{ig}/σ_i, deltaCT: mean SD of ΔC_q; BestKeeper: Pearson's r) for each algorithm and each experimental condition as well as combined experimental conditions (no treatment + compressive force, no treatment + Agac) and a rank sum of all algorithms calculated per gene for final stability assessment with the smallest rank sum indicating the most stable reference gene. Also a pooled overall ranking for all experimental conditions was calculated. The geNorm algorithm was used to calculate the ideal number of reference genes for reliable RT-qPCR normalization. If pairwise variation (V_n/V_{n+1}) between two sets of reference genes with one set including an additional reference gene was ≤0.15, this additional gene was deemed unnecessary for normalization. To assess ranking variations between the algorithms, we used IBM SPSS Statistics® 23 (IBM, Armonk, NY, USA) to create a correlation matrix of bivariate correlations (Pearson´s correlation coefficient r, normality confirmed by Shapiro-Wilk tests and histogram evaluation) of the overall pooled stability values as calculated by two respective algorithms. (see Figures 3 and 4, Table 3, Supplementary Table 4).
Software (source, version)	E	Microsoft Excel 2010 (Microsoft Corporation, Redmond, USA);<br>IBM SPSS Statistics® 23 (IBM, Armonk, NY, USA)

C_q or raw data submission	D	Raw C_q values are provided in Figure 2 and Supplementary Table 3.
**qPCR target information**		
Gene symbol	E	Provided in Table 1. We based our primer design on the officially registered target gene nucleotide sequences from the NCBI Nucleotide database (GeneBank, access: http://www.ncbi.nlm.nih.gov/nuccore).
Sequence accession number	E	
Location of amplicon	D	Provided in Table 1.
Amplicon length	E	Provided in Table 1. Target amplicon sequences were chosen to range from 60 to 150 bp with a GC content of 35–65%.
In silico specificity screen (BLAST, and so on)	E	Provided in Table 1. In-silico specify of constructed primers was ensured by PrimerBLAST (National Center for Biotechnology Information, Bethesda MD, USA, https://www.ncbi.nlm.nih.gov/tools/primer-blast; RefSeq mRNA, Splice variants allowed, Max. Product Size: 4000) and cross-checked using the UCSC in-silico-PCR Genome Browser (Dec. 2013 GRCh38/hg38; UCSC Genes; Max. Product Size: 4000; Min. Perfect Match: 15; Min. Good Match: 15; Jim Kent, http://genome-mirror.genomedk.au.dk/cgi-bin/hgPcr). Intron-flanking primer pairs were designed to prevent a co-amplification of genomic DNA and checked in silico for sufficient absence of hairpin structures and dimer formation at annealing temperature ($\Delta G \geq -3,5$ kcal/mol, BeaconDesigner™ Free Edition, Premier BioSoft International, Palo Alto, CA, USA, http://www.premierbiosoft.com/qOligo/Oligo.jsp?PID=1).
Pseudogenes, retropseudogenes or other homologs	D	Sequence alignment, possible splicing and targeted transcript variants as well as absence of targeted pseudogenes, retropseudogenes or other homologs were assessed upon primer construction by NCBI PrimerBLAST (National Center for Biotechnology Information, Bethesda MD, USA, https://www.ncbi.nlm.nih.gov/tools/primer-blast) and PrimerCheck (SpliceCenter der Genomics and Bioinformatics Group, LMP, CCR, NCI, http://projects.insilico.us/SpliceCenter/PrimerCheck.jsp).
Sequence alignment	D	
Secondary structure analysis of amplicon	D	Provided in Supplementary Data 1. No secondary structures present at annealing temperature (60°C) were detected as determined in silico by UNAFold (http://eu.idtdna.com/UNAFold?, Suboptimality 50%; Integrated DNA Technologies Inc., Coralville, IA, USA).
Location of each primer by exon or intron	E	Provided in Table 1. Also see Supplementary Data 1.
What splice variants are targeted	E	Provided in Table 1. Also see Supplementary Data 1.
**qPCR oligonucleotides**		
Primer sequences	E	Provided in Table 1.
RTPrimerDB identification number	D	Not applicable, primers were constructed and validated by the authors.
Probe sequences	D	Not applicable.
Location and identity of any modifications	E	Primers received no terminal or other modifications.
Manufacturer of oligonucleotides	D	Primers were synthesized by Eurofins MWG Operon LLC (Huntsville, AL, USA).
Purification method	D	Primers were purified by High Purity Salt Free Purification HPSF® (Eurofins MWG Operon LLC).

Supplementary Table 2. Yield (quantity) and quality of extracted total RNA per biological replicate (well).

Sample ID	Nucleic Acid Concentration	Unit	A260	A280	A260/A280	Sample Type	Factor
Control K7	450.8	ng/µl	11.269	5.757	1.96	RNA	40
Control K8	495.4	ng/µl	12.385	6.415	1.93	RNA	40
Control K9	488.2	ng/µl	12.206	6.494	1.88	RNA	40
Control K10	486.2	ng/µl	12.156	6.407	1.9	RNA	40
Control K11	444.4	ng/µl	11.11	5.793	1.92	RNA	40
Control K12	472.8	ng/µl	11.82	6.125	1.93	RNA	40
Compression D7	295.1	ng/µl	7.377	3.922	1.88	RNA	40
Compression D8	291.6	ng/µl	7.29	3.826	1.91	RNA	40
Compression D9	225.2	ng/µl	5.631	3.002	1.88	RNA	40
Compression D10	225.9	ng/µl	5.647	3.098	1.82	RNA	40
Compression D11	218.6	ng/µl	5.464	2.934	1.86	RNA	40
Compression D12	298.9	ng/µl	7.473	3.952	1.89	RNA	40
Agac7	441	ng/µl	11.026	5.873	1.88	RNA	40
Agac8	245.5	ng/µl	6.138	3.262	1.88	RNA	40
Agac9	303	ng/µl	7.575	3.935	1.93	RNA	40
Agac10	456.7	ng/µl	11.417	6.052	1.89	RNA	40
Agac11	295.2	ng/µl	7.38	3.902	1.89	RNA	40
Agac12	312.6	ng/µl	7.814	4.149	1.88	RNA	40

A = absorbance = optical density (OD) at 260nm and 280nm; A260/A280 = absorbance ratio.

Factor = ng/µl total RNA per 1 unit OD_{260nm}.

Supplementary Table 3. Raw C_q values of RT-qPCR (triplet means) for 3 experimental groups and 9 candidate reference genes.

**RAW C_q values**		Gene	Gene	Gene	Gene	Gene	Gene	Gene	Gene	Gene
**Sample**	**Group**	**GAPDH**	**PPIB**	**YWHAZ**	**POLR2A**	**TBP**	**RPL22**	**RPLP0**	**EEF1A1**	**RNA18S5**
Control K7	1	15.04	16.43	21.81	20.96	23.35	18.22	16.34	14.17	8.67
Control K8	1	15.14	16.40	21.73	21.03	23.29	18.13	16.18	14.14	8.42
Control K9	1	15.14	16.36	21.51	20.77	23.30	18.00	16.33	14.09	7.91
Control K10	1	15.26	16.51	21.60	20.83	23.47	18.21	16.24	14.12	8.33
Control K11	1	15.04	16.30	21.21	21.00	23.46	17.95	16.06	14.02	8.41
Control K12	1	15.09	16.36	21.00	20.60	23.12	17.93	16.22	14.06	7.81
Compression D7	2	15.13	16.98	22.57	21.78	24.08	18.39	16.37	14.26	8.97
Compression D8	2	14.85	16.75	22.14	21.50	23.72	18.19	16.26	13.91	8.57
Compression D9	2	14.70	16.80	21.74	21.66	23.75	18.26	16.42	13.97	8.52
Compression D10	2	15.05	16.74	21.11	21.45	23.50	18.20	16.46	13.97	8.08
Compression D11	2	15.11	16.52	21.01	21.51	23.64	17.95	16.26	13.75	8.27
Compression D12	2	14.85	16.71	21.28	21.57	23.67	18.21	16.06	13.85	8.05
Agac7	3	15.58	16.43	21.84	21.07	23.32	17.82	16.19	14.15	8.76
Agac8	3	15.41	16.63	21.46	21.45	23.67	18.36	16.55	14.44	8.46
Agac9	3	15.27	16.48	20.97	21.18	23.46	18.21	16.42	14.41	7.99
Agac10	3	15.37	16.48	21.03	21.15	23.43	18.36	16.43	14.37	8.02
Agac11	3	15.58	16.71	21.04	21.36	23.69	18.41	16.83	14.57	8.32
Agac12	3	15.40	16.66	20.89	21.09	23.50	18.35	16.65	14.46	7.91
**C_q SD Control**	1	0.08	0.07	0.31	0.16	0.13	0.13	0.10	0.05	0.33
**C_q SD Compression**	2	0.17	0.15	0.62	0.12	0.19	0.14	0.15	0.17	0.35
**C_q SD Agac**	3	0.12	0.12	0.37	0.15	0.14	0.22	0.22	0.14	0.33

C_q = quantification cycle; SD = standard deviation of group mean. Gene symbols see Table 1. Agac = Aggregatibacter actinomycetemcomitans (periodontitis)

Supplementary Table 4. Gene stability ranking for individual experimental groups of the nine analysed candidate reference genes according to their expression stability as calculated by the algorithms geNorm, NormFinder, comparative ΔC_q and BestKeeper.

Rank	Total (of 4 methods)		geNorm		NormFinder			comparative deltaC$_q$		BestKeeper			
	Ranking order	Rank sum	Ranking order	Stability value (M)	Ranking order	Stability value (ρ_{ig}/σ_i)	Standard error	Ranking order	Stability value (mean SD of mean ΔC_q)	Ranking order	Stability value (r)	SD (+/- C_q)	CV (% C_q)
Untreated control (physiological conditions)													
1.)	**RPL22**	**7**	**EEF1A1**	**0.138**	**RPL22**	**0.005**	**0.082**	**EEF1A1**	**0.146**	**RNA18S5**	**0.915**	**0.266**	**3.216**
2.)	**EEF1A1**	**9**	**PPIB**	**0.142**	**EEF1A1**	**0.045**	**0.024**	**RPL22**	**0.147**	**RPL22**	**0.903**	**0.113**	**0.627**
3.)	PPIB	14	**RPL22**	**0.142**	PPIB	0.055	0.025	PPIB	0.147	YWHAZ	0.890	0.248	1.154
4.)	TBP	20	GAPDH	0.164	TBP	0.070	0.028	GAPDH	0.173	POLR2A	0.771	0.132	0.631
5.)	GAPDH	23	TBP	0.165	POLR2A	0.073	0.029	TBP	0.177	**EEF1A1**	**0.735**	**0.043**	**0.307**
6.)	POLR2A	23	RPLP0	0.175	GAPDH	0.086	0.032	RPLP0	0.182	TBP	0.621	0.095	0.407
7.)	RPLP0	27	POLR2A	0.178	RPLP0	0.091	0.033	POLR2A	0.188	PPIB	0.579	0.053	0.325
8.)	YWHAZ	27	YWHAZ	0.248	YWHAZ	0.148	0.049	YWHAZ	0.266	RPLP0	0.186	0.075	0.462
9.)	RNA18S5	28	RNA18S5	0.290	RNA18S5	0.187	0.061	RNA18S5	0.290	GAPDH	0.127	0.062	0.408
Compressive orthodontic force (model for orthodontic tooth movement)													
1.)	**EEF1A1**	**9**	**PPIB**	**0.177**	**EEF1A1**	**0.012**	**0.072**	**PPIB**	**0.185**	**RNA18S5**	**0.958**	**0.277**	**3.290**
2.)	**PPIB**	**10**	**EEF1A1**	**0.181**	**TBP**	**0.021**	**0.045**	**EEF1A1**	**0.187**	**RPL22**	**0.938**	**0.508**	**2.349**
3.)	TBP	15	POLR2A	0.190	**PPIB**	**0.036**	**0.034**	RPL22	0.200	YWHAZ	0.913	0.126	0.529
4.)	POLR2A	17	RPL22	0.190	POLR2A	0.065	0.033	POLR2A	0.201	POLR2A	0.905	0.115	0.824
5.)	RPL22	18	TBP	0.194	RPL22	0.067	0.034	TBP	0.203	**EEF1A1**	**0.872**	**0.093**	**0.557**
6.)	RNA18S5	23	RPLP0	0.246	RPLP0	0.131	0.047	RPLP0	0.258	TBP	0.804	0.094	0.438
7.)	RPLP0	26	GAPDH	0.279	RNA18S5	0.140	0.050	RNA18S5	0.286	PPIB	0.752	0.087	0.476
8.)	YWHAZ	29	RNA18S5	0.283	GAPDH	0.166	0.057	GAPDH	0.296	RPLP0	0.390	0.112	0.685
9.)	GAPDH	32	YWHAZ	0.474	YWHAZ	0.324	0.103	YWHAZ	0.515	GAPDH	0.177	0.148	0.992
Bacterial lysate of Aggregatibacter actinomycetemcomitans (Agac, model for bacterial periodontitis)													
1.)	**TBP**	**7**	**TBP**	**0.175**	**TBP**	**0.035**	**0.035**	**TBP**	**0.184**	**POLR2A**	**0.742**	**0.126**	**0.592**
2.)	**POLR2A**	**10**	**PPIB**	**0.180**	**POLR2A**	**0.038**	**0.034**	**PPIB**	**0.187**	**GAPDH**	**0.715**	**0.097**	**0.626**
3.)	PPIB	11	**POLR2A**	**0.192**	PPIB	0.038	0.033	EEF1A1	0.199	RNA18S5	0.691	0.270	3.275
4.)	GAPDH	16	EEF1A1	0.194	GAPDH	0.055	0.032	**POLR2A**	**0.203**	TBP	0.650	0.112	0.477
5.)	EEF1A1	20	GAPDH	0.218	EEF1A1	0.090	0.037	GAPDH	0.227	PPIB	0.537	0.102	0.614
6.)	RPLP0	25	RPLP0	0.223	RPLP0	0.124	0.045	RPLP0	0.236	YWHAZ	0.463	0.297	1.399
7.)	RNA18S5	27	RPL22	0.249	RPL22	0.155	0.054	RPL22	0.260	RPLP0	0.379	0.165	0.999
8.)	RPL22	30	RNA18S5	0.346	RNA18S5	0.224	0.073	RNA18S5	0.354	EEF1A1	0.222	0.093	0.648
9.)	YWHAZ	33	YWHAZ	0.386	YWHAZ	0.255	0.083	YWHAZ	0.410	RPL22	0.078	0.158	0.864

C_q = quantification cycle; SD = standard deviation; CV = coefficient of variation; r = Pearson's correlation coefficient.

Supplementary Table 5. Marker genes, primers and amplicons used for characterization of hPDL fibroblasts.

**Gene symbol**	**Gene name** (Homo sapiens)	**Accession Number** (NCBI GenBank)	**Chromosomal location** (length)	**5´-forward primer-3´** (length / T_m / %GC / max. ΔG Hairpin &Self-Dimer / Self-Comp. / Self-3'-Comp.)	**5´-reverse primer-3´** (length / T_m / %GC / max. ΔG Hairpin &Self-Dimer / Self-Comp. / Self-3'-Comp.)	**Primer Location** (max. ΔG Cross-Dimer)	**Amplicon** (length, %GC, T_m, SSAT)	**Amplicon location** (bp of Start/Stop)	**Intron -flanking** (length)	**In silico qPCR specifity**	**Variants targeted (Transcript /Splice)**
**VIM**	vimentin	NM_003380.3	10p13 (2151bp)	CTGGATTCACTCCCTCTGGTTG (22bp / 62.1°C / 54.5% / -1.3 / 5 / 0)	CGTGATGCTGAGAAGTTTCGTTG (23bp / 60.6°C / 47.8% / -0.6 / 4 / 0)	exon 8/9 (-2.6)	106bp, 44.3%, 82.3%, no SSAT	1695/1800	Yes (850bp)	Yes (BLAST/ UCSC)	Yes
**P4HA1**	prolyl 4-hydroxylase, alpha polypeptide I	NM_000917.3	10q22.1 (2860bp)	GCTCTCTGGCTATGAAAATCCTG (23bp / 60.6°C / 47.8% / 0.0 / 2 / 2)	GTGCAAAGTCAAAATGGGGTTC (22bp / 58.4°C / 45.5% / -3.4 / 4 / 0)	exon 13/14 (-0.9)	146bp, 41.1%, 82.2°C, no SSAT	1396/1541	Yes (13371bp)	Yes (BLAST/ UCSC)	Yes
**FN1**	fibronectin 1	NM_212482.1	2q34 (8815bp)	GCCAGTCCTACAACCAGTATTCTC (24bp / 62.7°C / 50.0% / -0.3 / 4 / 2)	GCTTGTTCCTCTGGATTGGAAAG (23bp / 60.6°C / 47.8% / -2.5 / 4 / 1)	exon 45/46 (-3.0)	150bp, 42.7%, 83.1°C, no SSAT	7579/7728	Yes (342bp)	Yes (BLAST/ UCSC)	Yes
**COL1A2**	collagen, type I, alpha 2	NM_000089.3	7q22.1 (5411bp)	AGAAACACGTCTGGCTAGGAG (21bp / 59.8°C / 52.4% / -3.3 / 4 / 2)	GCATGAAGGCAAGTTGGGTAG (21bp / 59.8°C / 52.4% / -2.3 / 5 / 0)	exon 50/51 (-0.7)	105bp, 44.8%, 83.3°C no SSAT	4139/4243	Yes (710bp)	Yes (BLAST/ UCSC)	Yes
**FMOD**	fibromodulin	NM_002023.4	1q32 (3271bp)	AGTCAACACCAACCTGGAGAAC (22bp / 60.3°C / 50.0% / -1.5 / 3 / 0)	GAAGTTCACGACGTCCACCAC (21bp / 61.8°C / 57.1% / -6.5 / 6 / 3)	exon 2/3 (-2.8)	97bp, 51.6%, 85.7°C no SSAT	1334/1430	Yes (4797bp)	Yes (BLAST/ UCSC)	Yes
**TNFRSF11B (OPG)**	tumor necrosis factor receptor superfamily, member 11b (osteoprotegerin)	NM_002546.3	8q24 (2354bp)	TGTCTTTGGTCTCCTGCTAACTC (23bp / 60.6°C / 47.8% / 0.0 / 2 / 0)	CCTGAAGAATGCCTCCTCACAC (22bp / 62.1°C / 54.5% / -0.9 / 4 / 0)	exon 3/4 (-1.8)	124bp, 42.7%, 83.1°C no SSAT	824/947	Yes (2019bp)	Yes (BLAST/ UCSC)	Yes
**POSTN**	periostin	NM_006475.2	13q13.3 (3390bp)	AGACACACCCGTGAGGAAG (19bp / 58.8°C / 57.9% / -1.3 / 4 / 0)	GGTCAGGTTATTGACTTAGGGTTG (24bp / 61.0°C / 45.8% / -2.6 / 4 / 0)	exon 23/24 (-3.4)	136bp, 39.4%, 81.9°C no SSAT	2548/2683	Yes (1148bp)	Yes (BLAST/ UCSC)	Yes
**RUNX2**	runt related transcription factor 2	NM_001024630.3	6p21 (5553bp)	CAGTAGATGGACCTCGGGAAC (21bp / 61.8°C / 57.1% / 0.0 / 3 / 0)	TGAGGCGGTCAGAGAACAAAC (21bp / 59.8°C / 52.4% / -0.9 / 3 / 0)	exon 5/6 (-3.1)	81bp, 50.6%, 83.7°C no SSAT	869/949	Yes (53889bp)	Yes (BLAST/ UCSC)	Yes
**SMAD1**	SMAD family member 1	NM_005900.2	4q31 (3056bp)	AGCAGCACCTACCCTCACTC (20bp / 61.4°C / 60.0% / 0.0 / 3 / 0)	CTTCAGGAGGCAGGTAAGCAG (21bp / 61.8°C / 57.1% / -0.5 / 3 / 1)	exon 4/5 (-2.9)	97bp, 60.8%, 90.4°C, no SSAT	1014/1110	Yes (2520bp)	Yes (BLAST/ UCSC)	Yes
**ALPL**	alkaline phosphatase, liver/bone/kidney	NM_000478.4	1p36.12 (2606bp)	ACAAGCACTCCCACTTCATCTG (22bp / 60.3°C / 50.0% / -0.5 / 3 / 2)	GGTCCGTCACGTTGTTCCTG (20bp / 61.4°C / 60.0% / -3.3 / 5 / 1)	exon 7-8/9 (-2.1)	132bp, 56.1%, 89.5°C, no SSAT	1045/1176	Yes (3290bp)	Yes (BLAST/ UCSC)	Yes
**SCX**	scleraxis bHLH transcription factor	NM_001080514.2	8q24.3 (1027bp)	CCAGCCCAAACAGATCTGCAC (21bp / 61.8°C / 57.1% / -7.9 / 8 / 2)	TGCGAATCGCTGTCTTTCTGTC (22bp / 60.3°C / 50.0% / -4.2 / 7 / 1)	exon 1/2 (-3.8)	83bp, 54.2%, 86.6°C, no SSAT	575/657	Yes (923bp)	Yes (BLAST/ UCSC)	Yes
**S100A4**	S100 calcium binding protein A4	NM_002961.2	1q21 (512bp)	TCTCTACAACCCTCTCTCCTCAG (23bp / 62.4°C / 52.2% / 0.0 / 3 / 3)	GGAAGGTGGACACCATCACATC (22bp / 62.1°C / 54.5% / -3.2 / 8 / 1)	exon 1/3 (-1.5)	108bp, 54.1%, 87.8°C no SSAT	11/118	Yes (943bp)	Yes (BLAST/ UCSC)	Yes
**NCAM1**	neural cell adhesion molecule 1 (NCAM1)	NM_000615.6	11q23.1 (5977bp)	CTCCCACCAACCATCATCTGG (21bp / 61.8°C / 57.1% / -1.5 / 3 / 2)	CAGGATTCTGCCCTCACAGC (20bp / 61.4°C / 60.0% / -1.3 / 6 / 2)	exon 4/5 (-1.3)	150bp, 49.3%, 86.6°C no SSAT	799/948	Yes (376bp)	Yes (BLAST/ UCSC)	Yes

T_m = melting temperature of primer/specific qPCR product (amplicon); %GC = guanine/cytosine content; bp = base pairs; Comp. = Complementarity; SSAT = secondary structure at annealing temperature.

Supplementary Figure 1. Characterization of human PDL fibroblasts. (**a)** Cell morphology of isolated hPDL cells. All cells show a spindle-shaped cell morphology. **(b)** Specific gene expression profile of hPDL markers (western blot of PCR products): untreated control samples of individual patients and of final hPDL cell pool (experimental groups). Abbreviations see Supplementary Table 5.

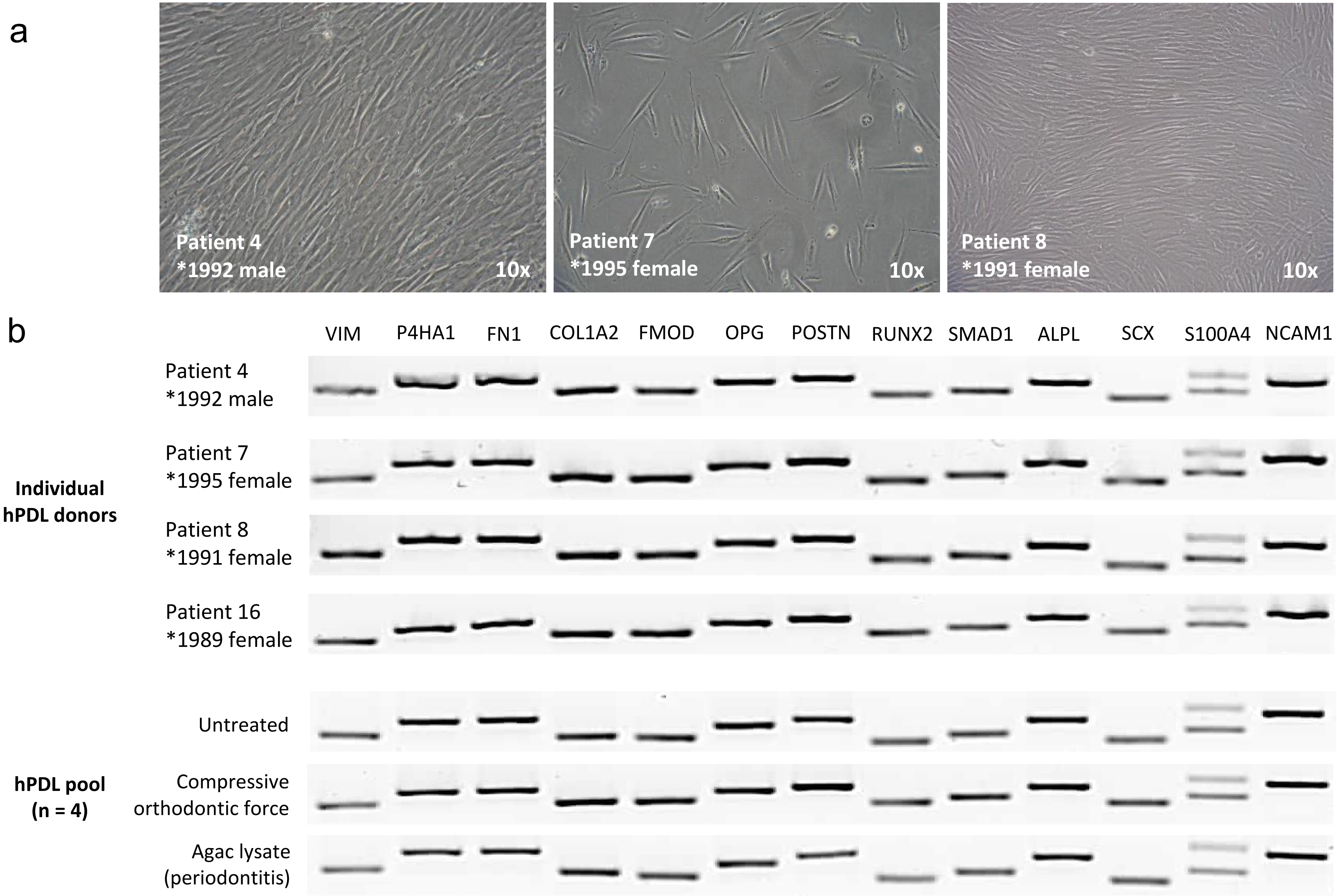

Supplementary Figure 2. Uncropped original gel of RT-qPCR products (amplification specifity). For each candidate reference gene / primer pair we found a single fluorescent band at the expected amplicon size. bp = base pairs. Gene names see Table 1. All RT-qPCR products were run concurrently and adjacently on the same gel, which was recorded with the gel documentation system Genoplex 2 (VWR International GmbH, Darmstadt, Germany) and its software GenoCapture (version 7.01, Synoptics Ltd., Cambridge, UK - automatic exposure, exposure time 80 ms, no binning, transillumination) as secure gel data (*.sgd) and exported as TIF image, which was inverted and cropped to encompass the relevant gel area.

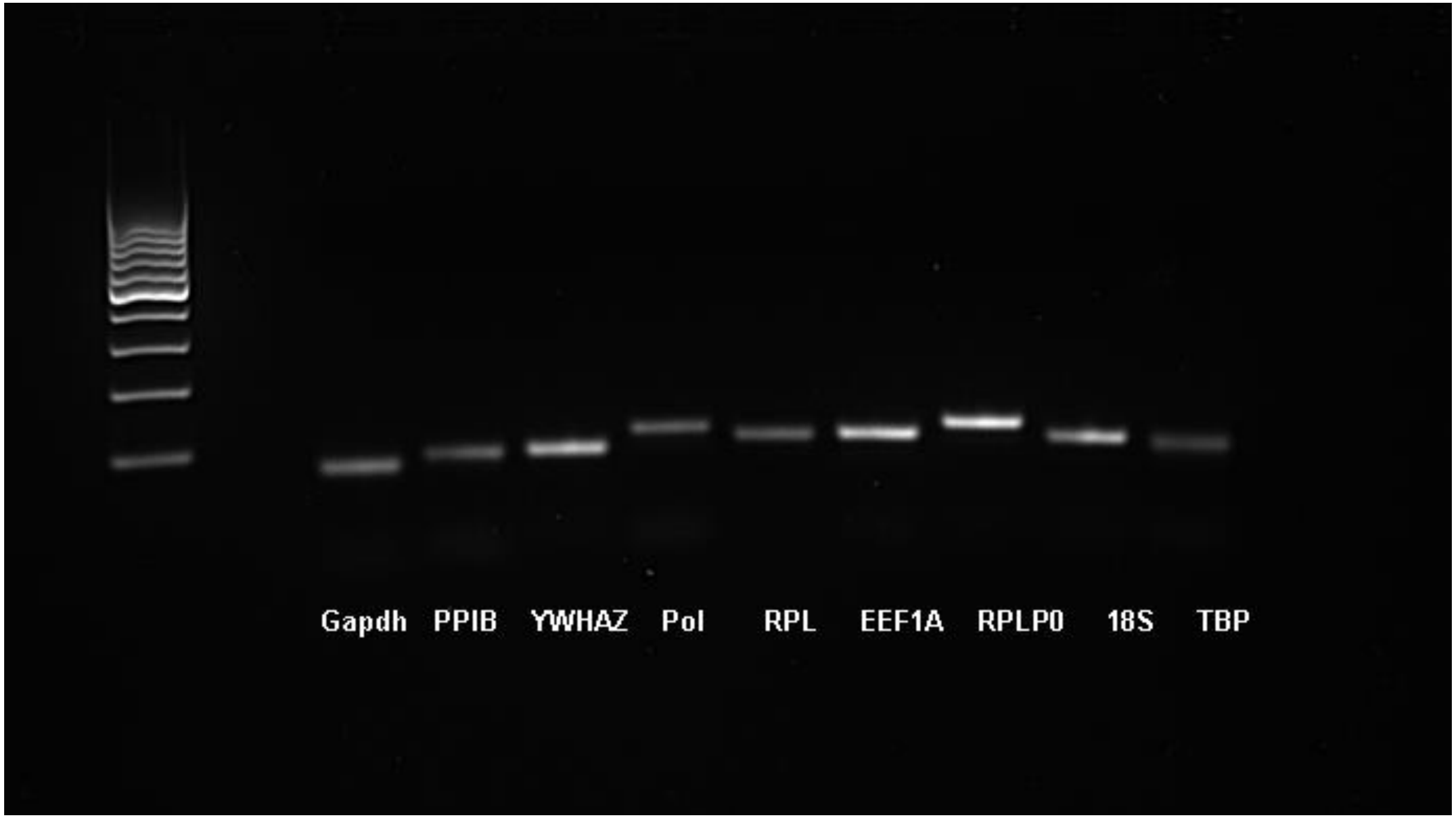

Supplementary Data 1. Splice variants and secondary structure analysis of amplicons and primers of the nine evaluated candidate reference genes.

GAPDH PrimerBLAST (National Center for Biotechnology Information, Bethesda MD, USA, https://www.ncbi.nlm.nih.gov/tools/primer-blast)

Primer pair 1

	Sequence (5'->3')	Template strand	Length	Start	Stop	Tm	GC%	Self complementarity	Self 3' complementarity
**Forward primer**	TGCCCTCAACGACCACTTTG	Plus	20	1091	1110	63.28	55.00	3.00	2.00
**Reverse primer**	CCACCACCCTGTTGCTGTAG	Minus	20	1164	1145	63.08	60.00	4.00	2.00
**Product length**	74								
**Total intron size**	104 (between pos. 6527996 and 6528101 on NT_009759.17)								

Products on intended target

>NM_002046.5 Homo sapiens glyceraldehyde-3-phosphate dehydrogenase (GAPDH), transcript variant 1, mRNA

```
product length = 74
Forward primer  1     TGCCCTCAACGACCACTTTG  20
Template        1091  ....................  1110

Reverse primer  1     CCACCACCCTGTTGCTGTAG  20
Template        1164  ....................  1145
```

Products on allowed transcript variants

>NM_001289746.1 Homo sapiens glyceraldehyde-3-phosphate dehydrogenase (GAPDH), transcript variant 4, mRNA

```
product length = 74
Forward primer  1     TGCCCTCAACGACCACTTTG  20
Template        1077  ....................  1096

Reverse primer  1     CCACCACCCTGTTGCTGTAG  20
Template        1150  ....................  1131
```

>NM_001289745.1 Homo sapiens glyceraldehyde-3-phosphate dehydrogenase (GAPDH), transcript variant 3, mRNA

```
product length = 74
Forward primer  1     TGCCCTCAACGACCACTTTG  20
Template        1183  ....................  1202

Reverse primer  1     CCACCACCCTGTTGCTGTAG  20
Template        1256  ....................  1237
```

>NM_001256799.2 Homo sapiens glyceraldehyde-3-phosphate dehydrogenase (GAPDH), transcript variant 2, mRNA

```
product length = 74
Forward primer  1     TGCCCTCAACGACCACTTTG  20
Template        1125  ....................  1144

Reverse primer  1     CCACCACCCTGTTGCTGTAG  20
Template        1198  ....................  1179
```

GAPDH PrimerCheck (SpliceCenter der Genomics and Bioinformatics Group, LMP, CCR, NCI, http://projects.insilico.us/SpliceCenter/PrimerCheck.jsp)

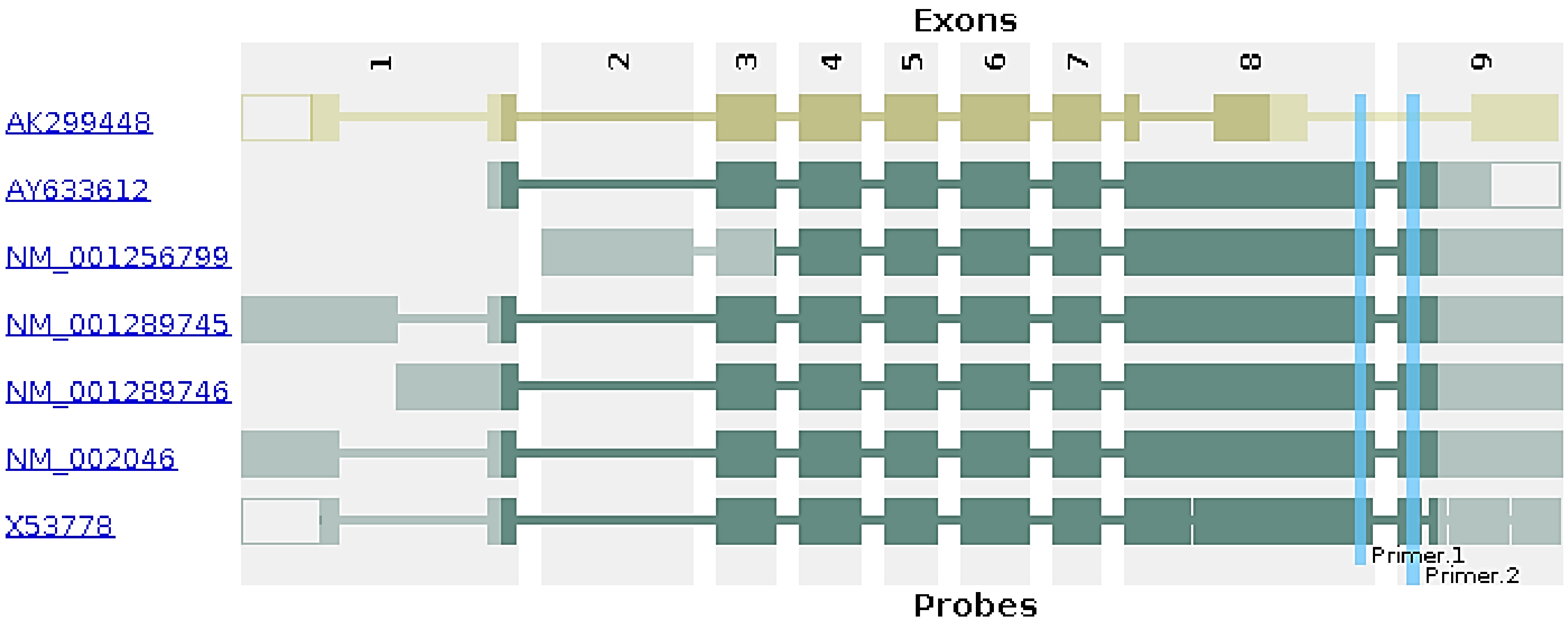

GAPDH

GAPDH UNAFold (Integrated DNA Technologies Inc., Coralville, IA, USA, http://eu.idtdna.com/UNAFold? , Suboptimality 50%)

GAPDH Amplicon Sequence

5’ TGCCCTCAACGACCACTTTGTCAAGCTCATTTCCTGGTATGACAACGAATTTGGCTACAGCAACAGGGTGGTGG 3’

Structures

Structure Name	Image	ΔG (kcal.mole^{-1})	T_M (^oC)	ΔH (kcal.mole^{-1})	ΔS (cal.K^{-1}mole^{-1})	Output
1	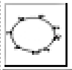	1.04	47	-25.6	-79.96	Ct Det
2	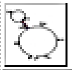	1.33	49.9	-42.7	-132.17	Ct Det
3	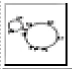	1.49	41.3	-25	-79.51	Ct Det
4	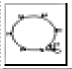	1.58	47.1	-39.2	-122.4	Ct Det
5	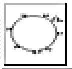	1.66	41.2	-27.8	-88.44	Ct Det
6	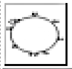	1.82	-30.4	-4.9	-20.19	Ct Det
7	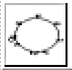	1.92	-28.6	-5.3	-21.67	Ct Det
8	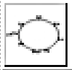	1.98	34.3	-23.6	-76.77	Ct Det

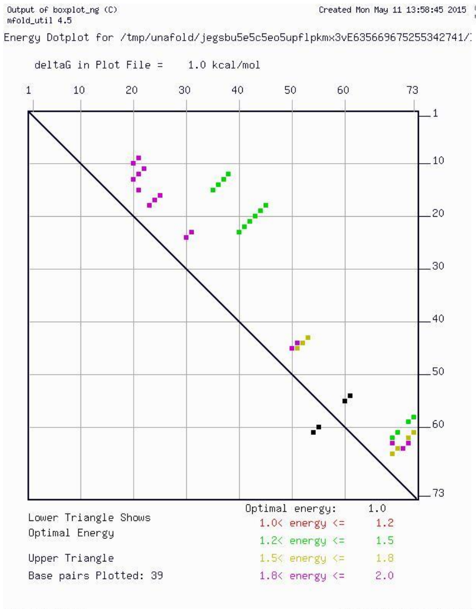

Output of boxplot_ng (C)
mfold_util 4.5
Created Mon May 11 13:58:45 2015
Energy Dotplot for /tmp/unafold/jegsbu5e5c5eo5upflpkmx3vE635669675255342741/
deltaG in Plot File = 1.0 kcal/mol
Lower Triangle Shows Optimal Energy
Upper Triangle Base pairs Plotted: 39
Optimal energy: 1.0
1.0< energy <= 1.2
1.2< energy <= 1.5
1.5< energy <= 1.8
1.8< energy <= 2.0

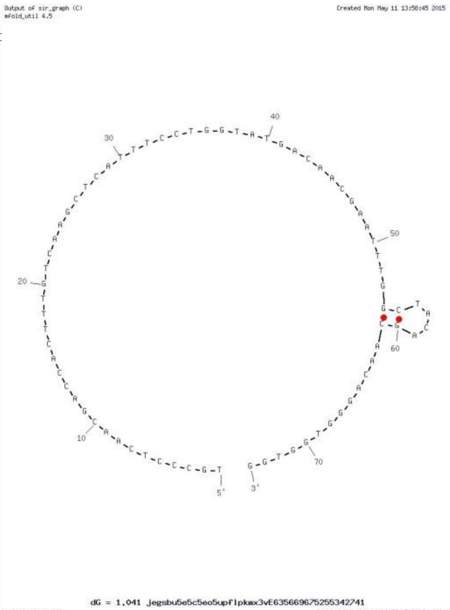

Output of sir_graph (C)
mfold_util 4.5
Created Mon May 11 13:58:45 2015
dG = 1.041 jegsbu5e5c5eo5upflpkmx3vE635669675255342741

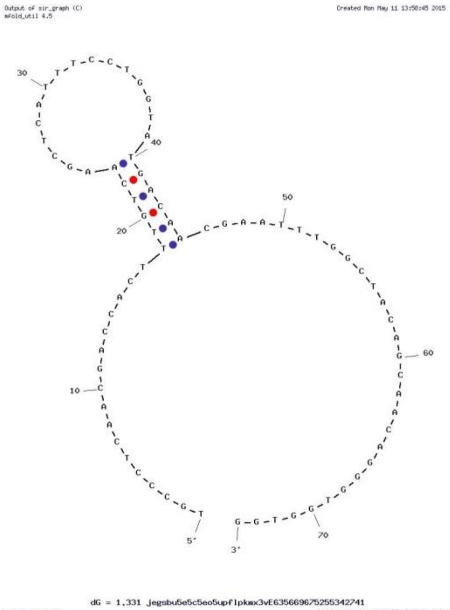

Output of sir_graph (C)
mfold_util 4.5
Created Mon May 11 13:58:45 2015
dG = 1.331 jegsbu5e5c5eo5upflpkmx3vE635669675255342741

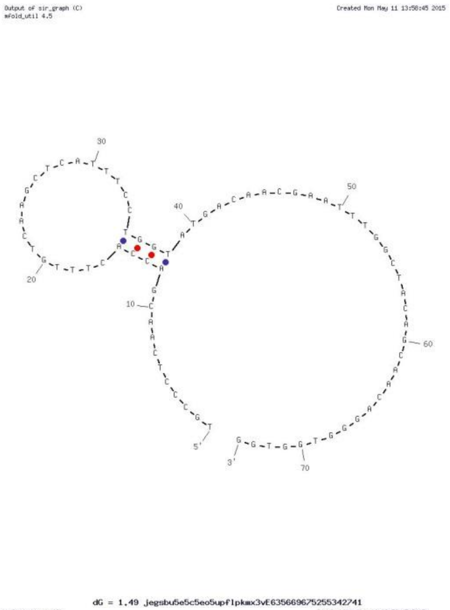

Output of sir_graph (C)
mfold_util 4.5
Created Mon May 11 13:58:45 2015
dG = 1.49 jegsbu5e5c5eo5upflpkmx3vE635669675255342741

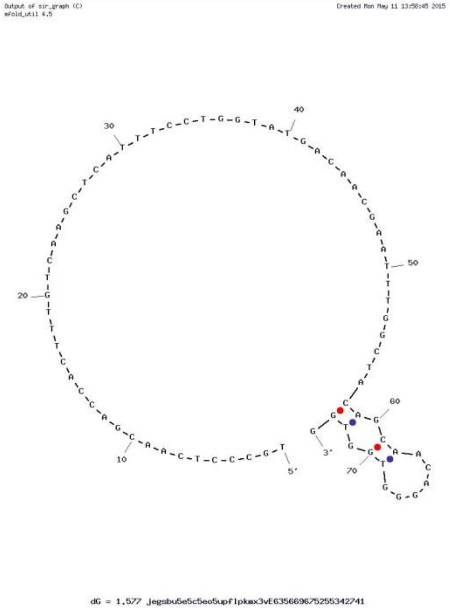

Output of sir_graph (C)
mfold_util 4.5
Created Mon May 11 13:58:45 2015
dG = 1.577 jegsbu5e5c5eo5upflpkmx3vE635669675255342741

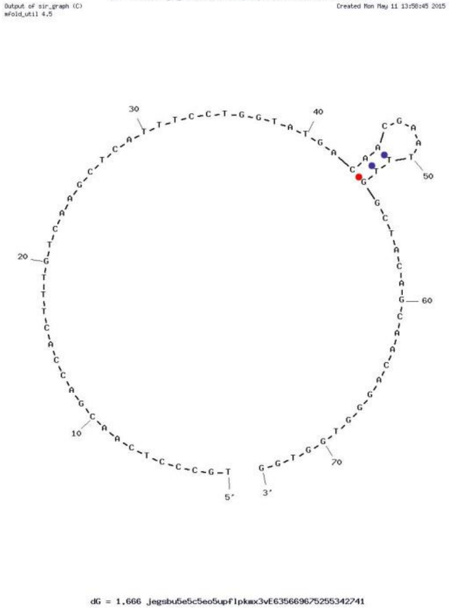

Output of sir_graph (C)
mfold_util 4.5
Created Mon May 11 13:58:45 2015
dG = 1.666 jegsbu5e5c5eo5upflpkmx3vE635669675255342741

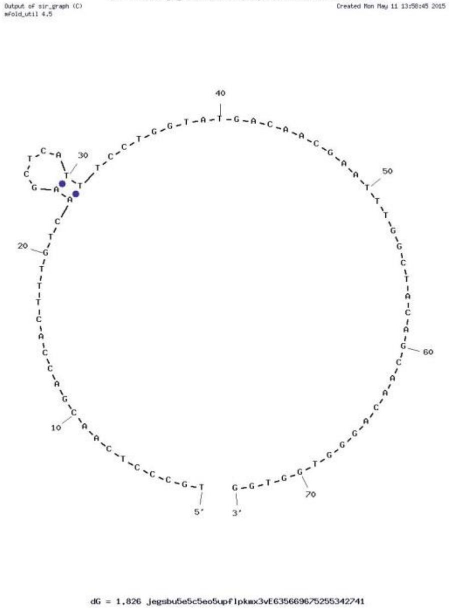

Output of sir_graph (C)
mfold_util 4.5
Created Mon May 11 13:58:45 2015
dG = 1.826 jegsbu5e5c5eo5upflpkmx3vE635669675255342741

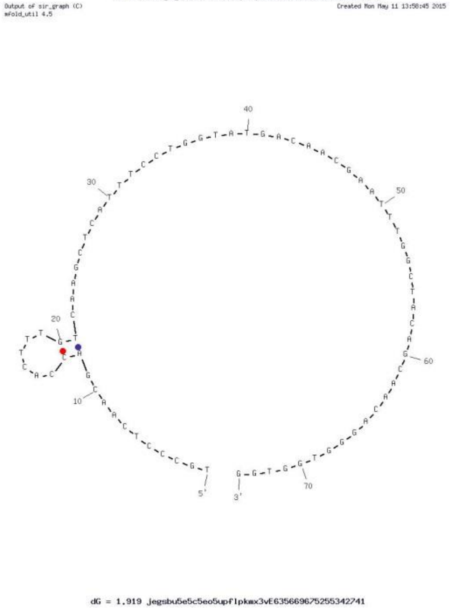

Output of sir_graph (C)
mfold_util 4.5
Created Mon May 11 13:58:45 2015
dG = 1.919 jegsbu5e5c5eo5upflpkmx3vE635669675255342741

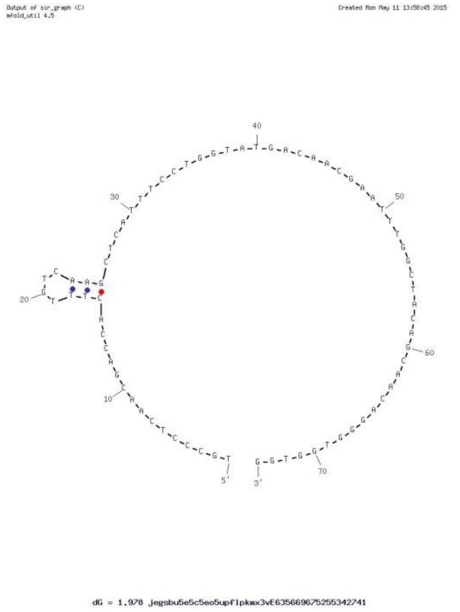

GAPDH UCSC In-silico-PCR Genome Browser (Dec. 2013 GRCh38/hg38; UCSC Genes; Max. Product Size: 4000; Min. Perfect Match: 15; Min. Good Match: 15; Jim Kent, http://genome-mirror.genomedk.au.dk/cgi-bin/hgPcr)

UCSC In-Silico PCR

The sequences and coordinates shown below are from UCSC Genes, not from the genome assembly. The links lead to the Genome Browser at the position of the entire target sequence.

```
>uc001qop.3__GAPDH:1091+1164 74bp TGCCCTCAACGACCACTTTG CCACCACCCTGTTGCTGTAG
TGCCCTCAACGACCACTTTGtcaagctcatttcctggtatgacaacgaat
ttggCTACAGCAACAGGGTGGTGG

>uc031qfw.2__GAPDH:1125+1198 74bp TGCCCTCAACGACCACTTTG CCACCACCCTGTTGCTGTAG
TGCCCTCAACGACCACTTTGtcaagctcatttcctggtatgacaacgaat
ttggCTACAGCAACAGGGTGGTGG

>uc031yrl.1__GAPDH:1183+1256 74bp TGCCCTCAACGACCACTTTG CCACCACCCTGTTGCTGTAG
TGCCCTCAACGACCACTTTGtcaagctcatttcctggtatgacaacgaat
ttggCTACAGCAACAGGGTGGTGG

>uc031yrm.1__GAPDH:1077+1150 74bp TGCCCTCAACGACCACTTTG CCACCACCCTGTTGCTGTAG
TGCCCTCAACGACCACTTTGtcaagctcatttcctggtatgacaacgaat
ttggCTACAGCAACAGGGTGGTGG
```

GAPDH BeaconDesigner™ Free Edition (Premier BioSoft International, Palo Alto, CA, USA, http://www.premierbiosoft.com/qOligo/Oligo.jsp?PID=1)

Secondary Structures for Sense Primer

Dimer:-

```
5' TGCCCTCAACGACCACTTTG 3'
         ||| ¦    ¦ ¦¦¦
      3' GTTTCACCAGCAACTCCCGT 5'
```

-0.7

Hairpin:-

```
/CAGCAACTCCCGT 5'
|  ¦ |||
\CACTTTG 3'
```

-0.7

Secondary Structures for Anti-sense Primer

Dimer:-

Not Found

Hairpin:-

Not Found

Cross Dimer

Cross Dimer between Sense Primer and Anti-sense Primer:-

```
5' TGCCCTCAACGACCACTTTG 3'
      ¦  ||||       ¦ ¦
3' GATGTCGTTGTCCCACCACC 5'
```

-2.4

PPIB PrimerBLAST (National Center for Biotechnology Information, Bethesda MD, USA, https://www.ncbi.nlm.nih.gov/tools/primer-blast)

Primer pair 1

	Sequence (5'->3')	Template strand	Length	Start	Stop	Tm	GC%	Self complementarity	Self 3' complementarity
**Forward primer**	TTCCATCGTGTAATCAAGGACTTC	Plus	24	446	469	61.33	41.67	4.00	2.00
**Reverse primer**	GCTCACCGTAGATGCTCTTTC	Minus	21	533	513	61.22	52.38	4.00	0.00
**Product length**	88								
**Total intron size**	3194 (between pos. 40883230 and 40880035 on NT_010194.18)								

Products on intended target

>NM_000942.4 Homo sapiens peptidylprolyl isomerase B (cyclophilin B) (PPIB), mRNA

```
product length = 88
Forward primer  1    TTCCATCGTGTAATCAAGGACTTC  24
Template        446  ........................  469

Reverse primer  1    GCTCACCGTAGATGCTCTTTC  21
Template        533  .....................  513
```

PPIB PrimerCheck (SpliceCenter der Genomics and Bioinformatics Group, LMP, CCR, NCI, http://projects.insilico.us/SpliceCenter/PrimerCheck.jsp)

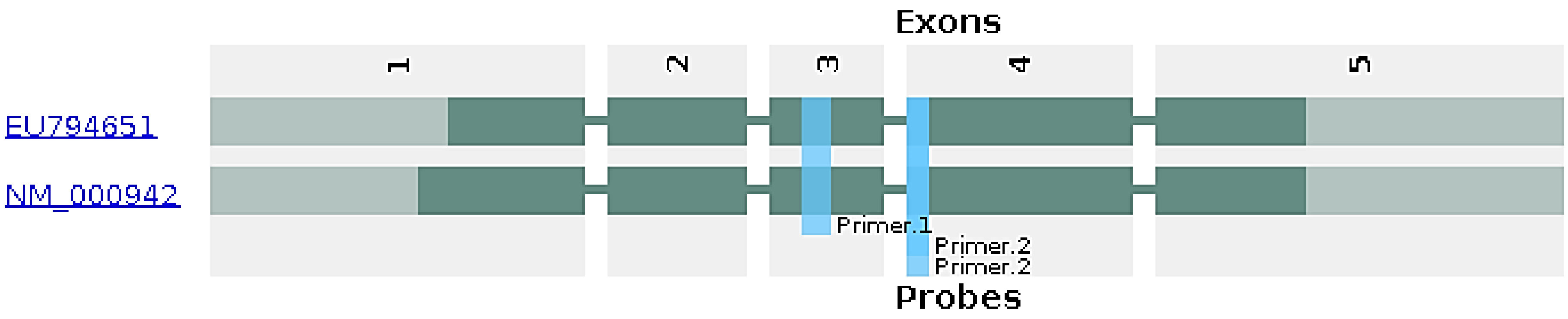

PPIB

PPIB UCSC In-silico-PCR Genome Browser (Dec. 2013 GRCh38/hg38; UCSC Genes; Max. Product Size: 4000; Min. Perfect Match: 15; Min. Good Match: 15; Jim Kent, http://genome-mirror.genomedk.au.dk/cgi-bin/hgPcr)

UCSC In-Silico PCR

The sequences and coordinates shown below are from UCSC Genes, not from the genome assembly. The links lead to the Genome Browser at the position of the entire target sequence.

```
>uc002and.3  PPIB:446+533 88bp TTCCATCGTGTAATCAAGGACTTC GCTCACCGTAGATGCTCTTTC
TTCCATCGTGTAATCAAGGACTTCatgatccagggcggagacttcaccag
gggagatggcacaggagGAAAGAGCATCTACGGTGAGC
```

PPIB UNAFold (Integrated DNA Technologies Inc., Coralville, IA, USA, http://eu.idtdna.com/UNAFold? , Suboptimality 50%)

PPIB Amplicon Sequence 5' TTCCATCGTGTAATCAAGGACTTCATGATCCAGGGCGGAGACTTCACCAGGGGAGATGGCACAGGAGGAAAGAGCATCTACGGTGAGC 3'

Structures

Structure Name	Image	ΔG (kcal.mole^{-1})	T_M (^{o}C)	ΔH (kcal.mole^{-1})	ΔS (cal.K^{-1}mole^{-1})	Output
1	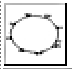	0.45	49	-13.1	-40.66	Ct Det
2	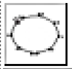	0.64	47.3	-16.1	-50.24	Ct Det
3	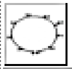	0.66	41.5	-11.2	-35.6	Ct Det
4	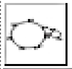	1.25	49	-36.6	-113.63	Ct Det
5	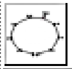	1.32	35.1	-16.4	-53.2	Ct Det

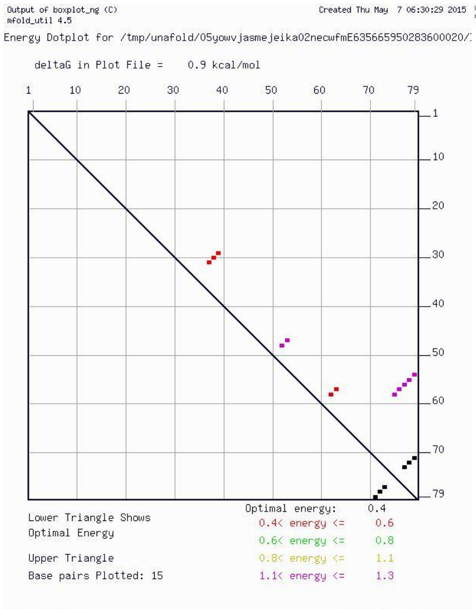

Output of boxplot_ng (C)
mfold_util 4.5
Created Thu May 7 06:30:29 2015
Energy Dotplot for /tmp/unafold/05yowvjasmejeika02necwfmE635665950283600020/
deltaG in Plot File = 0.9 kcal/mol
Optimal energy: 0.4
Lower Triangle Shows
Optimal Energy
0.4< energy <= 0.6
0.6< energy <= 0.8
Upper Triangle
0.8< energy <= 1.1
Base pairs Plotted: 15
1.1< energy <= 1.3

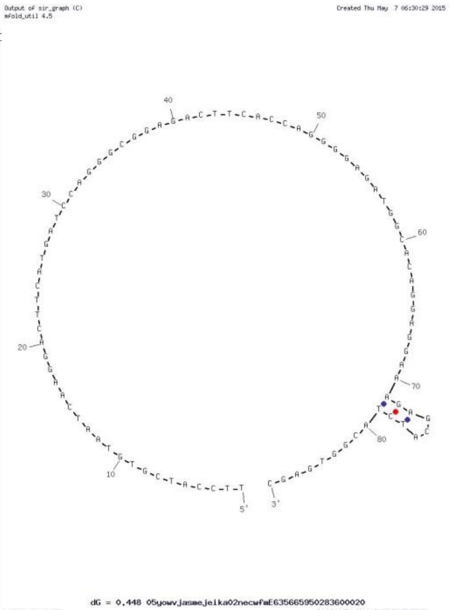

dG = 0.448 05yowvjasmejeika02necwfmE635665950283600020

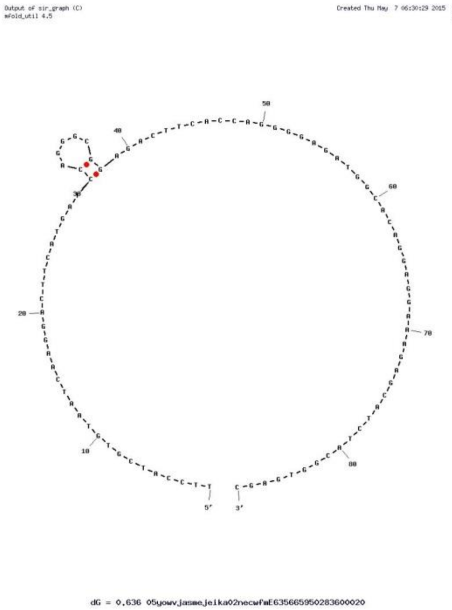

dG = 0.636 05yowvjasmejeika02necwfmE635665950283600020

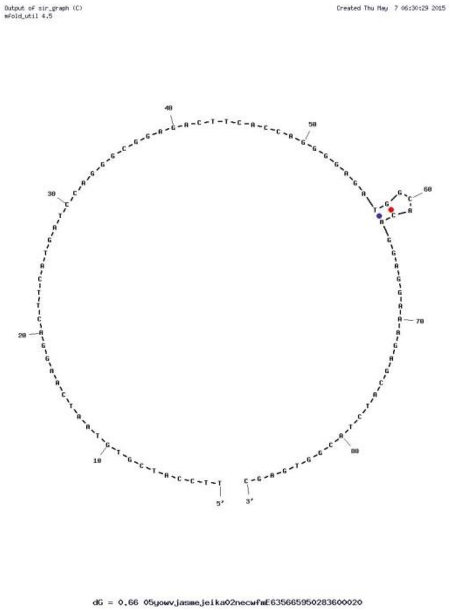

dG = 0.66 05yowvjasmejeika02necwfmE635665950283600020

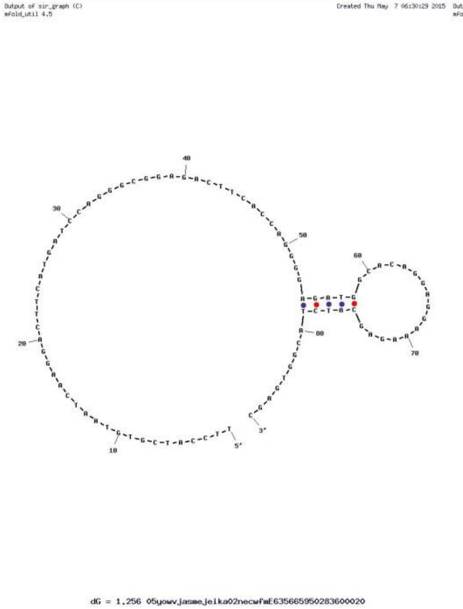

dG = 1.256 05yowvjasmejeika02necwfmE635665950283600020

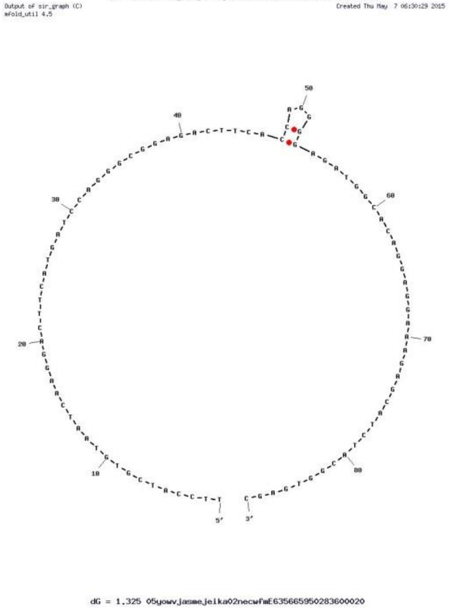

dG = 1.325 05yowvjasmejeika02necwfmE635665950283600020

PPIB BeaconDesigner™ Free Edition (Premier BioSoft International, Palo Alto, CA, USA, http://www.premierbiosoft.com/qOligo/Oligo.jsp?PID=1)

Secondary Structures for Sense Primer

Dimer:-

```
   5' TTCCATCGTGTAATCAAGGACTTC 3'
        ||| ¦  ¦   ¦  ¦ ¦¦¦
3' CTTCAGGAACTAATGTGCTACCTT 5'
                                                             -1.3

5' TTCCATCGTGTAATCAAGGACTTC 3'
                   |||  ¦¦¦
              3' CTTCAGGAACTAATGTGCTACCTT 5'
                                                             -0.5
```

Hairpin:-

```
/GTGCTACCTT 5'
T ¦  ¦ |||
\AATCAAGGACTTC 3'
                                                             -1.3
```

Secondary Structures for Anti-sense Primer

Dimer:-

```
5' GCTCACCGTAGATGCTCTTTC 3'
             |||   ¦¦¦
      3' CTTTCTCGTAGATGCCACTCG 5'
                                                             -0.7
```

Hairpin:-

```
/TAGATGCCACTCG 5'
G |||
\CTCTTTC 3'
                                                             -0.7
```

Cross Dimer

Cross Dimer between Sense Primer and Anti-sense Primer:-

```
    5' TTCCATCGTGTAATCAAGGACTTC 3'
          ||||
3' CTTTCTCGTAGATGCCACTCG 5'
                                                             -2.1

        5' TTCCATCGTGTAATCAAGGACTTC 3'
            ¦¦ ¦   |||
3' CTTTCTCGTAGATGCCACTCG 5'
                                                             -1.3

5' TTCCATCGTGTAATCAAGGACTTC 3'
                  |||  ¦ ¦¦
             3' CTTTCTCGTAGATGCCACTCG 5'
                                                             -0.5

5' TTCCATCGTGTAATCAAGGACTTC 3'
               ||| ¦ ¦
    3' CTTTCTCGTAGATGCCACTCG 5'
                                                             -0.4
```

YWHAZ PrimerBLAST (National Center for Biotechnology Information, Bethesda MD, USA, https://www.ncbi.nlm.nih.gov/tools/primer-blast)

Primer pair 1

	Sequence (5'->3')	Template strand	Length	Start	Stop	Tm	GC%	Self complementarity	Self 3' complementarity
**Forward primer**	AGGAGATTACTACCGTTACTTGGC	Plus	24	504	527	62.43	45.83	4.00	2.00
**Reverse primer**	AGCTTCTTGGTATGCTTGTTGTG	Minus	23	594	572	62.24	43.48	4.00	0.00
**Product length**	91								
**Total intron size**	617 (between pos. 15210694 and 15210076 on NT_008046.17)								

Products on intended target

>NM_003406.3 Homo sapiens tyrosine 3-monooxygenase/tryptophan 5-monooxygenase activation protein, zeta (YWHAZ), transcript variant 1, mRNA

```
product length = 91
Forward primer  1    AGGAGATTACTACCGTTACTTGGC  24
Template        504  ........................  527

Reverse primer  1    AGCTTCTTGGTATGCTTGTTGTG  23
Template        594  .......................  572
```

Products on allowed transcript variants

>XM_011517289.1 PREDICTED: Homo sapiens tyrosine 3-monooxygenase/tryptophan 5-monooxygenase activation protein, zeta (YWHAZ), transcript variant X4, mRNA

```
product length = 91
Forward primer  1    AGGAGATTACTACCGTTACTTGGC  24
Template        809  ........................  832

Reverse primer  1    AGCTTCTTGGTATGCTTGTTGTG  23
Template        899  .......................  877
```

>XM_005251063.2 PREDICTED: Homo sapiens tyrosine 3-monooxygenase/tryptophan 5-monooxygenase activation protein, zeta (YWHAZ), transcript variant X3, mRNA

```
product length = 91
Forward primer  1    AGGAGATTACTACCGTTACTTGGC  24
Template        663  ........................  686

Reverse primer  1    AGCTTCTTGGTATGCTTGTTGTG  23
Template        753  .......................  731
```

>XM_005251062.2 PREDICTED: Homo sapiens tyrosine 3-monooxygenase/tryptophan 5-monooxygenase activation protein, zeta (YWHAZ), transcript variant X2, mRNA

```
product length = 91
Forward primer  1    AGGAGATTACTACCGTTACTTGGC  24
Template        676  ........................  699

Reverse primer  1    AGCTTCTTGGTATGCTTGTTGTG  23
Template        766  .......................  744
```

>XM_005251061.2 PREDICTED: Homo sapiens tyrosine 3-monooxygenase/tryptophan 5-monooxygenase activation protein, zeta (YWHAZ), transcript variant X1, mRNA

```
product length = 91
Forward primer  1    AGGAGATTACTACCGTTACTTGGC  24
Template        901  ........................  924

Reverse primer  1    AGCTTCTTGGTATGCTTGTTGTG  23
Template        991  .......................  969
```

>NM_001135702.1 Homo sapiens tyrosine 3-monooxygenase/tryptophan 5-monooxygenase activation protein, zeta (YWHAZ), transcript variant 6, mRNA

```
product length = 91
Forward primer  1    AGGAGATTACTACCGTTACTTGGC  24
Template        543  ........................  566

Reverse primer  1    AGCTTCTTGGTATGCTTGTTGTG  23
Template        633  .......................  611
```

>NM_001135701.1 Homo sapiens tyrosine 3-monooxygenase/tryptophan 5-monooxygenase activation protein, zeta (YWHAZ), transcript variant 5, mRNA

```
product length = 91
Forward primer  1    AGGAGATTACTACCGTTACTTGGC  24
Template        524  ........................  547

Reverse primer  1    AGCTTCTTGGTATGCTTGTTGTG  23
Template        614  .......................  592
```

>NM_001135700.1 Homo sapiens tyrosine 3-monooxygenase/tryptophan 5-monooxygenase activation protein, zeta (YWHAZ), transcript variant 4, mRNA

```
product length = 91
Forward primer  1    AGGAGATTACTACCGTTACTTGGC  24
Template        475  ........................  498

Reverse primer  1    AGCTTCTTGGTATGCTTGTTGTG  23
Template        565  .......................  543
```

>NM_001135699.1 Homo sapiens tyrosine 3-monooxygenase/tryptophan 5-monooxygenase activation protein, zeta (YWHAZ), transcript variant 3, mRNA

```
product length = 91
Forward primer  1    AGGAGATTACTACCGTTACTTGGC  24
Template        521  ........................  544

Reverse primer  1    AGCTTCTTGGTATGCTTGTTGTG  23
Template        611  .......................  589
```

>NM_145690.2 Homo sapiens tyrosine 3-monooxygenase/tryptophan 5-monooxygenase activation protein, zeta (YWHAZ), transcript variant 2, mRNA

```
product length = 91
Forward primer  1    AGGAGATTACTACCGTTACTTGGC  24
Template        578  ........................  601

Reverse primer  1    AGCTTCTTGGTATGCTTGTTGTG  23
Template        668  .......................  646
```

YWHAZ PrimerCheck (SpliceCenter der Genomics and Bioinformatics Group, LMP, CCR, NCI, http://projects.insilico.us/SpliceCenter/PrimerCheck.jsp)

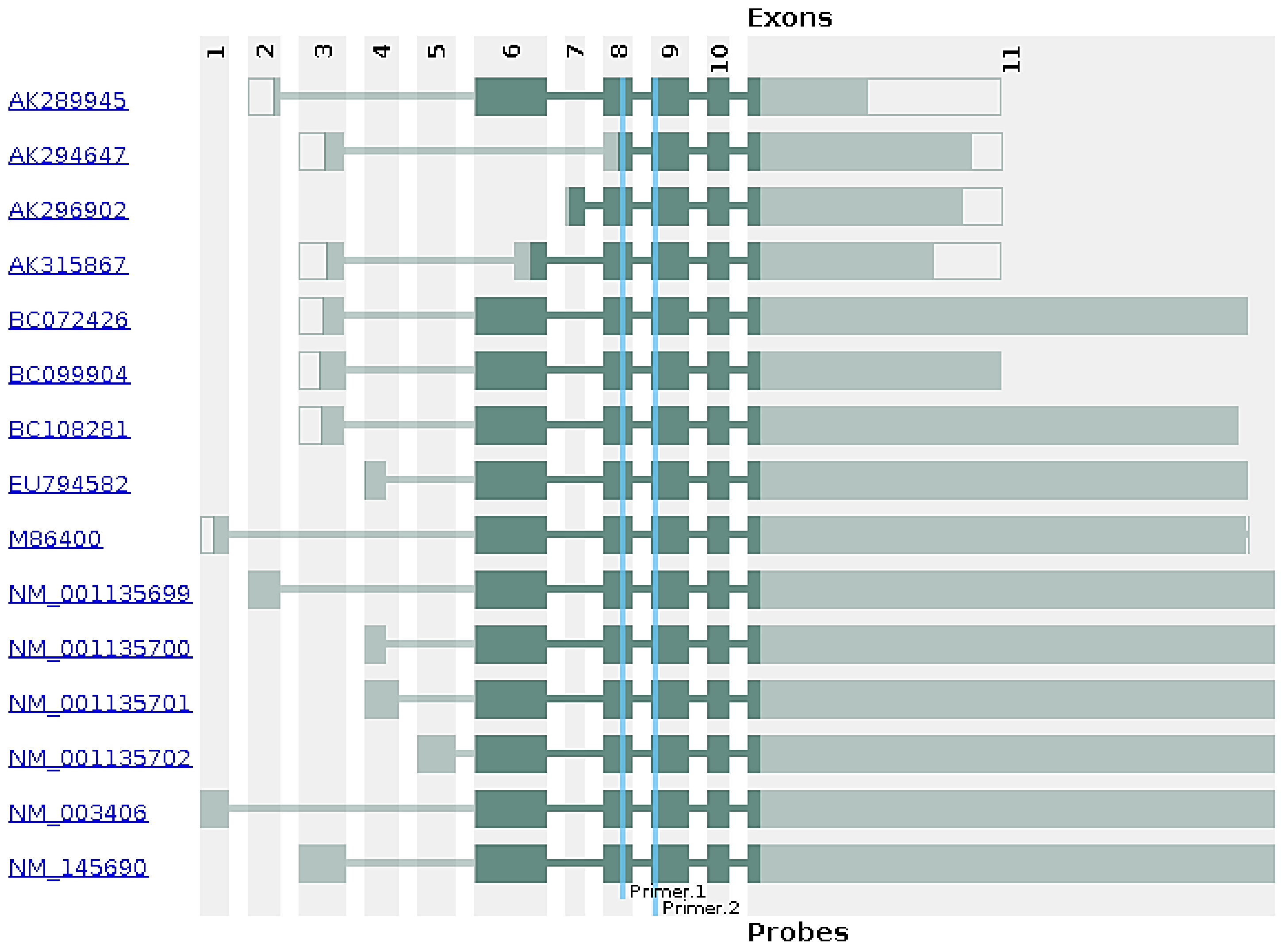

YWHAZ

YWHAZ UCSC In-silico-PCR Genome Browser (Dec. 2013 GRCh38/hg38; UCSC Genes; Max. Product Size: 4000; Min. Perfect Match: 15; Min. Good Match: 15; Jim Kent, http://genome-mirror.genomedk.au.dk/cgi-bin/hgPcr)

UCSC In-Silico PCR

The sequences and coordinates shown below are from UCSC Genes, not from the genome assembly. The links lead to the Genome Browser at the position of the entire target sequence.

```
>uc011lhe.1  YWHAZ:543+633 91bp AGGAGATTACTACCGTTACTTGGC AGCTTCTTGGTATGCTTGTTGTG
AGGAGATTACTACCGTTACTTGGCtgaggttgccgctggtgatgacaaga
aagggattgtcgatcagtCACAACAAGCATACCAAGAAGCT

>uc011lhf.1  YWHAZ:524+614 91bp AGGAGATTACTACCGTTACTTGGC AGCTTCTTGGTATGCTTGTTGTG
AGGAGATTACTACCGTTACTTGGCtgaggttgccgctggtgatgacaaga
aagggattgtcgatcagtCACAACAAGCATACCAAGAAGCT

>uc011lhg.1  YWHAZ:273+363 91bp AGGAGATTACTACCGTTACTTGGC AGCTTCTTGGTATGCTTGTTGTG
AGGAGATTACTACCGTTACTTGGCtgaggttgccgctggtgatgacaaga
aagggattgtcgatcagtCACAACAAGCATACCAAGAAGCT

>uc003yjv.2  YWHAZ:475+565 91bp AGGAGATTACTACCGTTACTTGGC AGCTTCTTGGTATGCTTGTTGTG
AGGAGATTACTACCGTTACTTGGCtgaggttgccgctggtgatgacaaga
aagggattgtcgatcagtCACAACAAGCATACCAAGAAGCT

>uc010mbq.2  YWHAZ:410+500 91bp AGGAGATTACTACCGTTACTTGGC AGCTTCTTGGTATGCTTGTTGTG
AGGAGATTACTACCGTTACTTGGCtgaggttgccgctggtgatgacaaga
aagggattgtcgatcagtCACAACAAGCATACCAAGAAGCT

>uc003yjw.2  YWHAZ:578+668 91bp AGGAGATTACTACCGTTACTTGGC AGCTTCTTGGTATGCTTGTTGTG
AGGAGATTACTACCGTTACTTGGCtgaggttgccgctggtgatgacaaga
aagggattgtcgatcagtCACAACAAGCATACCAAGAAGCT

>uc010mbr.2  YWHAZ:521+611 91bp AGGAGATTACTACCGTTACTTGGC AGCTTCTTGGTATGCTTGTTGTG
AGGAGATTACTACCGTTACTTGGCtgaggttgccgctggtgatgacaaga
aagggattgtcgatcagtCACAACAAGCATACCAAGAAGCT

>uc003yjx.2  YWHAZ:504+594 91bp AGGAGATTACTACCGTTACTTGGC AGCTTCTTGGTATGCTTGTTGTG
AGGAGATTACTACCGTTACTTGGCtgaggttgccgctggtgatgacaaga
aagggattgtcgatcagtCACAACAAGCATACCAAGAAGCT
```

YWHAZ UNAFold (Integrated DNA Technologies Inc., Coralville, IA, USA, http://eu.idtdna.com/UNAFold? , Suboptimality 50%)

YWHAZ Amplicon Sequence

5’ AGGAGATTACTACCGTTACTTGGCTGAGGTTGCCGCTGGTGATGACAAGAAAGGGATTGTCGATCAGTCACAACAAGCATACCAAGAAGCT 3’

Structures

Structure Name	Image	ΔG (kcal.mole^{-1})	T_M (^oC)	ΔH (kcal.mole^{-1})	ΔS (cal.K^{-1}mole^{-1})	Output
1	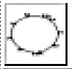	0.98	32.5	-10.9	-35.66	Ct Det
2	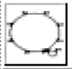	1.03	41.9	-18	-57.14	Ct Det
3	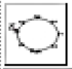	1.06	46.6	-25.3	-79.13	Ct Det
4	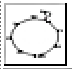	1.3	41.4	-22	-69.94	Ct Det
5	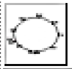	1.47	35.7	-18.6	-60.23	Ct Det
6	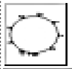	1.52	26	-13.4	-44.79	Ct Det
7	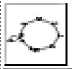	1.57	39	-23.3	-74.64	Ct Det
8	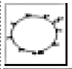	1.74	41.1	-28.9	-91.96	Ct Det
9	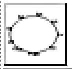	1.88	18.8	-13.3	-45.56	Ct Det

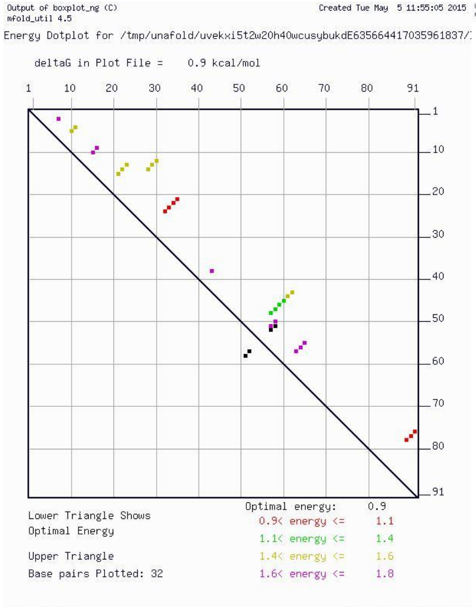
Output of boxplot_ng (C)
mfold_util 4.5
Created Tue May 5 11:55:05 2015
Energy Dotplot for /tmp/unafold/uvekxi5t2w20h40wcusybukdE635664417035961837/
deltaG in Plot File = 0.9 kcal/mol
Optimal energy: 0.9
Lower Triangle Shows
Optimal Energy
0.9< energy <= 1.1
1.1< energy <= 1.4
Upper Triangle
1.4< energy <= 1.6
Base pairs Plotted: 32
1.6< energy <= 1.8

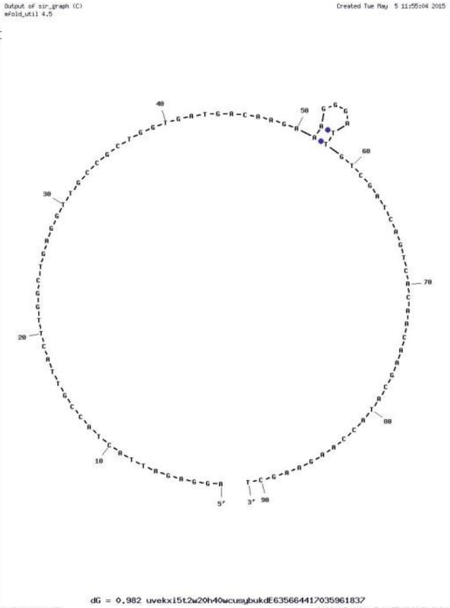
dG = 0.982 uvekxi5t2w20h40wcusybukdE635664417035961837

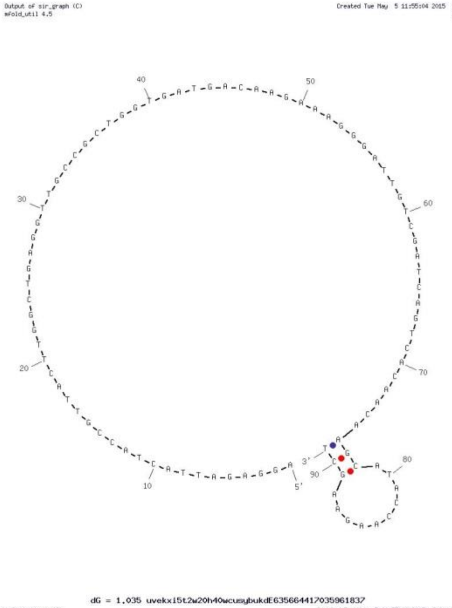
dG = 1.035 uvekxi5t2w20h40wcusybukdE635664417035961837

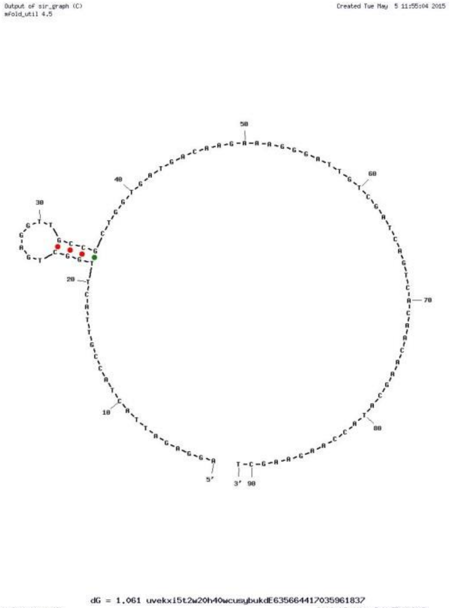
dG = 1.061 uvekxi5t2w20h40wcusybukdE635664417035961837

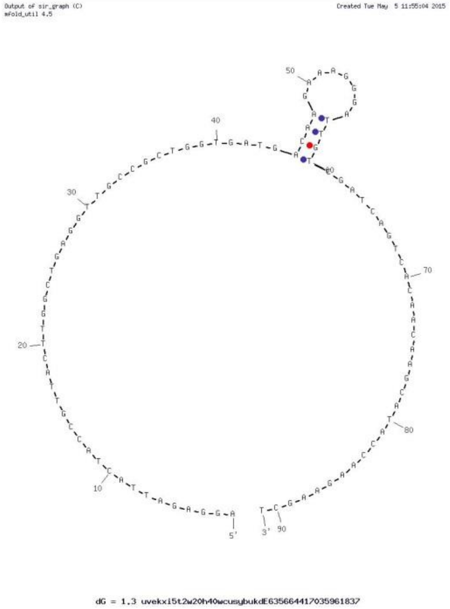
dG = 1.3 uvekxi5t2w20h40wcusybukdE635664417035961837

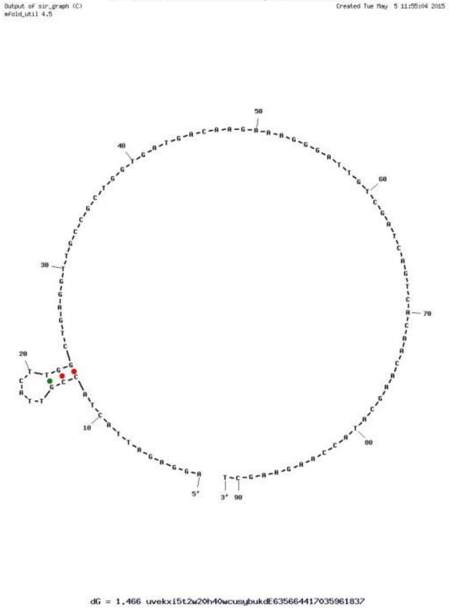
dG = 1.466 uvekxi5t2w20h40wcusybukdE635664417035961837

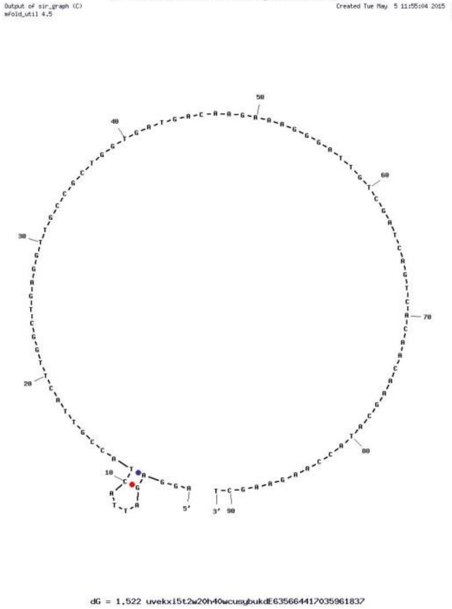
dG = 1.522 uvekxi5t2w20h40wcusybukdE635664417035961837

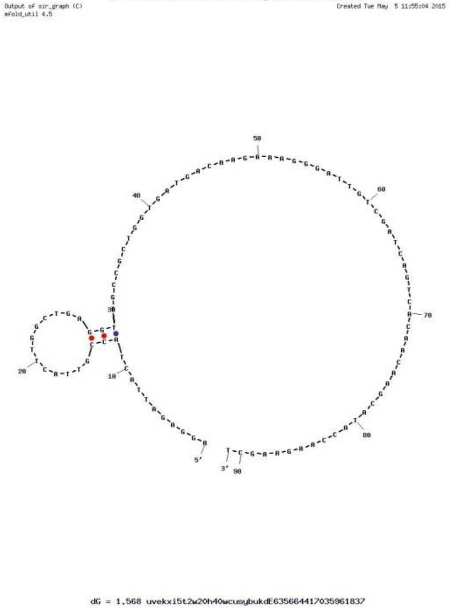
dG = 1.568 uvekxi5t2w20h40wcusybukdE635664417035961837

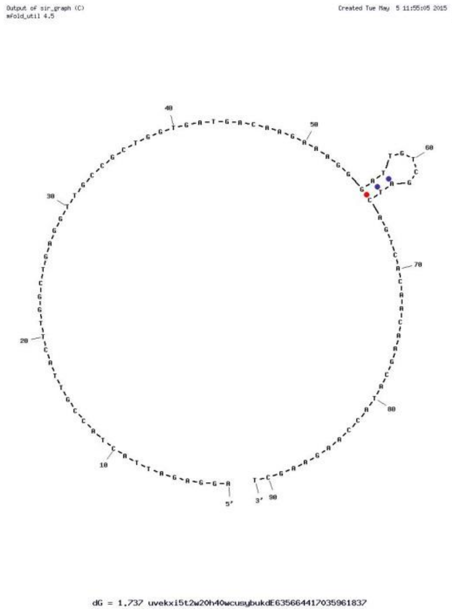
Output of sir_graph (C)
mfold_util 4.5
Created Tue May 5 11:55:05 2015
5'
3'
dG = 1,737 uvekxi5t2w20h40wcusybukdE635664417035961837

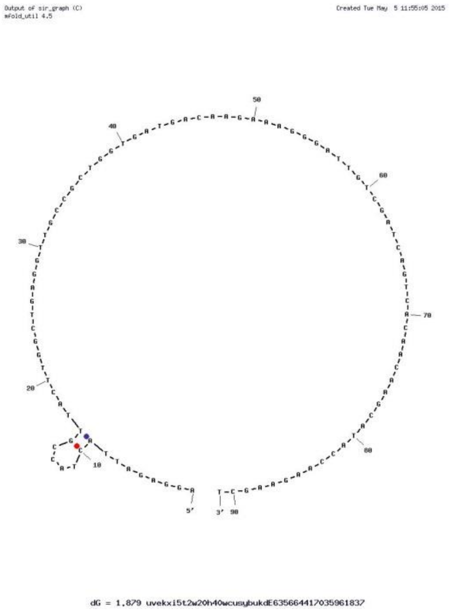
Output of sir_graph (C)
mfold_util 4.5
Created Tue May 5 11:55:05 2015
5'
3'
dG = 1,879 uvekxi5t2w20h40wcusybukdE635664417035961837

YWHAZ BeaconDesigner™ Free Edition (Premier BioSoft International, Palo Alto, CA, USA, http://www.premierbiosoft.com/qOligo/Oligo.jsp?PID=1)

Secondary Structures for Sense Primer

Dimer:-

Not Found

Hairpin:-

Not Found

Secondary Structures for Anti-sense Primer

Dimer:-

```
                   5' AGCTTCTTGGTATGCTTGTTGTG 3'
                      ||||
3' GTGTTGTTCGTATGGTTCTTCGA 5'
                                                    -3.0

       5' AGCTTCTTGGTATGCTTGTTGTG 3'
          ||| ¦      ¦ ¦¦¦
3' GTGTTGTTCGTATGGTTCTTCGA 5'
                                                    -1.8
```

Hairpin:-

```
/TTCTTCGA 5'
|   ¦ |||
\GGTATGCTTGTTGTG 3'
                                                    -1.8
```

Cross Dimer

Cross Dimer between Sense Primer and Anti-sense Primer:-

```
 5' AGGAGATTACTACCGTTACTTGGC 3'
    ¦  ¦ ¦   ||||   ¦
3' GTGTTGTTCGTATGGTTCTTCGA 5'
                                                    -2.2

            5' AGGAGATTACTACCGTTACTTGGC 3'
                  |||
3' GTGTTGTTCGTATGGTTCTTCGA 5'
                                                    -0.7

5' AGGAGATTACTACCGTTACTTGGC 3'
          ¦  ¦    |||   ¦
     3' GTGTTGTTCGTATGGTTCTTCGA 5'
                                                    -0.3

    5' AGGAGATTACTACCGTTACTTGGC 3'
       ¦  ¦¦  ||| ¦
3' GTGTTGTTCGTATGGTTCTTCGA 5'
                                                    -0.3
```

POLR2A PrimerBLAST (National Center for Biotechnology Information, Bethesda MD, USA, https://www.ncbi.nlm.nih.gov/tools/primer-blast)

Primer pair 1

	Sequence (5'->3')	Template strand	Length	Start	Stop	Tm	GC%	Self complementarity	Self 3' complementarity
**Forward primer**	TCGCTTACTGTCTTCCTGTTGG	Plus	22	3798	3819	62.77	50.00	3.00	0.00
**Reverse primer**	TGTGTTGGCAGTCACCTTCC	Minus	20	3905	3886	62.95	55.00	3.00	3.00
**Product length**	108								
**Total intron size**	468 (between pos. 7019488 and 7019957 on NT_010718.17)								

Products on intended target

>NM_000937.4 Homo sapiens polymerase (RNA) II (DNA directed) polypeptide A, 220kDa (POLR2A), mRNA

```
product length = 108
Forward primer  1     TCGCTTACTGTCTTCCTGTTGG  22
Template        3798  ......................  3819

Reverse primer  1     TGTGTTGGCAGTCACCTTCC  20
Template        3905  ....................  3886
```

POLR2A PrimerCheck (SpliceCenter der Genomics and Bioinformatics Group, LMP, CCR, NCI, http://projects.insilico.us/SpliceCenter/PrimerCheck.jsp)

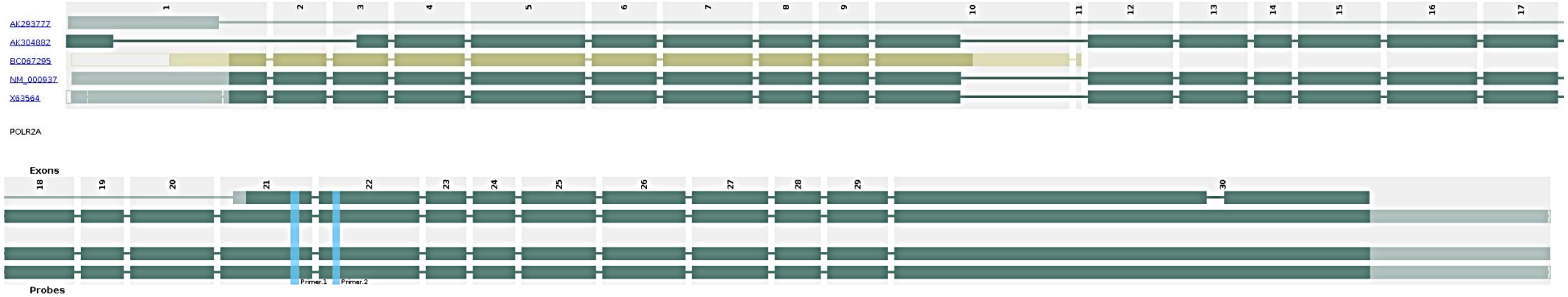

POLR2A UCSC In-silico-PCR Genome Browser (Dec. 2013 GRCh38/hg38; UCSC Genes; Max. Product Size: 4000; Min. Perfect Match: 15; Min. Good Match: 15; Jim Kent, http://genome-mirror.genomedk.au.dk/cgi-bin/hgPcr)

UCSC In-Silico PCR

The sequences and coordinates shown below are from UCSC Genes, not from the genome assembly. The links lead to the Genome Browser at the position of the entire target sequence.

```
>uc032eso.1  POLR2A:3798+3905 108bp TCGCTTACTGTCTTCCTGTTGG TGTGTTGGCAGTCACCTTCC
TCGCTTACTGTCTTCCTGTTGGgccagtccgctcgagatgctgagagagc
caaggatattctgtgccgtctggagcatacaacgttgaGGAAGGTGACTG
CCAACACA
```

POLR2A UNAFold (Integrated DNA Technologies Inc., Coralville, IA, USA, http://eu.idtdna.com/UNAFold? , Suboptimality 50%)

POLR2A Amplicon Sequence

5' TCGCTTACTGTCTTCCTGTTGGGCCAGTCCGCTCGAGATGCTGAGAGAGCCAAGGATATTCTGTGCCGTCTGGAGCATACAACGTTGAGGAAGGTGACTGCCAACACA 3'

Structures

Structure Name	Image	ΔG (kcal.mole^{-1})	T_M (^oC)	ΔH (kcal.mole^{-1})	ΔS (cal.K^{-1}mole^{-1})	Output
1	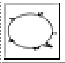	0.11	58.7	-28	-84.38	Ct Det
2	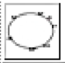	0.63	48.7	-18.1	-56.23	Ct Det
3	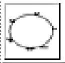	0.64	50.7	-22.2	-68.55	Ct Det
4	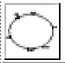	0.83	49.9	-26.6	-82.35	Ct Det

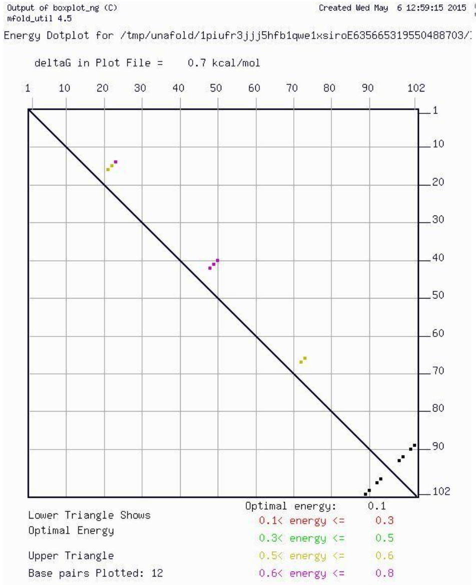

Output of boxplot_ng (C)
mfold_util 4.5
Created Wed May 6 12:59:15 2015
Energy Dotplot for /tmp/unafold/1piufr3jjj5hfb1qwe1xsiroE635665319550488703/
deltaG in Plot File = 0.7 kcal/mol
Optimal energy: 0.1
Lower Triangle Shows
Optimal Energy
0.1< energy <= 0.3
0.3< energy <= 0.5
Upper Triangle
0.5< energy <= 0.6
Base pairs Plotted: 12
0.6< energy <= 0.8

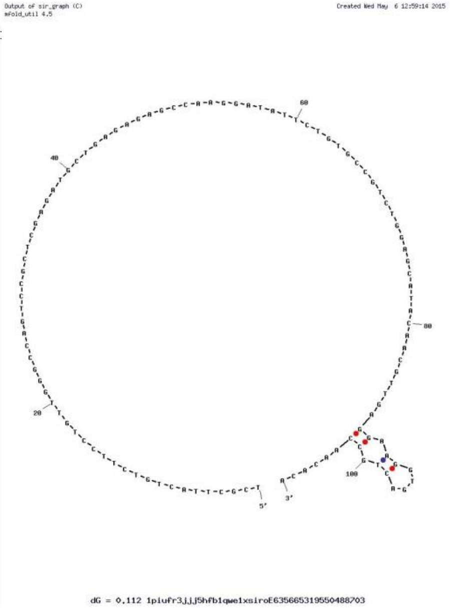

Output of sir_graph (C)
mfold_util 4.5
Created Wed May 6 12:59:14 2015
dG = 0.112 1piufr3jjj5hfb1qwe1xsiroE635665319550488703

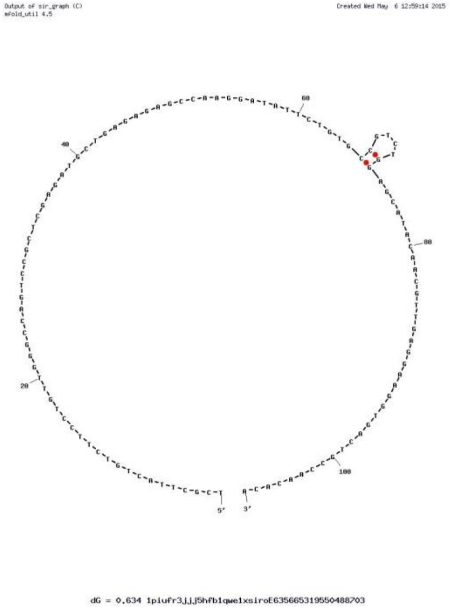

Output of sir_graph (C)
mfold_util 4.5
Created Wed May 6 12:59:14 2015
dG = 0.634 1piufr3jjj5hfb1qwe1xsiroE635665319550488703

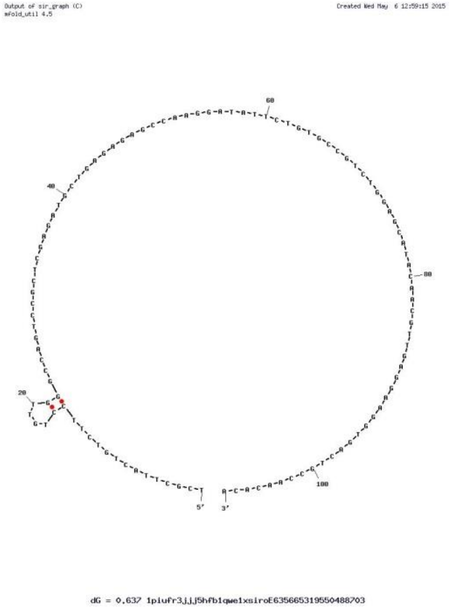

Output of sir_graph (C)
mfold_util 4.5
Created Wed May 6 12:59:15 2015
dG = 0.637 1piufr3jjj5hfb1qwe1xsiroE635665319550488703

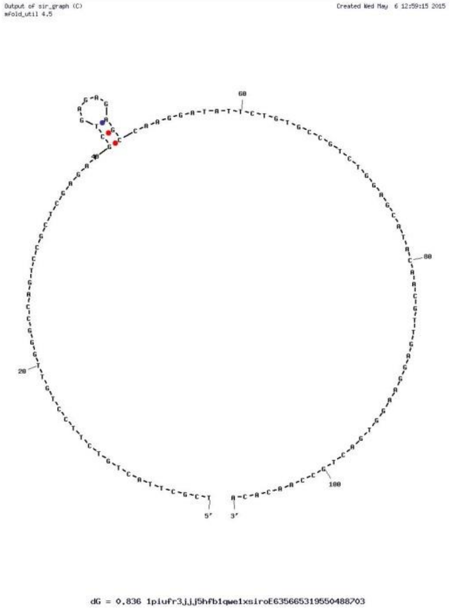

Output of sir_graph (C)
mfold_util 4.5
Created Wed May 6 12:59:15 2015
dG = 0.836 1piufr3jjj5hfb1qwe1xsiroE635665319550488703

POLR2A BeaconDesigner™ Free Edition (Premier BioSoft International, Palo Alto, CA, USA, http://www.premierbiosoft.com/qOligo/Oligo.jsp?PID=1)

Secondary Structures for Sense Primer

Dimer:-

Not Found

Hairpin:-

Not Found

Secondary Structures for Anti-sense Primer

Dimer:-

```
    5' TGTGTTGGCAGTCACCTTCC 3'
           |||   ::   :::
3' CCTTCCACTGACGGTTGTGT 5'
                                                          -1.3
```

Hairpin:-

```
/GGTTGTGT 5'
|    |||
\CAGTCACCTTCC 3'
                                                          -1.3
```

Cross Dimer

Cross Dimer between Sense Primer and Anti-sense Primer:-

```
  5' TCGCTTACTGTCTTCCTGTTGG 3'
          : : |||| :   :
3' CCTTCCACTGACGGTTGTGT 5'
                                                          -2.5

5' TCGCTTACTGTCTTCCTGTTGG 3'
                 :   |||
      3' CCTTCCACTGACGGTTGTGT 5'
                                                          -1.1
```

TBP PrimerBLAST (National Center for Biotechnology Information, Bethesda MD, USA, https://www.ncbi.nlm.nih.gov/tools/primer-blast)

Primer pair 1

	Sequence (5'->3')	Template strand	Length	Start	Stop	Tm	GC%	Self complementarity	Self 3' complementarity
**Forward primer**	CGGCTGTTTAACTTCGCTTCC	Plus	21	79	99	62.54	52.38	5.00	0.00
**Reverse primer**	TGGGTTATCTTCACACGCCAAG	Minus	22	164	143	63.37	50.00	3.00	2.00
**Product length**	86								
**Total intron size**	2418 (between pos. 110324529 and 110326948 on NT_025741.16)								

Products on intended target

>NM_003194.4 Homo sapiens TATA box binding protein (TBP), transcript variant 1, mRNA

```
product length = 86
Forward primer  1    CGGCTGTTTAACTTCGCTTCC  21
Template        79   .....................  99

Reverse primer  1    TGGGTTATCTTCACACGCCAAG  22
Template        164  ......................  143
```

TBP UCSC In-silico-PCR Genome Browser (Dec. 2013 GRCh38/hg38; UCSC Genes; Max. Product Size: 4000; Min. Perfect Match: 15; Min. Good Match: 15; Jim Kent, http://genome-mirror.genomedk.au.dk/cgi-bin/hgPcr)

UCSC In-Silico PCR

The sequences and coordinates shown below are from UCSC Genes, not from the genome assembly. The links lead to the Genome Browser at the position of the entire target sequence.

```
>uc003qxu.3  TBP:79+164 86bp CGGCTGTTTAACTTCGCTTCC TGGGTTATCTTCACACGCCAAG
CGGCTGTTTAACTTCGCTTCCgctggcccatagtgatctttgcagtgacc
cagcatcactgtttCTTGGCGTGTGAAGATAACCCA

>uc003qxt.3  TBP:79+167 89bp CGGCTGTTTAACTTCGCTTCC TGGGTTATCTTCACACGCCAAG
CGGCTGTTTAACTTCGCTTCCgctggcccatagtgatctttgcagtgacc
cagcagcatcactgtttCTTGGCGTGTGAAGATAACCCA
```

TBP PrimerCheck (SpliceCenter der Genomics and Bioinformatics Group, LMP, CCR, NCI, http://projects.insilico.us/SpliceCenter/PrimerCheck.jsp)

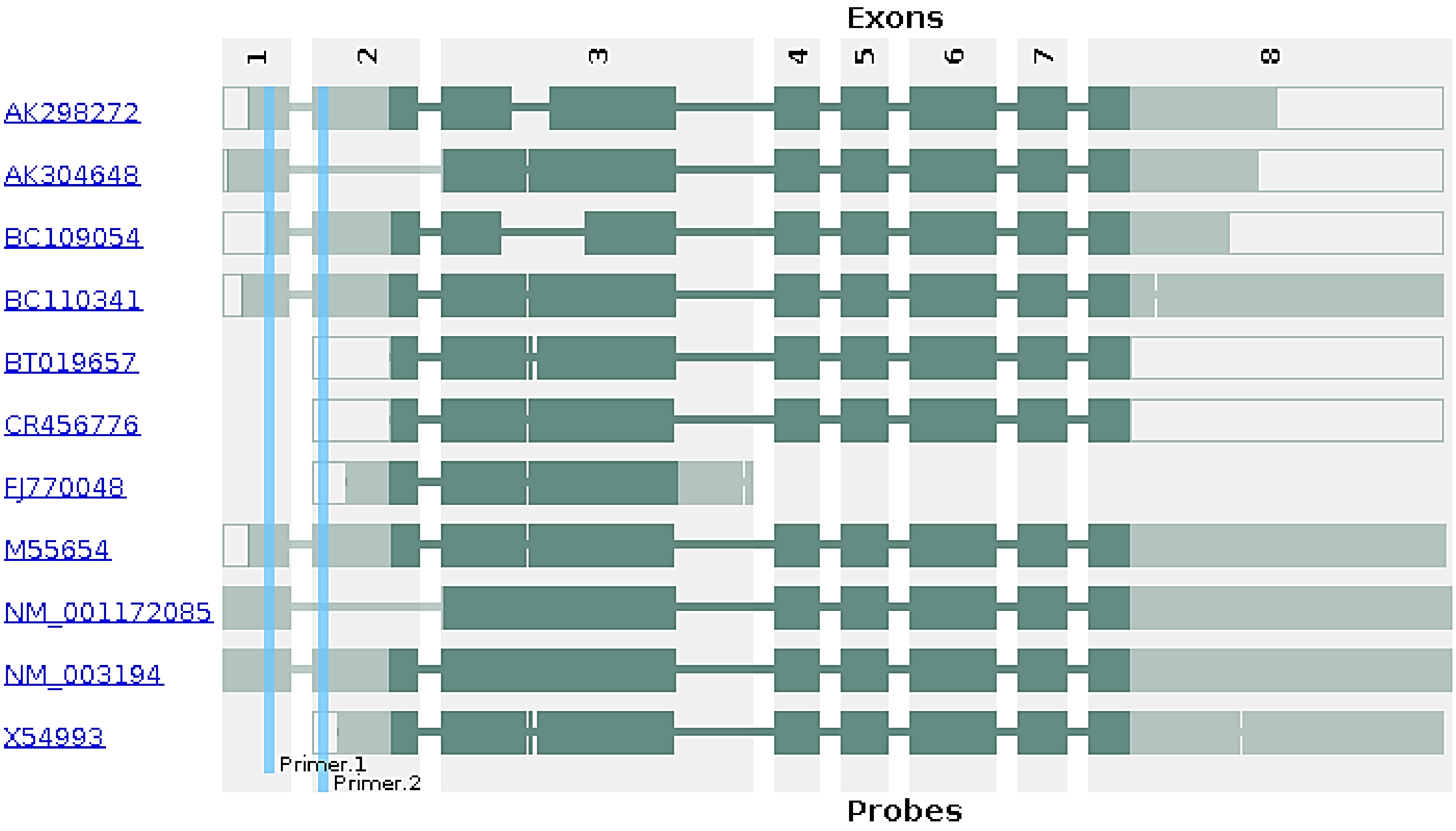

TBP

TBP UNAFold (Integrated DNA Technologies Inc., Coralville, IA, USA, http://eu.idtdna.com/UNAFold? , Suboptimality 50%)

TBP Amplicon Sequence

5' CGGCTGTTTAACTTCGCTTCCGCTGGCCCATAGTGATCTTTGCAGTGACCCAGCATCACTGTTTCTTGGCGTGTGAAGATAACCCA 3'

Structures

Structure Name	Image	ΔG (kcal.mole^{-1})	T_M (^{o}C)	ΔH (kcal.mole^{-1})	ΔS (cal.K^{-1}mole^{-1})	Output
1	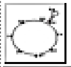	0.26	58.1	-46.1	-139.16	Ct Det
2	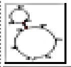	0.89	53.2	-42.5	-130.23	Ct Det
3	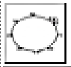	1.15	44.4	-23.5	-74	Ct Det

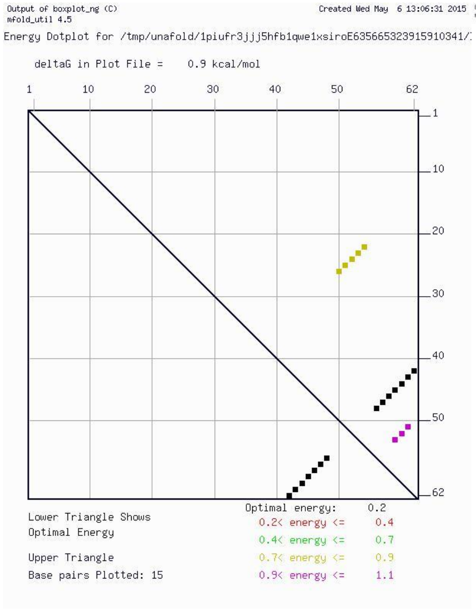

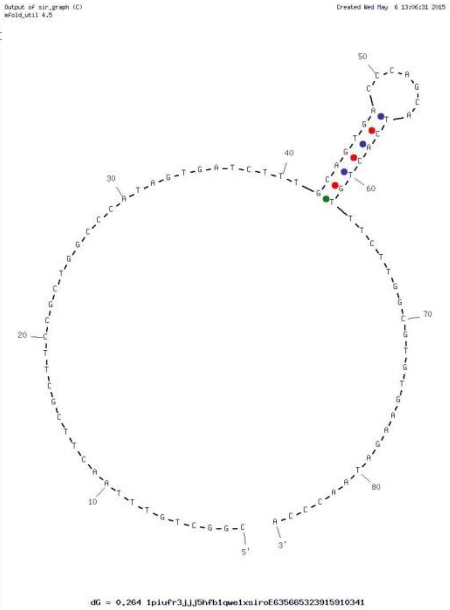

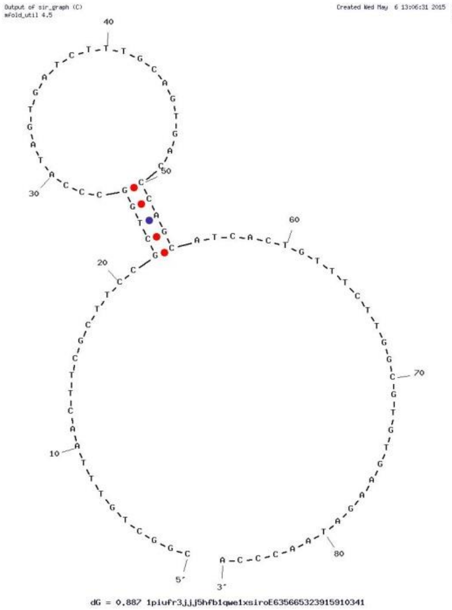

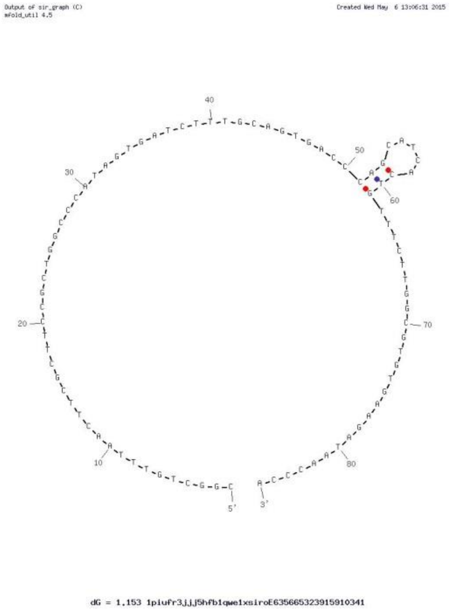

TBP BeaconDesigner™ Free Edition (Premier BioSoft International, Palo Alto, CA, USA, http://www.premierbiosoft.com/qOligo/Oligo.jsp?PID=1)

Secondary Structures for Sense Primer

Dimer:-

```
   5' CGGCTGTTTAACTTCGCTTCC 3'
       ¦     ||||     ¦
3' CCTTCGCTTCAATTTGTCGGC 5'
                                                                    -0.8

    5' CGGCTGTTTAACTTCGCTTCC 3'
         ¦  ||| ¦¦¦  ¦
3' CCTTCGCTTCAATTTGTCGGC 5'
                                                                    -0.7
```

Hairpin:-

Not Found

Secondary Structures for Anti-sense Primer

Dimer:-

```
  5' TGGGTTATCTTCACACGCCAAG 3'
     |||  ¦ ¦    ¦ ¦  ¦¦¦
3' GAACCGCACACTTCTATTGGGT 5'
                                                                    -1.5

5' TGGGTTATCTTCACACGCCAAG 3'
           |||  ¦  ¦  ¦¦¦
        3' GAACCGCACACTTCTATTGGGT 5'
                                                                    -0.5
```

Hairpin:-

```
/TCTATTGGGT 5'
|  ¦ ¦  |||
\TCACACGCCAAG 3'
                                                                    -1.5

/ACACTTCTATTGGGT 5'
| ¦  |||
\CGCCAAG 3'
                                                                    -0.5
```

Cross Dimer

Cross Dimer between Sense Primer and Anti-sense Primer:-

```
  5' CGGCTGTTTAACTTCGCTTCC 3'
      |||   ¦ ¦¦  ¦  ¦
3' GAACCGCACACTTCTATTGGGT 5'
                                                                    -2.4

       5' CGGCTGTTTAACTTCGCTTCC 3'
           ¦      ||||
3' GAACCGCACACTTCTATTGGGT 5'
                                                                    -1.4

   5' CGGCTGTTTAACTTCGCTTCC 3'
       ¦  |||  ¦  ¦   ¦
3' GAACCGCACACTTCTATTGGGT 5'
                                                                    -1.0

5' CGGCTGTTTAACTTCGCTTCC 3'
                   |||
                3' GAACCGCACACTTCTATTGGGT 5'
                                                                    -0.5

5' CGGCTGTTTAACTTCGCTTCC 3'
              ||| ¦¦ ¦
           3' GAACCGCACACTTCTATTGGGT 5'
                                                                    -0.5
```

RPL22 PrimerBLAST (National Center for Biotechnology Information, Bethesda MD, USA, https://www.ncbi.nlm.nih.gov/tools/primer-blast)

Primer pair 1

	Sequence (5'->3')	Template strand	Length	Start	Stop	Tm	GC%	Self complementarity	Self 3' complementarity
**Forward primer**	TGATTGCACCCACCCTGTAG	Plus	20	115	134	62.18	55.00	4.00	2.00
**Reverse primer**	GGTTCCCAGCTTTTCCGTTC	Minus	20	212	193	61.84	55.00	4.00	0.00
**Product length**	98								
**Total intron size**	4597 (between pos. 5611664 and 5607066 on NT_032977.10)								

Products on intended target

>NM_000983.3 Homo sapiens ribosomal protein L22 (RPL22), mRNA

```
product length = 98
Forward primer  1    TGATTGCACCCACCCTGTAG  20
Template        115  ....................  134

Reverse primer  1    GGTTCCCAGCTTTTCCGTTC  20
Template        212  ....................  193
```

RPL22 UCSC In-silico-PCR Genome Browser (Dec. 2013 GRCh38/hg38; UCSC Genes; Max. Product Size: 4000; Min. Perfect Match: 15; Min. Good Match: 15; Jim Kent, http://genome-mirror.genomedk.au.dk/cgi-bin/hgPcr).

UCSC In-Silico PCR

The sequences and coordinates shown below are from UCSC Genes, not from the genome assembly. The links lead to the Genome Browser at the position of the entire target sequence.

```
>uc001amd.3__RPL22:115+212 98bp TGATTGCACCCACCCTGTAG GGTTCCCAGCTTTTCCGTTC
TGATTGCACCCACCCTGTAGaagatggaatcatggatgctgccaattttg
agcagtttttgcaagaaaggatcaaagtGAACGGAAAAGCTGGGAACC

>uc001ame.3__RPL22:115+212 98bp TGATTGCACCCACCCTGTAG GGTTCCCAGCTTTTCCGTTC
TGATTGCACCCACCCTGTAGaagatggaatcatggatgctgccaattttg
agcagtttttgcaagaaaggatcaaagtGAACGGAAAAGCTGGGAACC
```

RPL22 PrimerCheck (SpliceCenter der Genomics and Bioinformatics Group, LMP, CCR, NCI, http://projects.insilico.us/SpliceCenter/PrimerCheck.jsp)

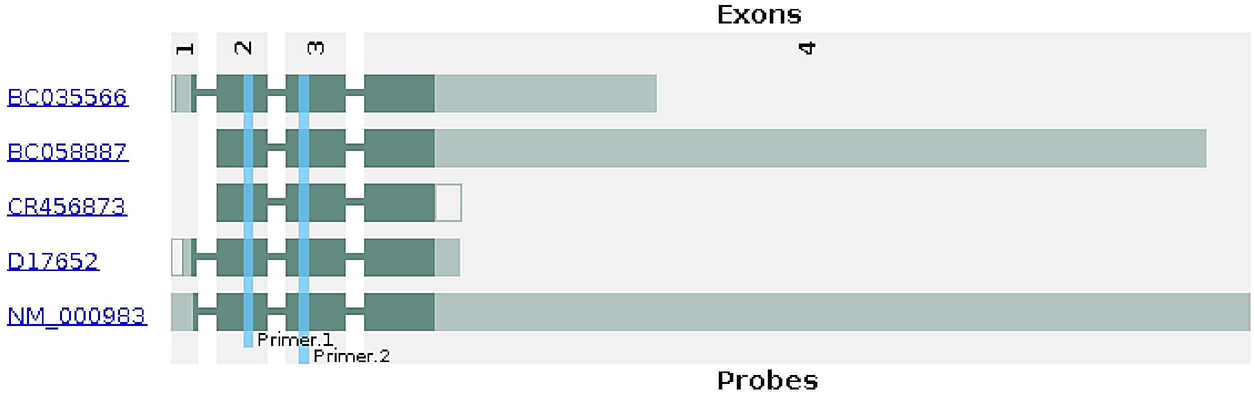

RPL22

RPL22 UNAFold (Integrated DNA Technologies Inc., Coralville, IA, USA, http://eu.idtdna.com/UNAFold? , Suboptimality 50%)

RPL22 Amplicon Sequence

5’ TGATTGCACCCACCCTGTAGAAGATGGAATCATGGATGCTGCCAATTTTGAGCAGTTTTTGCAAGAAAGGATCAAAGTGAACGGAAAAGCTGGGAACC 3’

Structures

Structure Name	Image	ΔG (kcal.mole^{-1})	T_M (^oC)	ΔH (kcal.mole^{-1})	ΔS (cal.K^{-1}mole^{-1})	Output
1	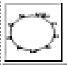	0.34	56.4	-30.8	-93.47	Ct Det
2	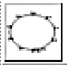	0.34	52.5	-14.9	-45.75	Ct Det
3	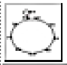	0.68	52.9	-31	-95.09	Ct Det
4	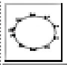	1.17	44.3	-23.8	-74.97	Ct Det

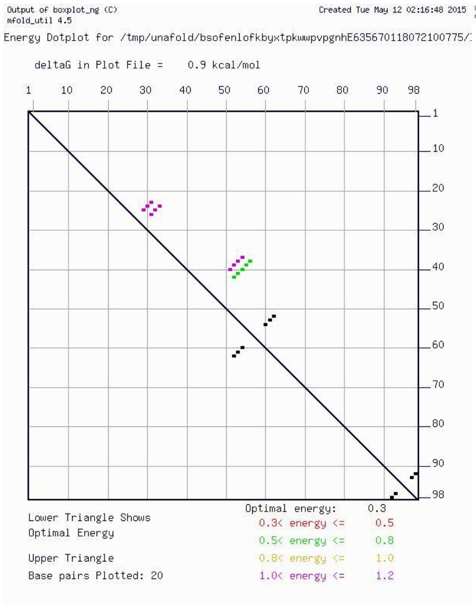

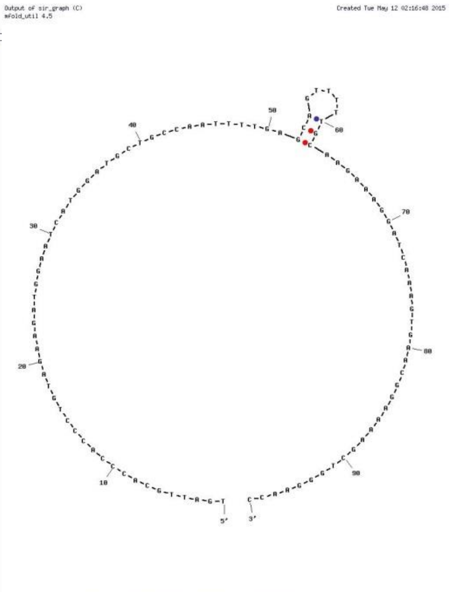

dG = 0.341 bsofenlofkbyxtpkwwpvpgnhE635670118072100775

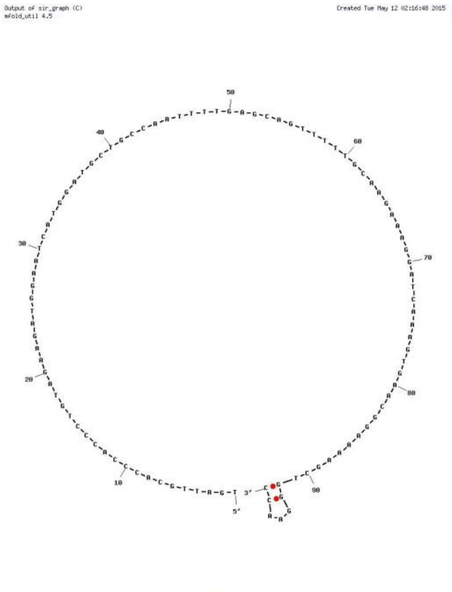

dG = 0.343 bsofenlofkbyxtpkwwpvpgnhE635670118072100775

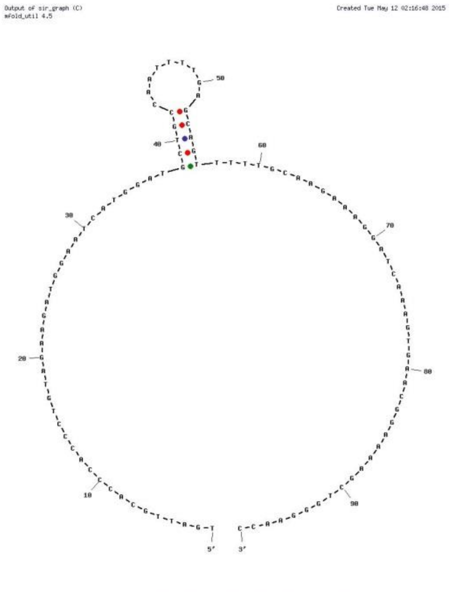

dG = 0.68 bsofenlofkbyxtpkwwpvpgnhE635670118072100775

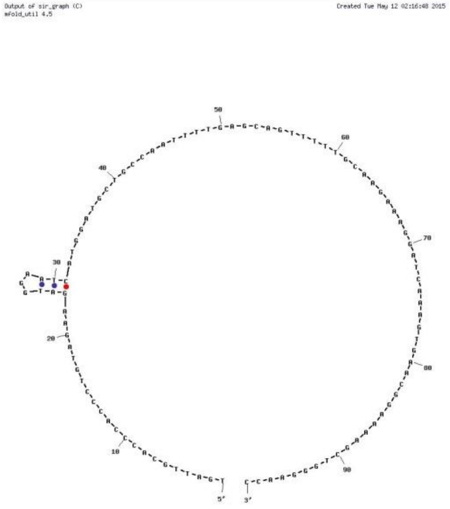

dG = 1.176 bsofenlofkbyxtpkwwpvpgnhE635670118072100775

RPL22 BeaconDesigner™ Free Edition (Premier BioSoft International, Palo Alto, CA, USA, http://www.premierbiosoft.com/qOligo/Oligo.jsp?PID=1)

Secondary Structures for Sense Primer

Dimer:-

```
        5' TGATTGCACCCACCCTGTAG 3'
            ¦¦  ||||  ¦¦
3' GATGTCCCACCCACGTTAGT 5'
                                                                       -3.4
```

Hairpin:-

Not Found

Secondary Structures for Anti-sense Primer

Dimer:-

```
  5' GGTTCCCAGCTTTTCCGTTC 3'
            ||||
3' CTTGCCTTTTCGACCCTTGG 5'
                                                                       -3.0
```

Hairpin:-

Not Found

Cross Dimer

Cross Dimer between Sense Primer and Anti-sense Primer:-

```
      5' TGATTGCACCCACCCTGTAG 3'
            ¦        |||
3' CTTGCCTTTTCGACCCTTGG 5'
                                                                       -1.5

          5' TGATTGCACCCACCCTGTAG 3'
                   ¦ |||
3' CTTGCCTTTTCGACCCTTGG 5'
                                                                       -1.5

5' TGATTGCACCCACCCTGTAG 3'
         ¦    ¦  |||
   3' CTTGCCTTTTCGACCCTTGG 5'
                                                                       -1.1
```

EEF1A1 PrimerBLAST (National Center for Biotechnology Information, Bethesda MD, USA, https://www.ncbi.nlm.nih.gov/tools/primer-blast)

Primer pair 1

	Sequence (5'->3')	Template strand	Length	Start	Stop	Tm	GC%	Self complementarity	Self 3' complementarity
**Forward primer**	CCTGCCTCTCCAGGATGTCTAC	Plus	22	804	825	64.13	59.09	5.00	2.00
**Reverse primer**	GGAGCAAAGGTGACCACCATAC	Minus	22	908	887	63.70	54.55	6.00	2.00
**Product length**	105								
**Total intron size**	87 (between pos. 13288764 and 13288676 on NT_025741.16)								

Products on intended target

>NM_001402.5 Homo sapiens eukaryotic translation elongation factor 1 alpha 1 (EEF1A1), mRNA

```
product length = 105
Forward primer  1     CCTGCCTCTCCAGGATGTCTAC  22
Template        804   ......................  825

Reverse primer  1     GGAGCAAAGGTGACCACCATAC  22
Template        908   ......................  887
```

Products on allowed transcript variants

>XM_011535514.1 PREDICTED: Homo sapiens eukaryotic translation elongation factor 1 alpha 1 (EEF1A1), transcript variant X1, mRNA

```
product length = 105
Forward primer  1      CCTGCCTCTCCAGGATGTCTAC  22
Template        1349   ......................  1370

Reverse primer  1      GGAGCAAAGGTGACCACCATAC  22
Template        1453   ......................  1432
```

EEF1A1 UCSC In-silico-PCR Genome Browser (Dec. 2013 GRCh38/hg38; UCSC Genes; Max. Product Size: 4000; Min. Perfect Match: 15; Min. Good Match: 15; Jim Kent, http://genome-mirror.genomedk.au.dk/cgi-bin/hgPcr)

UCSC In-Silico PCR

The sequences and coordinates shown below are from UCSC Genes, not from the genome assembly. The links lead to the Genome Browser at the position of the entire target sequence.

```
>uc003phi.3__EEF1A1:1733+1837 105bp CCTGCCTCTCCAGGATGTCTAC GGAGCAAAGGTGACCACCATAC
CCTGCCTCTCCAGGATGTCTACaaaattggtggtattggtactgttcctg
ttggccgagtggagactggtgttctcaaacccgGTATGGTGGTCACCTTT
GCTCC

>uc033djt.1__EEF1A1:794+898 105bp CCTGCCTCTCCAGGATGTCTAC GGAGCAAAGGTGACCACCATAC
CCTGCCTCTCCAGGATGTCTACaaaattggtggtattggtactgttcctg
ttggccgagtggagactggtgttctcaaacccgGTATGGTGGTCACCTTT
GCTCC

>uc003phj.3__EEF1A1:804+908 105bp CCTGCCTCTCCAGGATGTCTAC GGAGCAAAGGTGACCACCATAC
CCTGCCTCTCCAGGATGTCTACaaaattggtggtattggtactgttcctg
ttggccgagtggagactggtgttctcaaacccgGTATGGTGGTCACCTTT
GCTCC
```

EEF1A1 PrimerCheck (SpliceCenter der Genomics and Bioinformatics Group, LMP, CCR, NCI, http://projects.insilico.us/SpliceCenter/PrimerCheck.jsp)

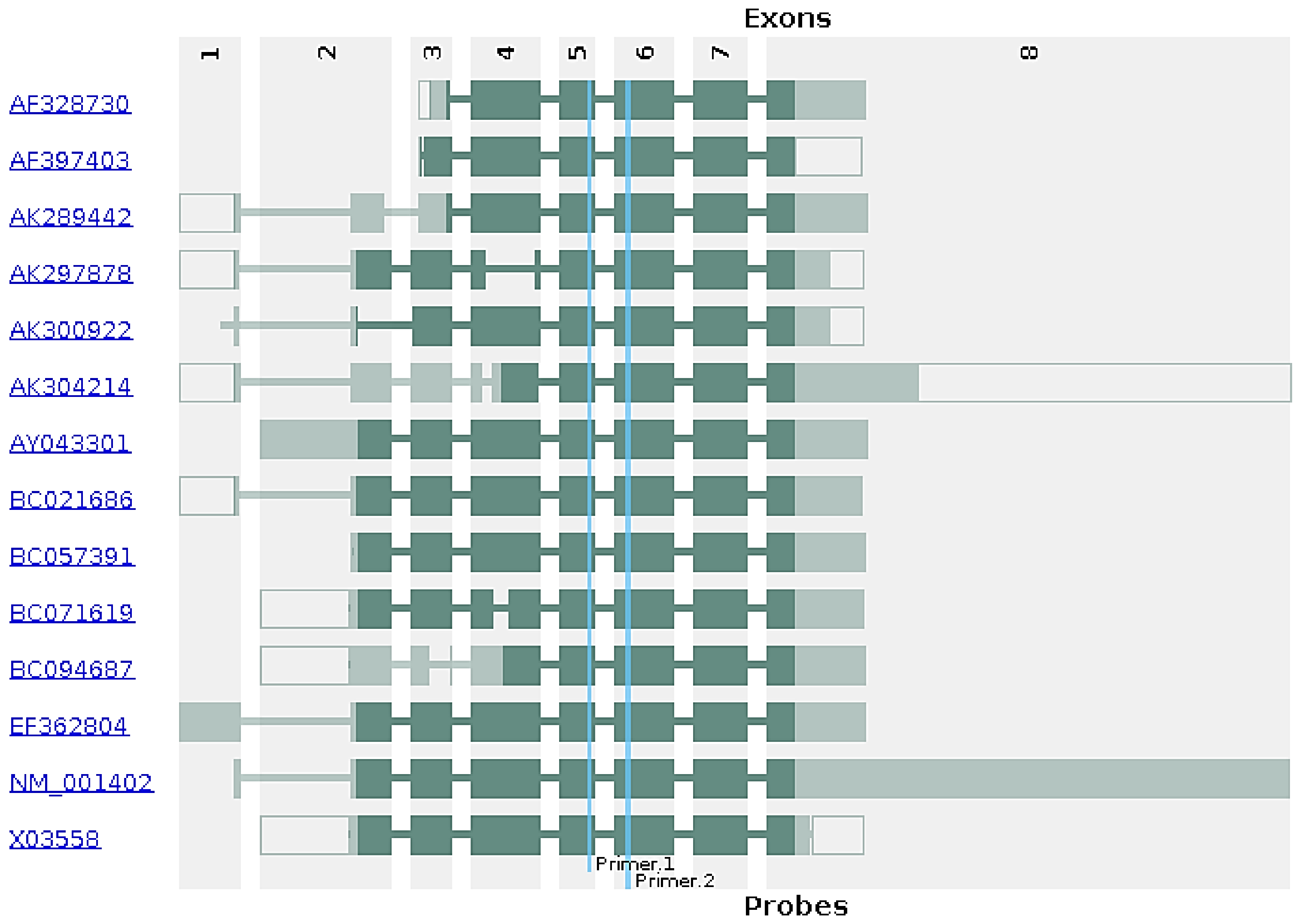

EEF1A1

EEF1A1 UNAFold (Integrated DNA Technologies Inc., Coralville, IA, USA, http://eu.idtdna.com/UNAFold? , Suboptimality 50%)

EEF1A1 Amplicon Sequence

5’ CCTGCCTCTCCAGGATGTCTACAAAATTGGTGGTATTGGTACTGTTCCTGTTGGCCGAGTGGAGACTGGTGTTCTCAAACCCGGTATGGTGGTCACCTTTGCTCC 3’

Structures

Structure Name	Image	ΔG (kcal.mole^{-1})	T_M (°C)	ΔH (kcal.mole^{-1})	ΔS (cal.K^{-1}mole^{-1})	Output
1	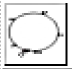	0.5	54.3	-28.9	-88.25	Ct Det
2	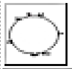	0.59	49.7	-18.5	-57.3	Ct Det
3	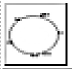	0.63	48.7	-18.1	-56.23	Ct Det
4	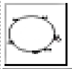	0.84	45.1	-18	-56.56	Ct Det
5	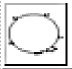	0.87	51.3	-32.5	-100.18	Ct Det
6	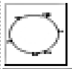	1.12	40.6	-18.2	-58.01	Ct Det
7	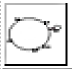	1.33	43.5	-25.4	-80.22	Ct Det
8	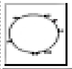	1.39	18.4	-9.7	-33.28	Ct Det
9	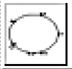	1.44	15.8	-9.4	-32.53	Ct Det

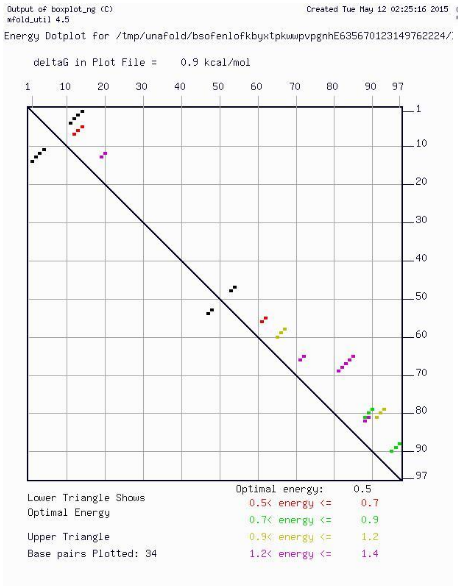
Output of boxplot_ng (C)
mfold_util 4.5
Created Tue May 12 02:25:16 2015
Energy Dotplot for /tmp/unafold/bsofenlofkbyxtpkwwpvpgnhE635670123149762224/
deltaG in Plot File = 0.9 kcal/mol
Lower Triangle Shows
Optimal Energy
Upper Triangle
Base pairs Plotted: 34
Optimal energy: 0.5
0.5< energy <= 0.7
0.7< energy <= 0.9
0.9< energy <= 1.2
1.2< energy <= 1.4

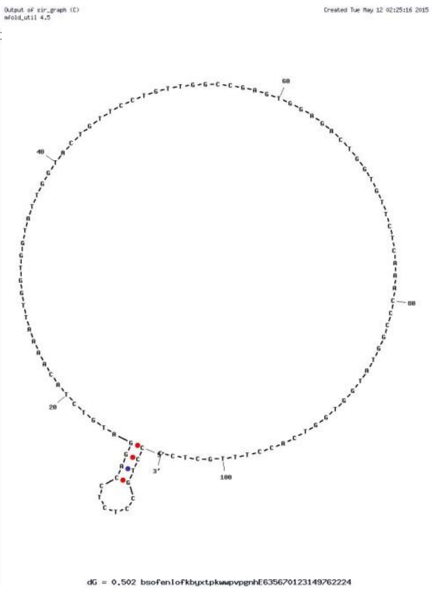
dG = 0.502 bsofenlofkbyxtpkwwpvpgnhE635670123149762224

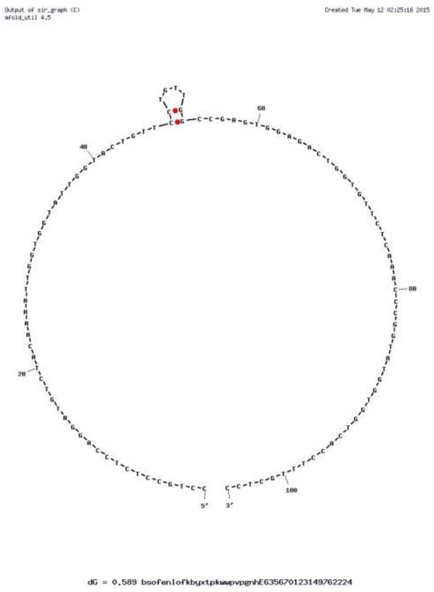
dG = 0.589 bsofenlofkbyxtpkwwpvpgnhE635670123149762224

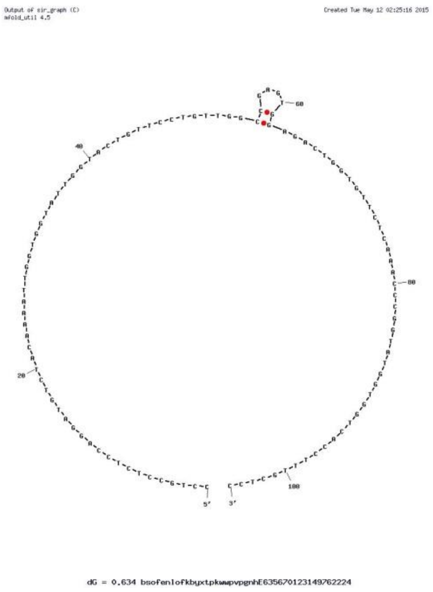
dG = 0.634 bsofenlofkbyxtpkwwpvpgnhE635670123149762224

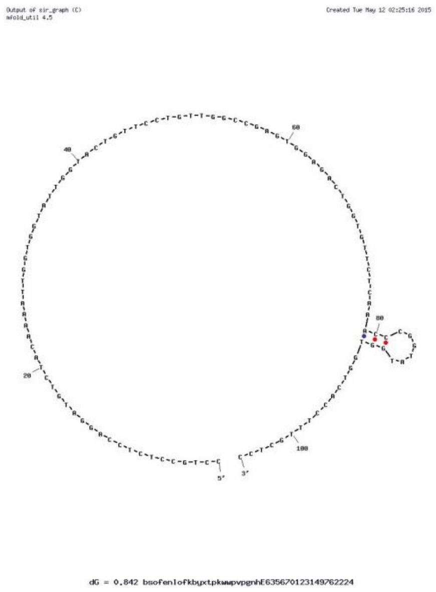
dG = 0.842 bsofenlofkbyxtpkwwpvpgnhE635670123149762224

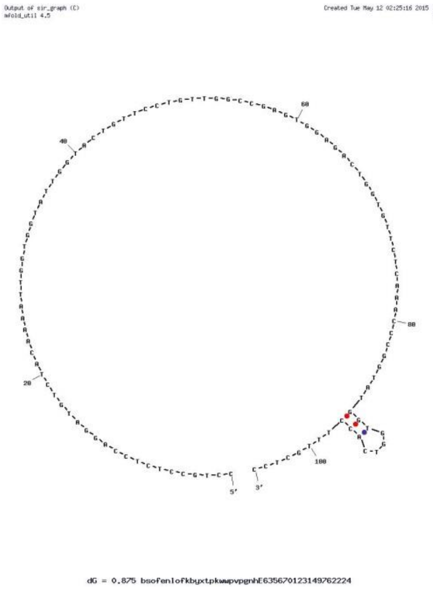
dG = 0.875 bsofenlofkbyxtpkwwpvpgnhE635670123149762224

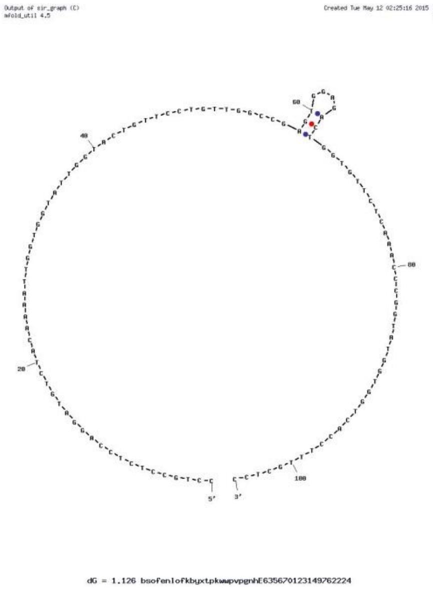
dG = 1.126 bsofenlofkbyxtpkwwpvpgnhE635670123149762224

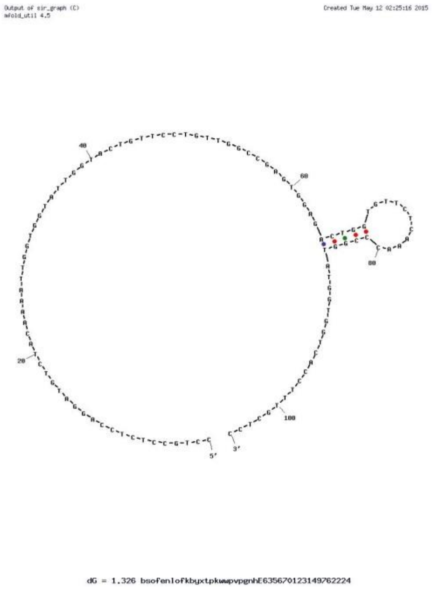
dG = 1.326 bsofenlofkbyxtpkwwpvpgnhE635670123149762224

Output of sir_graph (C)
mfold_util 4.5

Created Tue May 12 02:25:16 2015

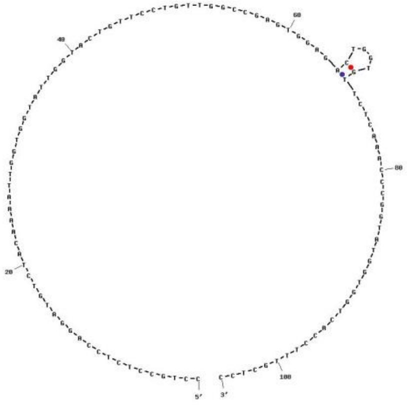

dG = 1.386 bsofenlofkbyxtpkwwpvpgnhE635670123149762224

Output of sir_graph (C)
mfold_util 4.5

Created Tue May 12 02:25:16 2015

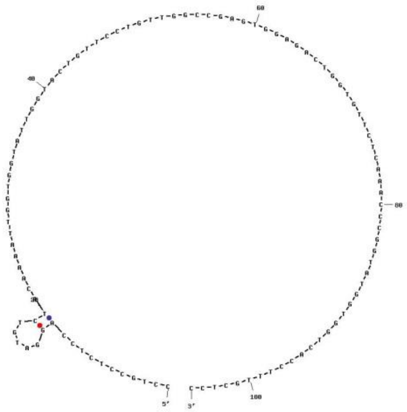

dG = 1.44 bsofenlofkbyxtpkwwpvpgnhE635670123149762224

EEF1A1 BeaconDesigner™ Free Edition (Premier BioSoft International, Palo Alto, CA, USA, http://www.premierbiosoft.com/qOligo/Oligo.jsp?PID=1)

Secondary Structures for Sense Primer

Dimer:-

```
        5' CCTGCCTCTCCAGGATGTCTAC 3'
              ||||      ¦¦¦¦
3' CATCTGTAGGACCTCTCCGTCC 5'
                                                                        -3.0

    5' CCTGCCTCTCCAGGATGTCTAC 3'
        ¦  |||    ¦¦¦  ¦
3' CATCTGTAGGACCTCTCCGTCC 5'
                                                                        -1.3

5' CCTGCCTCTCCAGGATGTCTAC 3'
     ¦     ||| ¦¦¦      ¦
 3' CATCTGTAGGACCTCTCCGTCC 5'
                                                                        -1.3
```

Hairpin:-

```
/TCCGTCC 5'
|   ||||
\CTCCAGGATGTCTAC 3'
                                                                        -3.0

/TCTCCGTCC 5'
|  |||  ¦
\CCAGGATGTCTAC 3'
                                                                        -1.3
```

Secondary Structures for Anti-sense Primer

Dimer:-

```
5' GGAGCAAAGGTGACCACCATAC 3'
         ¦ ||||  ¦¦¦¦ ¦
    3' CATACCACCAGTGGAAACGAGG 5'
                                                                        -3.2

5' GGAGCAAAGGTGACCACCATAC 3'
    ¦      ||| ¦¦¦       ¦
 3' CATACCACCAGTGGAAACGAGG 5'
                                                                        -1.5
```

Hairpin:-

```
/AGTGGAAACGAGG 5'
| ¦|||| ¦
\CCACCATAC 3'
                                                                        -1.5
```

Cross Dimer

Cross Dimer between Sense Primer and Anti-sense Primer:-

```
           5' CCTGCCTCTCCAGGATGTCTAC 3'
               ¦     ||||
3' CATACCACCAGTGGAAACGAGG 5'
                                                                        -2.9

              5' CCTGCCTCTCCAGGATGTCTAC 3'
                    |||  ¦
3' CATACCACCAGTGGAAACGAGG 5'
                                                                        -2.0

        5' CCTGCCTCTCCAGGATGTCTAC 3'
               ||| ¦ ¦
3' CATACCACCAGTGGAAACGAGG 5'
                                                                        -1.3

            5' CCTGCCTCTCCAGGATGTCTAC 3'
               |||      ¦
3' CATACCACCAGTGGAAACGAGG 5'
                                                                        -1.3

5' CCTGCCTCTCCAGGATGTCTAC 3'
               ¦¦  |||   ¦
        3' CATACCACCAGTGGAAACGAGG 5'
                                                                        -1.1

             5' CCTGCCTCTCCAGGATGTCTAC 3'
                ¦ ¦  |||
3' CATACCACCAGTGGAAACGAGG 5'
                                                                        -1.0

5' CCTGCCTCTCCAGGATGTCTAC 3'
               ¦ |||
            3' CATACCACCAGTGGAAACGAGG 5'
                                                                        -0.5
```

RPLP0 PrimerBLAST (National Center for Biotechnology Information, Bethesda MD, USA, https://www.ncbi.nlm.nih.gov/tools/primer-blast)

Primer pair 1

	Sequence (5'->3')	Template strand	Length	Start	Stop	Tm	GC%	Self complementarity	Self 3' complementarity
**Forward primer**	GAAACTCTGCATTCTCGCTTCC	Plus	22	802	823	62.34	50.00	4.00	0.00
**Reverse primer**	GACTCGTTTGTACCCGTTGATG	Minus	22	921	900	62.01	50.00	4.00	0.00
**Product length**	120								
**Total intron size**	1091 (between pos. 82963302 and 82962210 on NT_029419.13)								

Products on intended target

```
>NM_001002.3 Homo sapiens ribosomal protein, large, P0 (RPLP0), transcript variant 1, mRNA
product length = 120
Forward primer  1     GAAACTCTGCATTCTCGCTTCC   22
Template        802   ......................   823

Reverse primer  1     GACTCGTTTGTACCCGTTGATG   22
Template        921   ......................   900
```

Products on allowed transcript variants

```
>NM_053275.3 Homo sapiens ribosomal protein, large, P0 (RPLP0), transcript variant 2, mRNA
product length = 120
Forward primer  1     GAAACTCTGCATTCTCGCTTCC   22
Template        862   ......................   883

Reverse primer  1     GACTCGTTTGTACCCGTTGATG   22
Template        981   ......................   960
```

RPLP0 UCSC In-silico-PCR Genome Browser (Dec. 2013 GRCh38/hg38; UCSC Genes; Max. Product Size: 4000; Min. Perfect Match: 15; Min. Good Match: 15; Jim Kent, http://genome-mirror.genomedk.au.dk/cgi-bin/hgPcr).

UCSC In-Silico PCR

The sequences and coordinates shown below are from UCSC Genes, not from the genome assembly. The links lead to the Genome Browser at the position of the entire target sequence.

```
>uc001txp.3__RPLP0:862+981 120bp GAAACTCTGCATTCTCGCTTCC GACTCGTTTGTACCCGTTGATG
GAAACTCTGCATTCTCGCTTCCtggagggtgtccgcaatgttgccagtgt
ctgtctgcagattggctacccaactgttgcatcagtaccccattctatCA
TCAACGGGTACAAACGAGTC

>uc001txq.3__RPLP0:802+921 120bp GAAACTCTGCATTCTCGCTTCC GACTCGTTTGTACCCGTTGATG
GAAACTCTGCATTCTCGCTTCCtggagggtgtccgcaatgttgccagtgt
ctgtctgcagattggctacccaactgttgcatcagtaccccattctatCA
TCAACGGGTACAAACGAGTC
```

RPLP0 PrimerCheck (SpliceCenter der Genomics and Bioinformatics Group, LMP, CCR, NCI, http://projects.insilico.us/SpliceCenter/PrimerCheck.jsp)

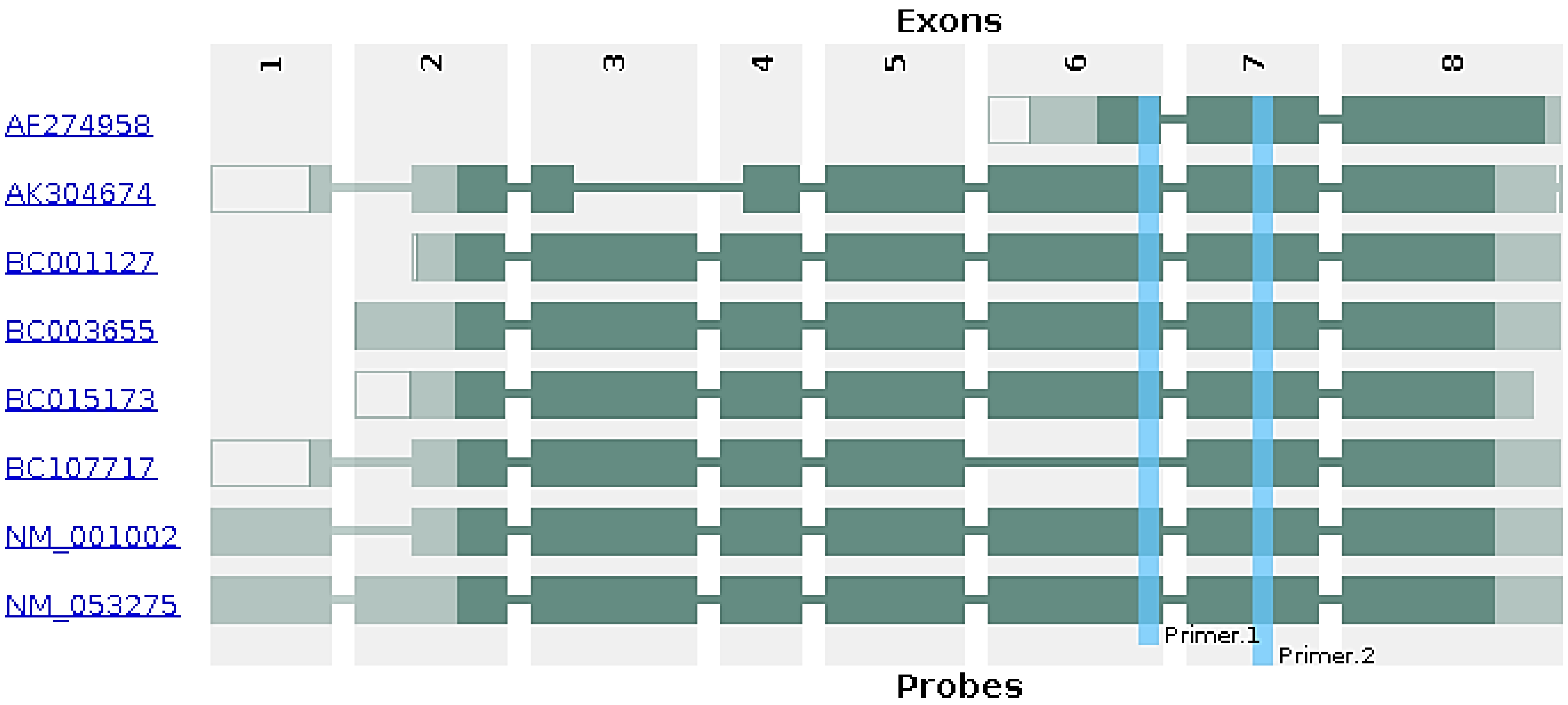

RPLP0

RPLP0 UNAFold (Integrated DNA Technologies Inc., Coralville, IA, USA, http://eu.idtdna.com/UNAFold? , Suboptimality 50%)

RPLP0 Amplicon Sequence

5’ GAAACTCTGCATTCTCGCTTCCTGGAGGGTGTCCGCAATGTTGCCAGTGTCTGTCTGCAGATTGGCTACCCAACTGTTGCATCAGTACCCCATTCTATCATCAACGGGTACAAACGAGTC 3’

Structures

Structure Name	Image	ΔG (kcal.mole^{-1})	T_M (^oC)	ΔH (kcal.mole^{-1})	ΔS (cal.K^{-1}mole^{-1})	Output
1	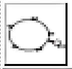	0.15	59	-51	-153.54	Ct Det
2	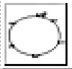	0.67	52.1	-27.6	-84.86	Ct Det
3	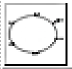	0.69	52.3	-29.1	-89.41	Ct Det
4	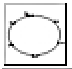	0.76	50.8	-26.9	-83.03	Ct Det
5	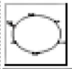	0.85	49.4	-25.9	-80.29	Ct Det
6	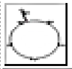	0.95	55.1	-63.5	-193.46	Ct Det
7	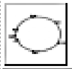	1.12	42.1	-19.7	-62.49	Ct Det

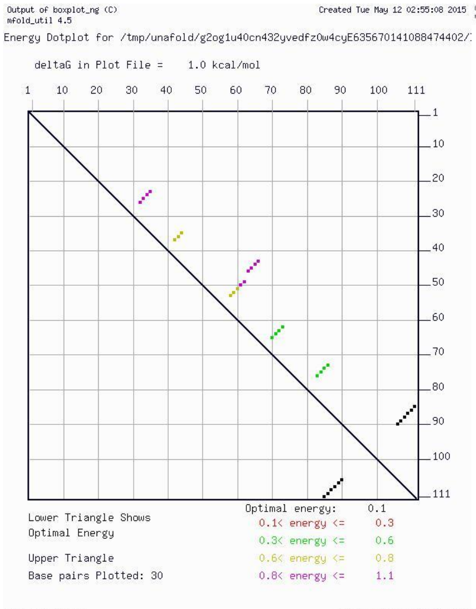
Output of boxplot_ng (C)
mfold_util 4.5
Created Tue May 12 02:55:08 2015
Energy Dotplot for /tmp/unafold/g2og1u40cn432yvedfz0w4cyE635670141088474402/
deltaG in Plot File = 1.0 kcal/mol
Lower Triangle Shows
Optimal Energy
Upper Triangle
Base pairs Plotted: 30
Optimal energy: 0.1
0.1< energy <= 0.3
0.3< energy <= 0.6
0.6< energy <= 0.8
0.8< energy <= 1.1

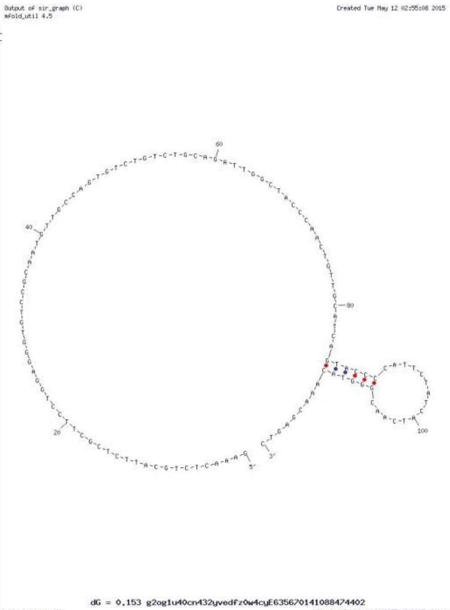
dG = 0.153 g2og1u40cn432yvedfz0w4cyE635670141088474402

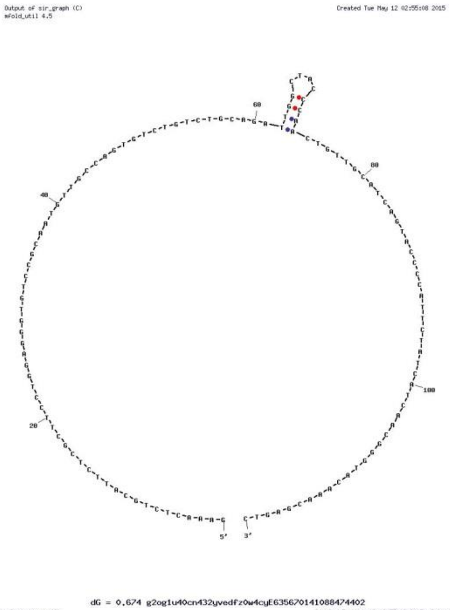
dG = 0.674 g2og1u40cn432yvedfz0w4cyE635670141088474402

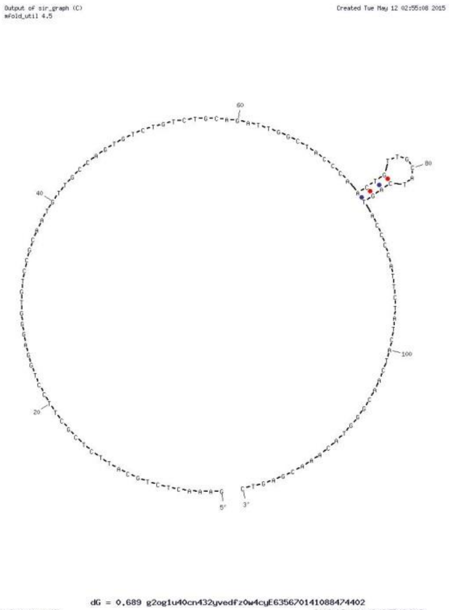
dG = 0.689 g2og1u40cn432yvedfz0w4cyE635670141088474402

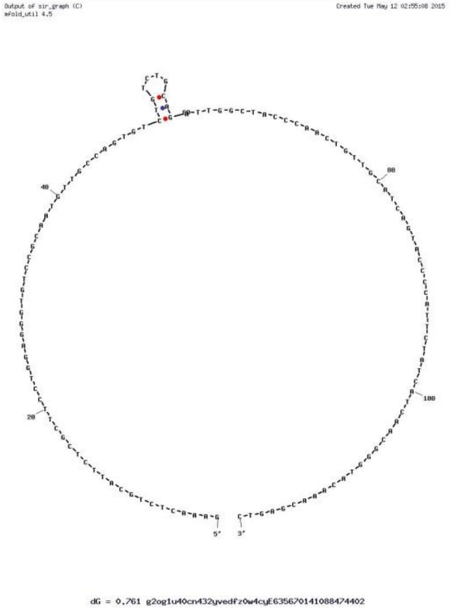
dG = 0.761 g2og1u40cn432yvedfz0w4cyE635670141088474402

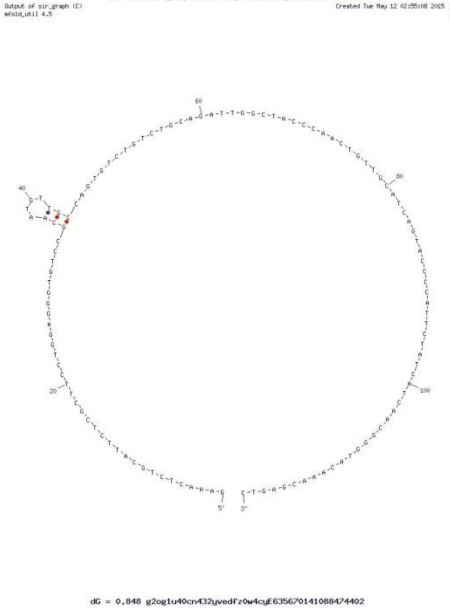
dG = 0.848 g2og1u40cn432yvedfz0w4cyE635670141088474402

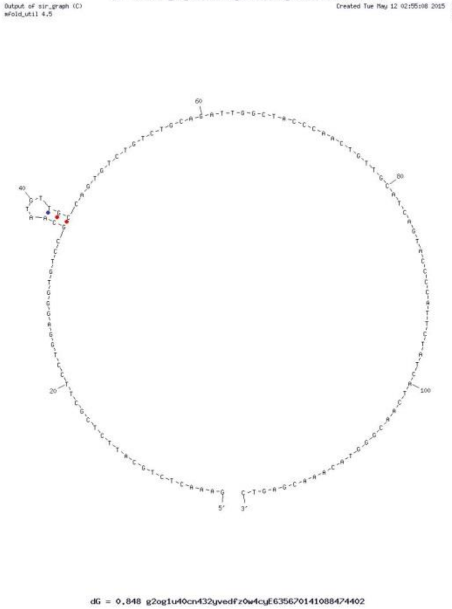
dG = 0.848 g2og1u40cn432yvedfz0w4cyE635670141088474402

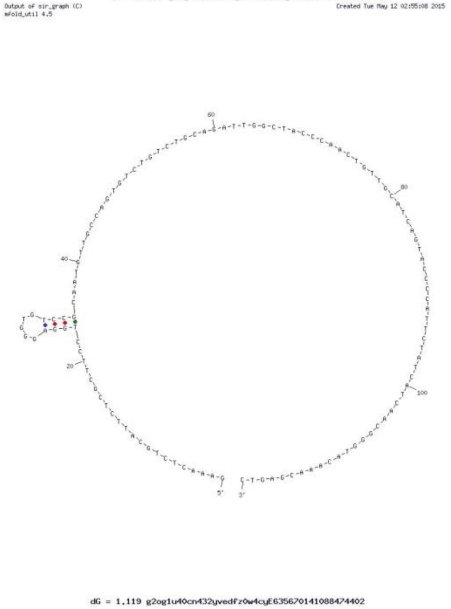
dG = 1.119 g2og1u40cn432yvedfz0w4cyE635670141088474402

RPLP0 BeaconDesigner™ Free Edition (Premier BioSoft International, Palo Alto, CA, USA, http://www.premierbiosoft.com/qOligo/Oligo.jsp?PID=1)

Secondary Structures for Sense Primer

Dimer:-

```
    5' GAAACTCTGCATTCTCGCTTCC 3'
       ¦  ¦   ||||   ¦  ¦
3' CCTTCGCTCTTACGTCTCAAAG 5'
                                                          -3.4

 5' GAAACTCTGCATTCTCGCTTCC 3'
    ||| ¦           ¦ ¦¦¦
3' CCTTCGCTCTTACGTCTCAAAG 5'
                                                          -0.6

        5' GAAACTCTGCATTCTCGCTTCC 3'
           |||         ¦¦¦
3' CCTTCGCTCTTACGTCTCAAAG 5'
                                                          -0.6
```

Hairpin:-

```
/CGTCTCAAAG 5'
A     ¦ |||
\TTCTCGCTTCC 3'
                                                          -0.6

/CTCAAAG 5'
|    |||
\TGCATTCTCGCTTCC 3'
                                                          -0.6
```

Secondary Structures for Anti-sense Primer

Dimer:-

```
5' GACTCGTTTGTACCCGTTGATG 3'
    ¦       ||||       ¦
3' GTAGTTGCCCATGTTTGCTCAG 5'
                                                          -2.0
```

Hairpin:-

Not Found

Cross Dimer

Cross Dimer between Sense Primer and Anti-sense Primer:-

```
            5' GAAACTCTGCATTCTCGCTTCC 3'
                ||||    ¦
3' GTAGTTGCCCATGTTTGCTCAG 5'
                                                                             -1.8

  5' GAAACTCTGCATTCTCGCTTCC 3'
       |||             ¦
3' GTAGTTGCCCATGTTTGCTCAG 5'
                                                                             -0.7

5' GAAACTCTGCATTCTCGCTTCC 3'
            |||   ¦¦  ¦ ¦
         3' GTAGTTGCCCATGTTTGCTCAG 5'
                                                                             -0.5
```

RNA18S5 PrimerBLAST (National Center for Biotechnology Information, Bethesda MD, USA, https://www.ncbi.nlm.nih.gov/tools/primer-blast)

Primer pair 1

	Sequence (5'->3')	Template strand	Length	Start	Stop	Tm	GC%	Self complementarity	Self 3' complementarity
**Forward primer**	AACTGCGAATGGCTCATTAAATC	Plus	23	84	106	60.55	39.13	6.00	3.00
**Reverse primer**	GCCCGTCGGCATGTATTAG	Minus	19	186	168	60.86	57.89	5.00	1.00
**Product length**	103								

RNA18S5 UCSC In-silico-PCR Genome Browser (Dec. 2013 GRCh38/hg38; UCSC Genes; Max. Product Size: 4000; Min. Perfect Match: 15; Min. Good Match: 15; Jim Kent, http://genome-mirror.genomedk.au.dk/cgi-bin/hgPcr).

UCSC In-Silico PCR

The sequences and coordinates shown below are from UCSC Genes, not from the genome assembly. The links lead to the Genome Browser at the position of the entire target sequence.

```
>uc032qts.1__RNA45S5:3742+3844 103bp AACTGCGAATGGCTCATTAAATC GCCCGTCGGCATGTATTAG
AACTGCGAATGGCTCATTAAATCagttatggttcctttggtcgctcgctc
ctctcctacttggataactgtggtaattctagagCTAATACATGCCGACG
GGC

>uc032ptn.1__RNA18S5:84+186 103bp AACTGCGAATGGCTCATTAAATC GCCCGTCGGCATGTATTAG
AACTGCGAATGGCTCATTAAATCagttatggttcctttggtcgctcgctc
ctctcctacttggataactgtggtaattctagagCTAATACATGCCGACG
GGC

>uc032ptu.1__RNA45S5:3738+3840 103bp AACTGCGAATGGCTCATTAAATC GCCCGTCGGCATGTATTAG
AACTGCGAATGGCTCATTAAATCagttatggttcctttggtcgctcgctc
ctctcctacttggataactgtggtaattctagagCTAATACATGCCGACG
GGC

>uc033dni.1__RNA18S5:84+186 103bp AACTGCGAATGGCTCATTAAATC GCCCGTCGGCATGTATTAG
AACTGCGAATGGCTCATTAAATCagttatggttcctttggtcgctcgctc
ctctcctacttggataactgtggtaattctagagCTAATACATGCCGACG
GGC

>uc032pst.1__RNA18S5:84+186 103bp AACTGCGAATGGCTCATTAAATC GCCCGTCGGCATGTATTAG
AACTGCGAATGGCTCATTAAATCagttatggttcctttggtcgctcgctc
ctctcctacttggataactgtggtaattctagagCTAATACATGCCGACG
GGC

>uc032qty.1__RNA18S5:84+186 103bp AACTGCGAATGGCTCATTAAATC GCCCGTCGGCATGTATTAG
AACTGCGAATGGCTCATTAAATCagttatggttcctttggtcgctcgctc
ctctcctacttggataactgtggtaattctagagCTAATACATGCCGACG
GGC

>uc033dng.1__RNA45S5:3738+3840 103bp AACTGCGAATGGCTCATTAAATC GCCCGTCGGCATGTATTAG
AACTGCGAATGGCTCATTAAATCagttatggttcctttggtcgctcgctc
ctctcctacttggataactgtggtaattctagagCTAATACATGCCGACG
GGC
```

RNA18S5 UNAFold (Integrated DNA Technologies Inc., Coralville, IA, USA, http://eu.idtdna.com/UNAFold? , Suboptimality 50%)

RNA18S5 Amplicon Sequence

5’ AACTGCGAATGGCTCATTAAATCAGTTATGGTTCCTTTGGTCGCTCGCTCCTCTCCTACTTGGATAACTGTGGTAATTCTAGAGCTAATACATGCCGACGGGC 3’

Structures

Structure Name	Image	ΔG (kcal.mole^{-1})	T_M (^oC)	ΔH (kcal.mole^{-1})	ΔS (cal.K^{-1}mole^{-1})	Output
1	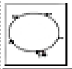	0.08	58.9	-25.6	-77.09	Ct Det
2	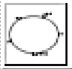	0.77	47.2	-19.4	-60.55	Ct Det
3	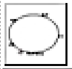	0.99	46.1	-22.8	-71.42	Ct Det

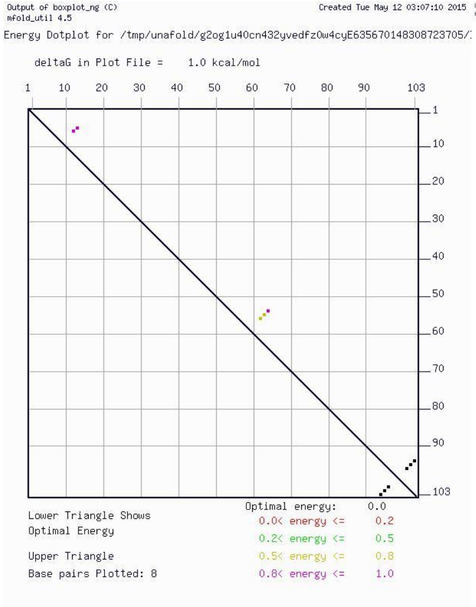

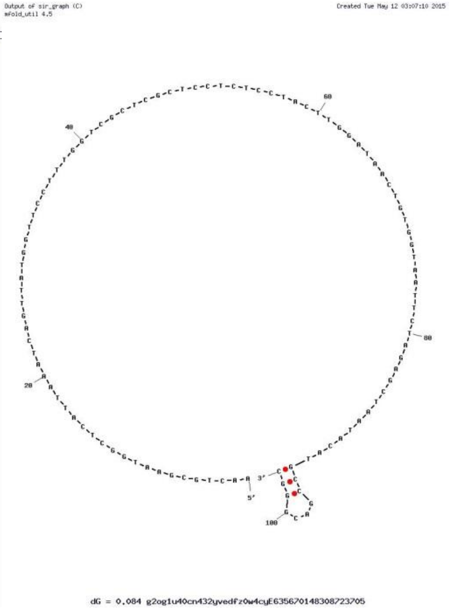

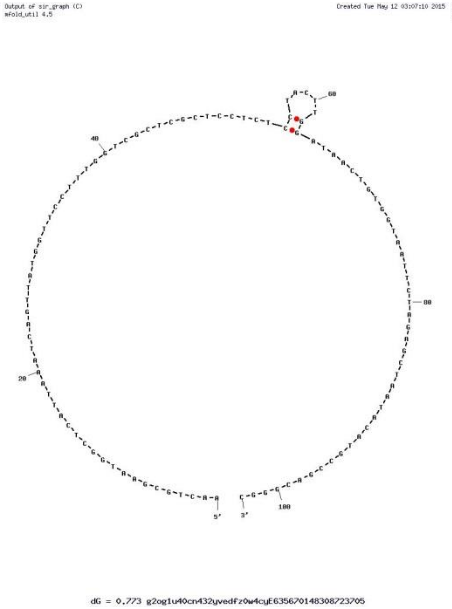

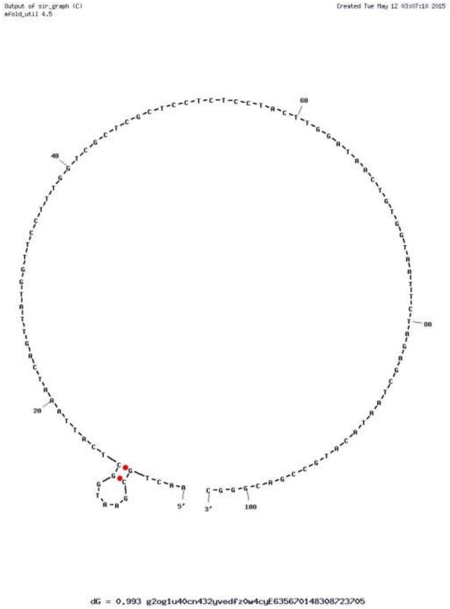

RNA18S5 BeaconDesigner™ Free Edition (Premier BioSoft International, Palo Alto, USA, http://www.premierbiosoft.com/qOligo/Oligo.jsp?PID=1)

Secondary Structures for Sense Primer

Dimer:-

```
5' AACTGCGAATGGCTCATTAAATC 3'
          ||||   ¦¦¦¦
  3' CTAAATTACTCGGTAAGCGTCAA 5'
                                        -1.7

5' AACTGCGAATGGCTCATTAAATC 3'
                   ||||
             3' CTAAATTACTCGGTAAGCGTCAA 5'
                                        -0.8

5' AACTGCGAATGGCTCATTAAATC 3'
                  ||| ¦¦¦
              3' CTAAATTACTCGGTAAGCGTCAA 5'
                                        0.0
```

Hairpin:-

```
/GGTAAGCGTCAA 5'
C ||||
\TCATTAAATC 3'
                                        -1.7
```

Secondary Structures for Anti-sense Primer

Dimer:-

```
        5' GCCCGTCGGCATGTATTAG 3'
                |||  ¦¦¦
3' GATTATGTACGGCTGCCCG 5'
                                        -2.4

         5' GCCCGTCGGCATGTATTAG 3'
              |||     ¦¦¦
3' GATTATGTACGGCTGCCCG 5'
                                        -2.4

5' GCCCGTCGGCATGTATTAG 3'
      ¦     ||||     ¦
   3' GATTATGTACGGCTGCCCG 5'
                                        -2.3
```

Hairpin:-

```
/GCCCG 5'
|  |||
\TCGGCATGTATTAG 3'
                                        -2.4
```

Cross Dimer

Cross Dimer between Sense Primer and Anti-sense Primer:-

```
     5' AACTGCGAATGGCTCATTAAATC 3'
           ¦   ¦¦¦   |||
3' GATTATGTACGGCTGCCCG 5'
                                        -2.4

    5' AACTGCGAATGGCTCATTAAATC 3'
         ¦  |||  ¦ ¦¦
3' GATTATGTACGGCTGCCCG 5'
                                        -2.0

5' AACTGCGAATGGCTCATTAAATC 3'
             ¦    |||      ¦
       3' GATTATGTACGGCTGCCCG 5'
                                        -0.5

5' AACTGCGAATGGCTCATTAAATC 3'
            ||| ¦
 3' GATTATGTACGGCTGCCCG 5'
                                        -0.5

5' AACTGCGAATGGCTCATTAAATC 3'
                      |||
               3' GATTATGTACGGCTGCCCG 5'
                                        0.0

5' AACTGCGAATGGCTCATTAAATC 3'
         ¦ |||    ¦    ¦
    3' GATTATGTACGGCTGCCCG 5'
                                        0.0

5' AACTGCGAATGGCTCATTAAATC 3'
                  |||  ¦
              3' GATTATGTACGGCTGCCCG 5'
                                        0.3
```

Supplementary Data 2. RNA integrity analysis. Experimental groups: K7-K12 = control; D7-D12 = compressive orthodontic force; Agac7-Agac12 = bacterial lysate (periodontitis).

Assay Class:	Eukaryote Total RNA Nano	Created:	08.03.2017 10:01:40
Data Path:	E:\...Eukaryote Total RNA Nano_DE72901710_2017-03-08_10-01-40.xad	Modified:	08.03.2017 10:25:31

Electrophoresis File Run Summary

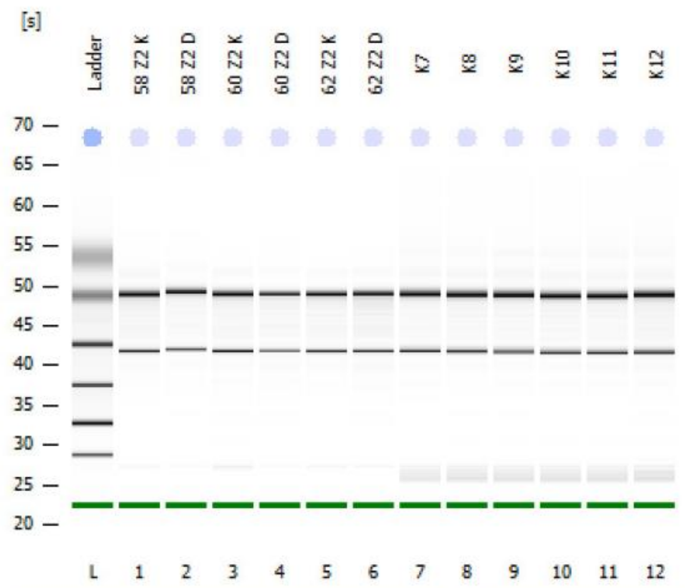

Instrument Information:

Instrument Name:	DE72901710	Firmware:	C.01.069
Serial#:	DE72901710	Type:	G2939A

Assay Information:

Assay Origin Path:	C:\Programme\Agilent\2100 bioanalyzer\2100 expert\assays\RNA\Eukaryote Total RNA Nano Series II.xsy
Assay Class:	Eukaryote Total RNA Nano
Version:	2.6
Assay Comments:	Total RNA Analysis ng sensitivity (Eukaryote)

© Copyright 2003 - 2009 Agilent Technologies, Inc.

Chip Information:

Chip Lot #:

Reagent Kit Lot #:

Chip Comments:

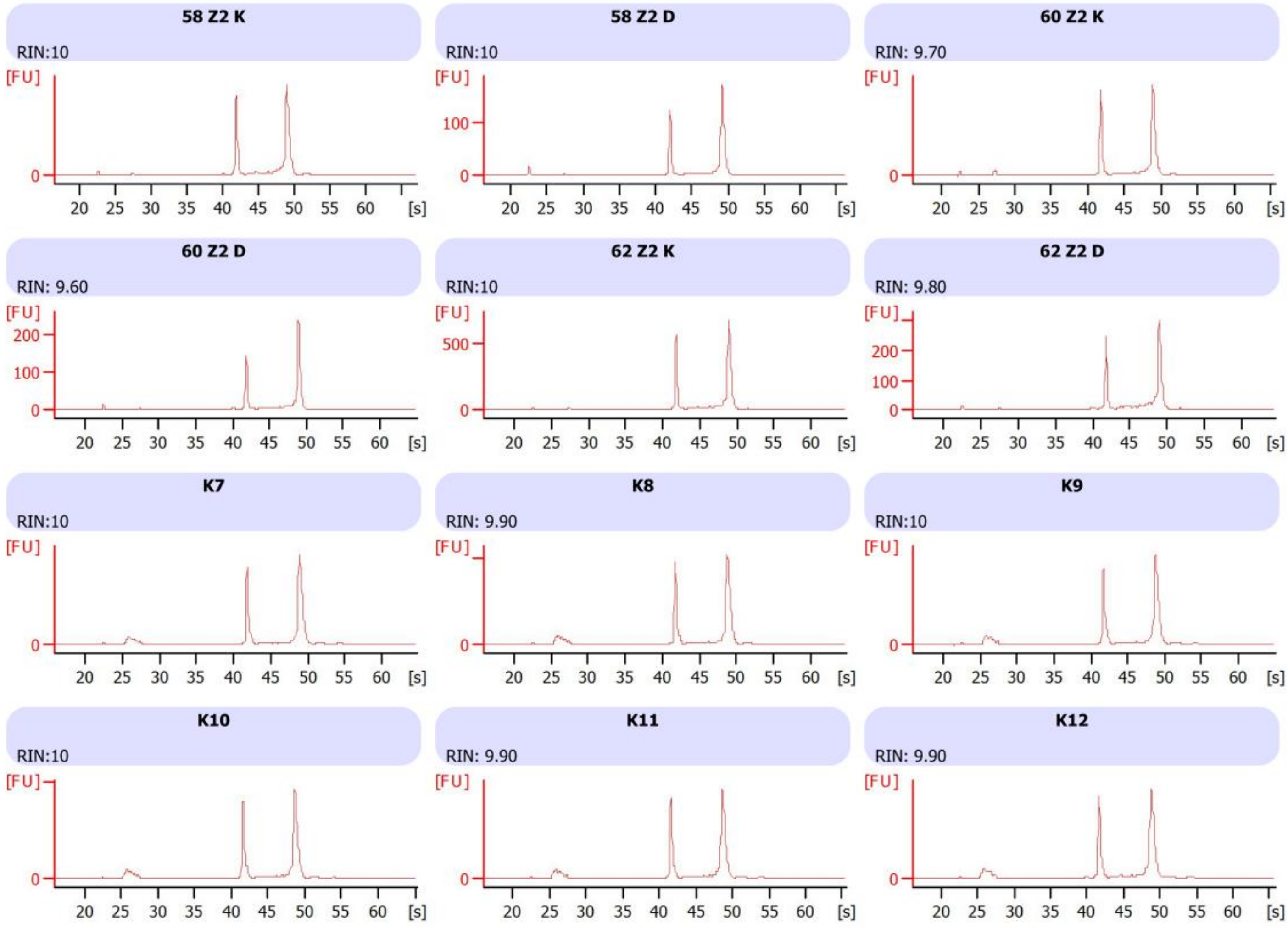

Assay Class: Eukaryote Total RNA Nano
Data Path: E:\...Eukaryote Total RNA Nano_DE72901710_2017-03-08_10-01-40.xad
Created: 08.03.2017 10:01:40
Modified: 08.03.2017 10:25:31

Electrophoresis File Run Summary (Chip Summary)

Sample Name	Sample Comment	Status	Result Label	Result Color
58 Z2 K		✔	RIN:10	
58 Z2 D		✔	RIN:10	
60 Z2 K		✔	RIN: 9.70	
60 Z2 D		✔	RIN: 9.60	
62 Z2 K		✔	RIN:10	
62 Z2 D		✔	RIN: 9.80	
K7		✔	RIN:10	
K8		✔	RIN: 9.90	
K9		✔	RIN:10	
K10		✔	RIN:10	
K11		✔	RIN: 9.90	
K12		✔	RIN: 9.90	
Ladder		✔	All Other Samples	

Chip Lot # **Reagent Kit Lot #**

Chip Comments :

© Copyright 2003 - 2009 Agilent Technologies, Inc.

Assay Class: Eukaryote Total RNA Nano Created: 08.03.2017 10:01:40
Data Path: E:\...Eukaryote Total RNA Nano_DE72901710_2017-03-08_10-01-40.xad Modified: 08.03.2017 10:25:31

Electrophoresis Assay Details

General Analysis Settings

Number of Available Sample and Ladder Wells (Max.) : 13
Minimum Visible Range [s] : 17
Maximum Visible Range [s] : 70
Start Analysis Time Range [s] : 19
End Analysis Time Range [s] : 69
Ladder Concentration [ng/µl] : 150
Lower Marker Concentration [ng/µl] : 0
Upper Marker Concentration [ng/µl] : 0
Used Lower Marker for Quantitation
Standard Curve Fit is Logarithmic
Show Data Aligned to Lower Marker

Integrator Settings

Integration Start Time [s] : 19
Integration End Time [s] : 69
Slope Threshold : 0,6
Height Threshold [FU] : 0,5
Area Threshold : 0,2
Width Threshold [s] : 0,5
Baseline Plateau [s] : 6

Filter Settings

Filter Width [s] : 0,5
Polynomial Order : 4

Ladder

Ladder Peak	Size
1	25
2	200
3	500
4	1000
5	2000
6	4000

© Copyright 2003 - 2009 Agilent Technologies, Inc.

Assay Class: Eukaryote Total RNA Nano
Data Path: E:\...Eukaryote Total RNA Nano_DE72901710_2017-03-08_10-01-40.xad
Created: 08.03.2017 10:01:40
Modified: 08.03.2017 10:25:31

Electropherogram Summary Continued ...

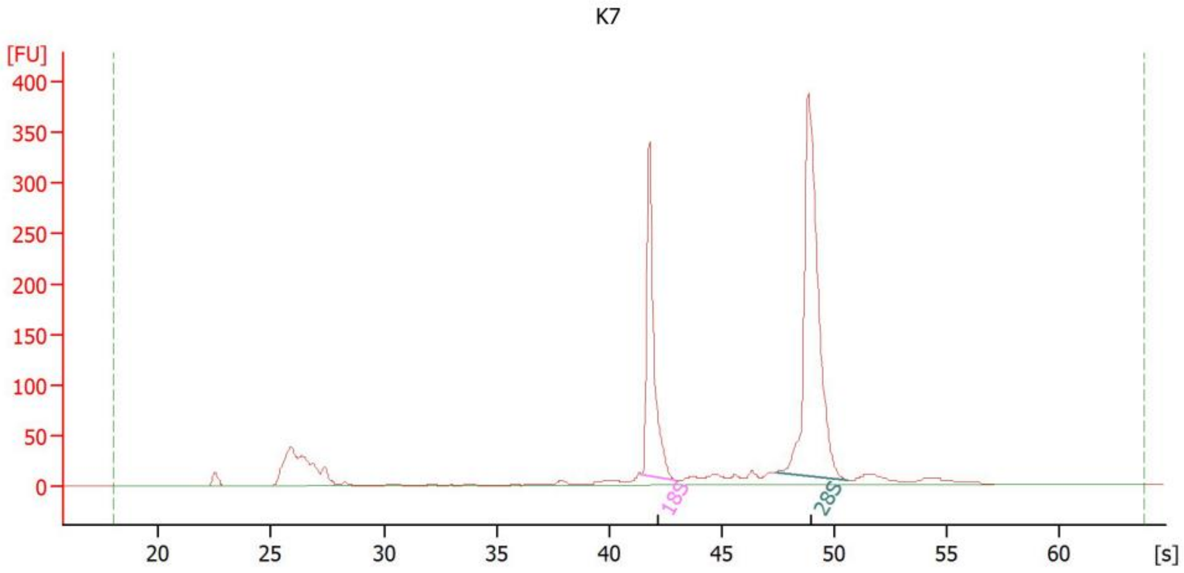

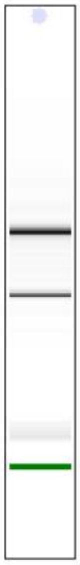

Overall Results for sample 7 : K7

RNA Area:	1.347,6	RNA Integrity Number (RIN):	10 (B.02.08)
RNA Concentration:	471 ng/µl	Result Flagging Color:	
rRNA Ratio [28s / 18s]:	1,8	Result Flagging Label:	RIN:10

Fragment table for sample 7 : K7

Name	Start Time [s]	End Time [s]	Area	% of total Area
18S	41,44	42,97	298,3	22,1
28S	47,40	50,59	547,4	40,6

 © Copyright 2003 - 2009 Agilent Technologies, Inc.

Assay Class: Eukaryote Total RNA Nano
Data Path: E:\...Eukaryote Total RNA Nano_DE72901710_2017-03-08_10-01-40.xad
Created: 08.03.2017 10:01:40
Modified: 08.03.2017 10:25:31

Electropherogram Summary Continued ...

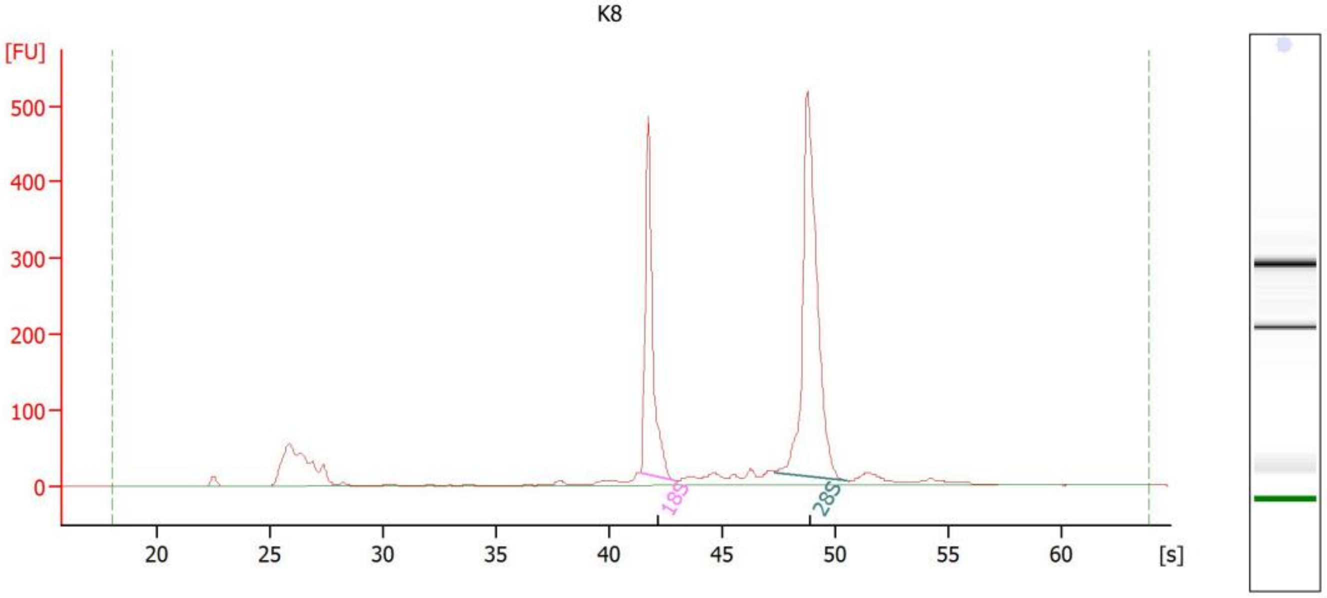

Overall Results for sample 8 : K8

RNA Area:	1.892,6	RNA Integrity Number (RIN):	9.9 (B.02.08)
RNA Concentration:	661 ng/µl	Result Flagging Color:	
rRNA Ratio [28s / 18s]:	1,8	Result Flagging Label:	RIN: 9.90

Fragment table for sample 8 : K8

Name	Start Time [s]	End Time [s]	Area	% of total Area
18S	41,39	42,92	424,1	22,4
28S	47,31	50,51	757,6	40,0

© Copyright 2003 - 2009 Agilent Technologies, Inc.

Assay Class:	Eukaryote Total RNA Nano	Created:	08.03.2017 10:01:40
Data Path:	E:\...Eukaryote Total RNA Nano_DE72901710_2017-03-08_10-01-40.xad	Modified:	08.03.2017 10:25:31

Electropherogram Summary Continued ...

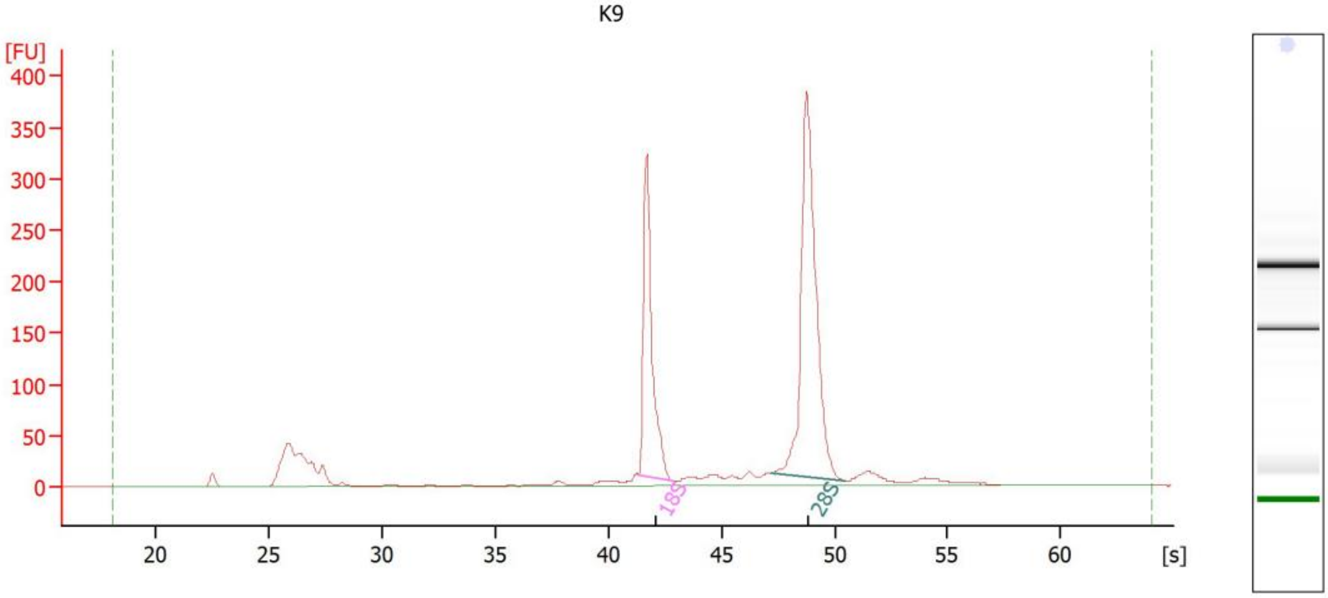

Overall Results for sample 9 : K9

RNA Area:	1.413,9	RNA Integrity Number (RIN):	10 (B.02.08)
RNA Concentration:	494 ng/µl	Result Flagging Color:	
rRNA Ratio [28s / 18s]:	1,8	Result Flagging Label:	RIN:10

Fragment table for sample 9 : K9

Name	Start Time [s]	End Time [s]	Area	% of total Area
18S	41,34	42,87	312,2	22,1
28S	47,23	50,47	568,9	40,2

Assay Class:	Eukaryote Total RNA Nano	Created:	08.03.2017 10:01:40
Data Path:	E:\...Eukaryote Total RNA Nano_DE72901710_2017-03-08_10-01-40.xad	Modified:	08.03.2017 10:25:31

Electropherogram Summary Continued ...

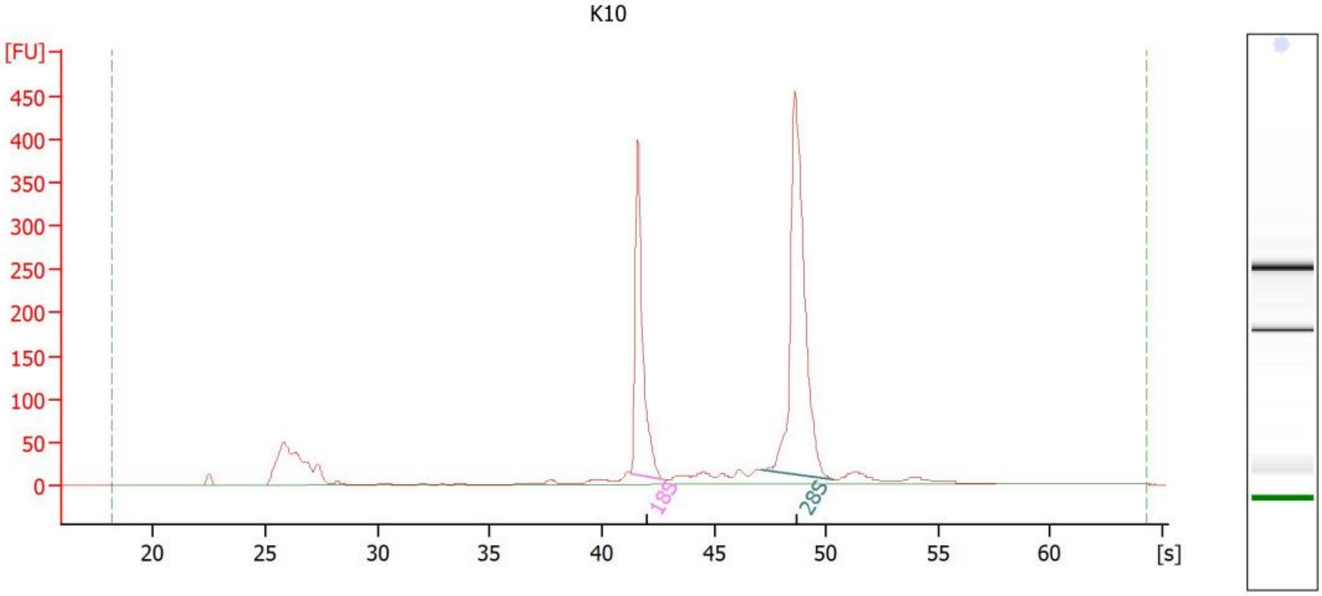

Overall Results for sample 10 : K10

RNA Area:	1.661,9	RNA Integrity Number (RIN):	10 (B.02.08)
RNA Concentration:	581 ng/µl	Result Flagging Color:	
rRNA Ratio [28s / 18s]:	1,8	Result Flagging Label:	RIN:10

Fragment table for sample 10 : K10

Name	Start Time [s]	End Time [s]	Area	% of total Area
18S	41,27	42,76	358,9	21,6
28S	47,14	50,36	652,4	39,3

 © Copyright 2003 - 2009 Agilent Technologies, Inc.

Assay Class:	Eukaryote Total RNA Nano	Created:	08.03.2017 10:01:40
Data Path:	E:\...Eukaryote Total RNA Nano_DE72901710_2017-03-08_10-01-40.xad	Modified:	08.03.2017 10:25:31

Electropherogram Summary Continued ...

K11

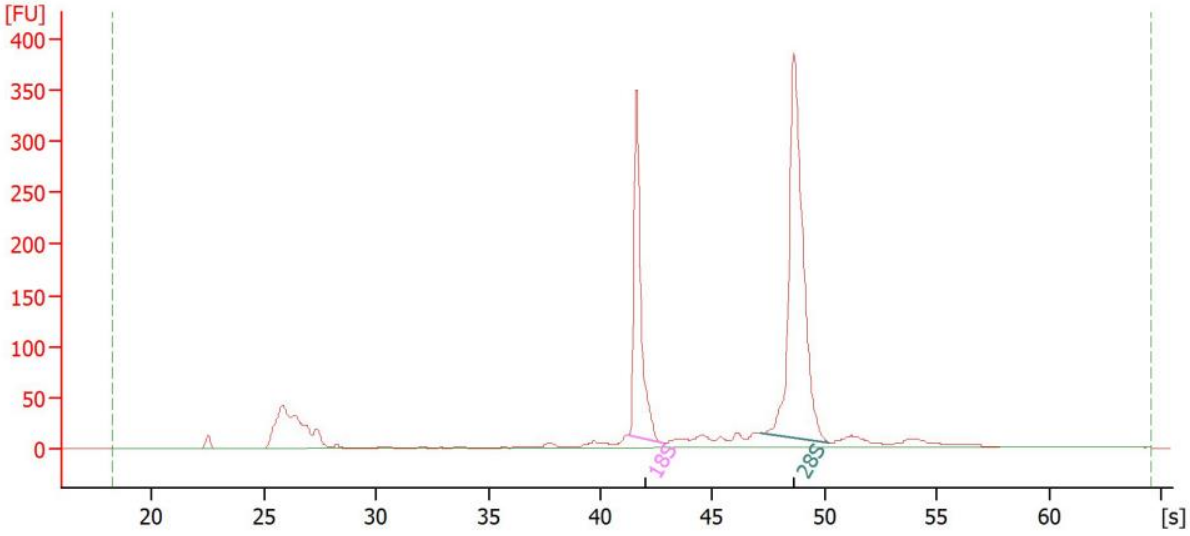

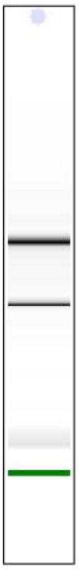

Overall Results for sample 11 : **K11**

RNA Area:	1.428,2	RNA Integrity Number (RIN):	9.9 (B.02.08)
RNA Concentration:	499 ng/µl	Result Flagging Color:	
rRNA Ratio [28s / 18s]:	1,8	Result Flagging Label:	RIN: 9.90

Fragment table for sample 11 : **K11**

Name	Start Time [s]	End Time [s]	Area	% of total Area
18S	41,26	42,75	306,4	21,5
28S	47,15	50,19	555,1	38,9

 © Copyright 2003 - 2009 Agilent Technologies, Inc.

Assay Class:	Eukaryote Total RNA Nano	Created:	08.03.2017 10:01:40
Data Path:	E:\...Eukaryote Total RNA Nano_DE72901710_2017-03-08_10-01-40.xad	Modified:	08.03.2017 10:25:31

Electropherogram Summary Continued ...

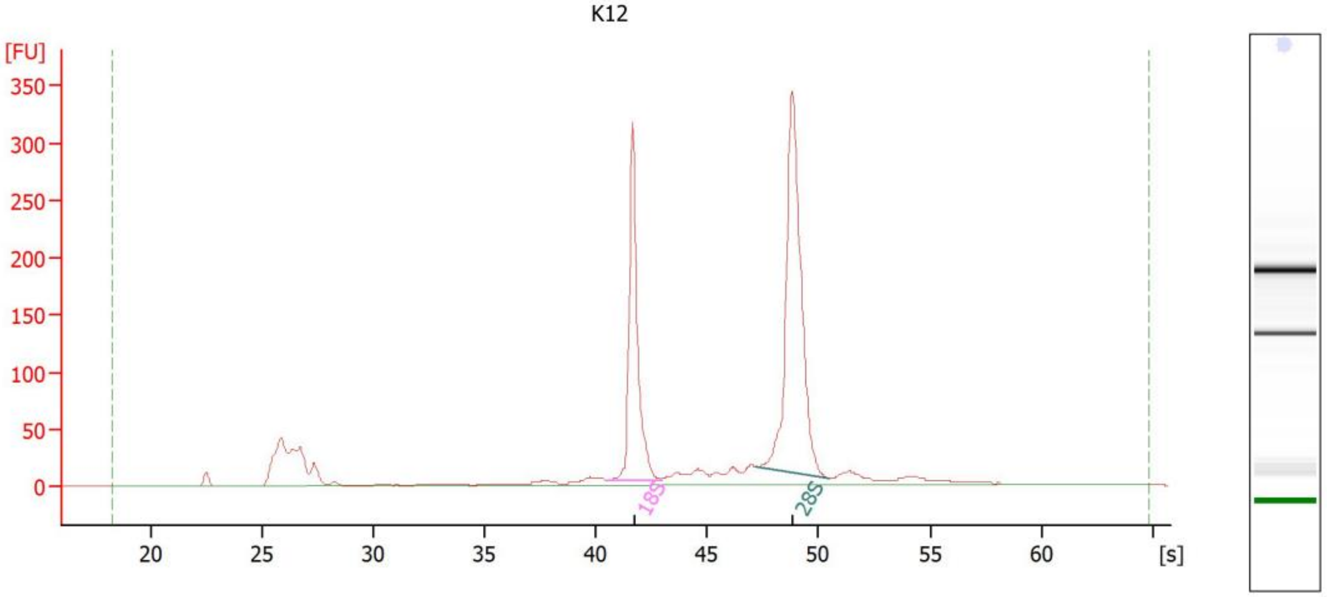

Overall Results for sample 12 : K12

RNA Area:	1.415,8	RNA Integrity Number (RIN):	9.9 (B.02.08)
RNA Concentration:	495 ng/µl	Result Flagging Color:	
rRNA Ratio [28s / 18s]:	1,7	Result Flagging Label:	RIN: 9.90

Fragment table for sample 12 : K12

Name	Start Time [s]	End Time [s]	Area	% of total Area
18S	40,54	42,93	318,9	22,5
28S	47,21	50,50	528,1	37,3

© Copyright 2003 - 2009 Agilent Technologies, Inc.

Assay Class: Eukaryote Total RNA Nano
Data Path: E:\...Eukaryote Total RNA Nano_DE72901710_2017-03-08_10-01-40.xad
Created: 08.03.2017 10:01:40
Modified: 08.03.2017 10:25:31

Gel Image

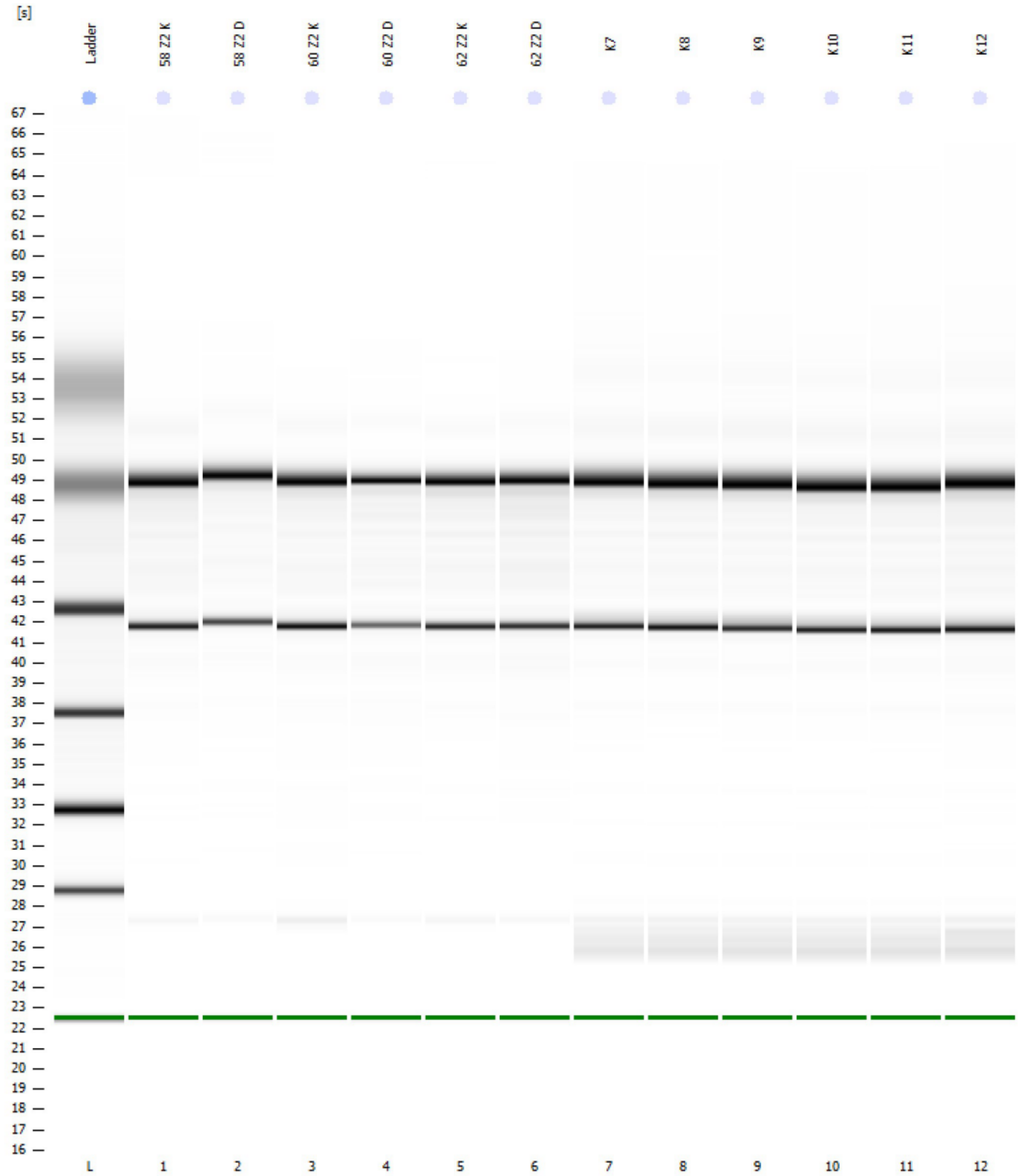

© Copyright 2003 - 2009 Agilent Technologies, Inc.

Assay Class: Eukaryote Total RNA Nano
Data Path: E:\...Eukaryote Total RNA Nano_DE72901710_2017-03-08_10-30-01.xad

Created: 08.03.2017 10:30:01
Modified: 08.03.2017 10:53:07

Electrophoresis File Run Summary

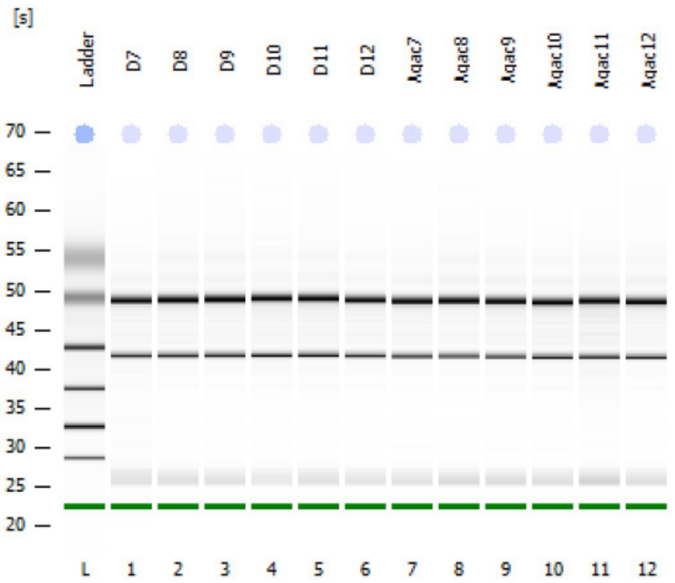

Instrument Information:

Instrument Name: DE72901710
Serial#: DE72901710
Firmware: C.01.069
Type: G2939A

Assay Information:

Assay Origin Path: C:\Programme\Agilent\2100 bioanalyzer\2100 expert\assays\RNA\Eukaryote Total RNA Nano Series II.xsy

Assay Class: Eukaryote Total RNA Nano

Version: 2.6

Assay Comments: Total RNA Analysis ng sensitivity (Eukaryote)

© Copyright 2003 - 2009 Agilent Technologies, Inc.

Chip Information:

Chip Lot #:

Reagent Kit Lot #:

Chip Comments:

D7
RIN: 9.50

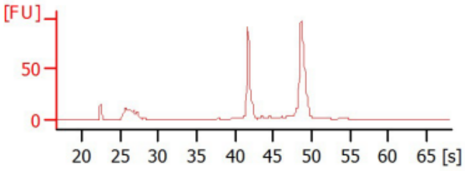

D8
RIN: 9.80

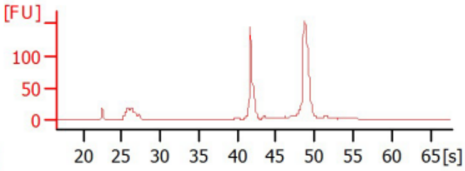

D9
RIN:10

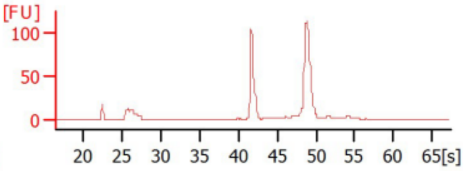

D10
RIN: 9.80

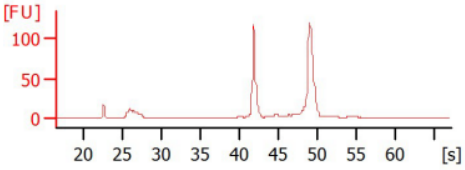

D11
RIN: 9.80

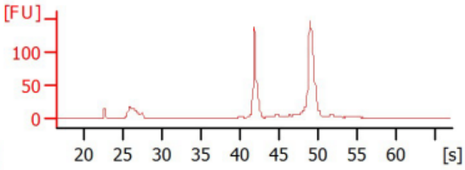

D12
RIN: 9.80

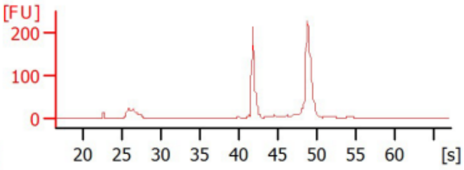

Agac7
RIN: 9.90

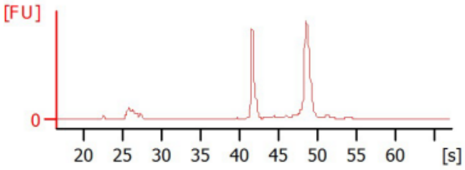

Agac8
RIN:10

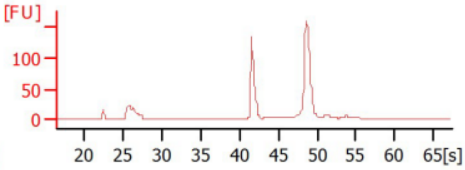

Agac9
RIN: 9.80

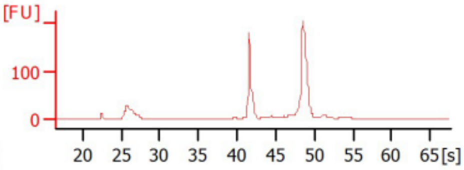

Agac10
RIN: 9.80

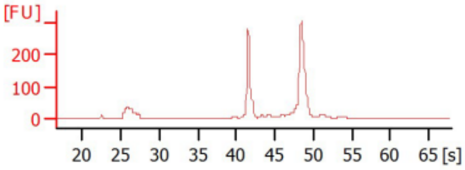

Agac11
RIN: 9.50

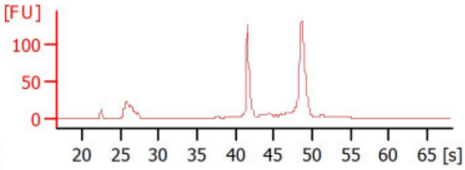

Agac12
RIN: 9.90

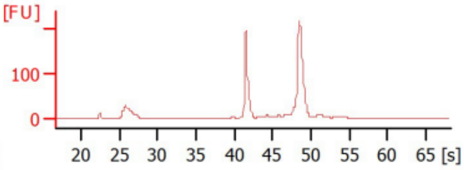

© Copyright 2003 - 2009 Agilent Technologies, Inc.

Assay Class: Eukaryote Total RNA Nano
Data Path: E:\...Eukaryote Total RNA Nano_DE72901710_2017-03-08_10-30-01.xad
Created: 08.03.2017 10:30:01
Modified: 08.03.2017 10:53:07

Electrophoresis File Run Summary (Chip Summary)

Sample Name	Sample Comment	Status	Result Label	Result Color
D7		✔	RIN: 9.50	
D8		✔	RIN: 9.80	
D9		✔	RIN:10	
D10		✔	RIN: 9.80	
D11		✔	RIN: 9.80	
D12		✔	RIN: 9.80	
Agac7		✔	RIN: 9.90	
Agac8		✔	RIN:10	
Agac9		✔	RIN: 9.80	
Agac10		✔	RIN: 9.80	
Agac11		✔	RIN: 9.50	
Agac12		✔	RIN: 9.90	
Ladder		✔	All Other Samples	

Chip Lot # **Reagent Kit Lot #**

Chip Comments :

 © Copyright 2003 - 2009 Agilent Technologies, Inc.

Assay Class: Eukaryote Total RNA Nano
Data Path: E:\...Eukaryote Total RNA Nano_DE72901710_2017-03-08_10-30-01.xad
Created: 08.03.2017 10:30:01
Modified: 08.03.2017 10:53:07

Electrophoresis Assay Details

General Analysis Settings

Number of Available Sample and Ladder Wells (Max.) : 13
Minimum Visible Range [s] : 17
Maximum Visible Range [s] : 70
Start Analysis Time Range [s] : 19
End Analysis Time Range [s] : 69
Ladder Concentration [ng/µl] : 150
Lower Marker Concentration [ng/µl] : 0
Upper Marker Concentration [ng/µl] : 0
Used Lower Marker for Quantitation
Standard Curve Fit is Logarithmic
Show Data Aligned to Lower Marker

Integrator Settings

Integration Start Time [s] : 19
Integration End Time [s] : 69
Slope Threshold : 0,6
Height Threshold [FU] : 0,5
Area Threshold : 0,2
Width Threshold [s] : 0,5
Baseline Plateau [s] : 6

Filter Settings

Filter Width [s] : 0,5
Polynomial Order : 4

Ladder

Ladder Peak	Size
1	25
2	200
3	500
4	1000
5	2000
6	4000

© Copyright 2003 - 2009 Agilent Technologies, Inc.

Assay Class:	Eukaryote Total RNA Nano	Created:	08.03.2017 10:30:01
Data Path:	E:\...Eukaryote Total RNA Nano_DE72901710_2017-03-08_10-30-01.xad	Modified:	08.03.2017 10:53:07

Electropherogram Summary

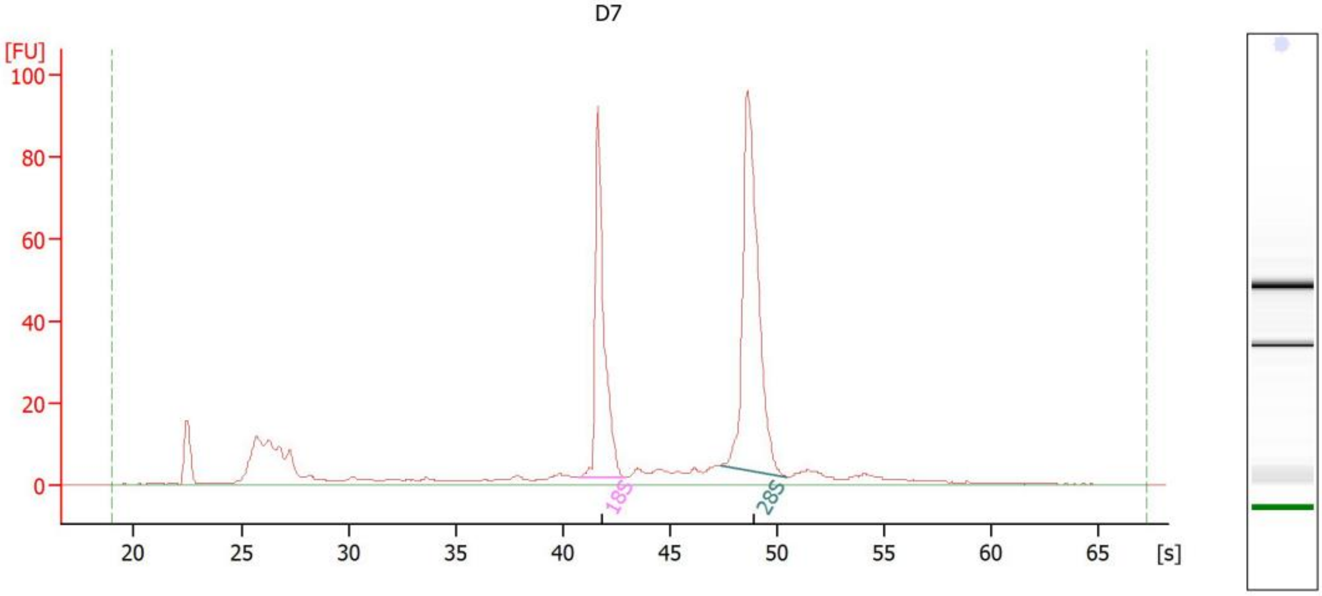

Overall Results for sample 1 : D7

RNA Area:	479,7	RNA Integrity Number (RIN):	9.5 (B.02.08)
RNA Concentration:	201 ng/µl	Result Flagging Color:	
rRNA Ratio [28s / 18s]:	1,6	Result Flagging Label:	RIN: 9.50

Fragment table for sample 1 : D7

Name	Start Time [s]	End Time [s]	Area	% of total Area
18S	40,71	42,91	97,1	20,2
28S	47,39	50,41	156,1	32,5

Assay Class:	Eukaryote Total RNA Nano	Created:	08.03.2017 10:30:01
Data Path:	E:\...Eukaryote Total RNA Nano_DE72901710_2017-03-08_10-30-01.xad	Modified:	08.03.2017 10:53:07

Electropherogram Summary Continued ...

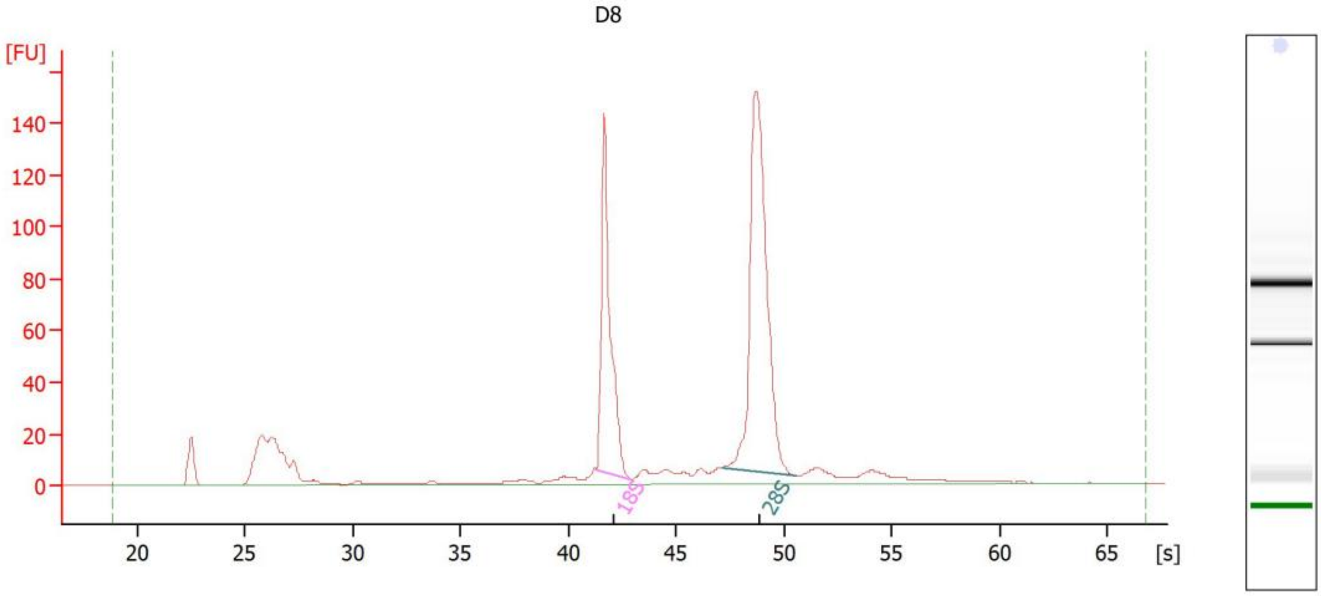

Overall Results for sample 2 : D8

RNA Area:	718,9	RNA Integrity Number (RIN):	9.8 (B.02.08)
RNA Concentration:	302 ng/µl	Result Flagging Color:	
rRNA Ratio [28s / 18s]:	1,7	Result Flagging Label:	RIN: 9.80

Fragment table for sample 2 : D8

Name	Start Time [s]	End Time [s]	Area	% of total Area
18S	41,32	42,92	153,6	21,4
28S	47,13	50,61	267,8	37,2

 © Copyright 2003 - 2009 Agilent Technologies, Inc.

Assay Class: Eukaryote Total RNA Nano
Data Path: E:\...Eukaryote Total RNA Nano_DE72901710_2017-03-08_10-30-01.xad

Created: 08.03.2017 10:30:01
Modified: 08.03.2017 10:53:07

Electropherogram Summary Continued ...

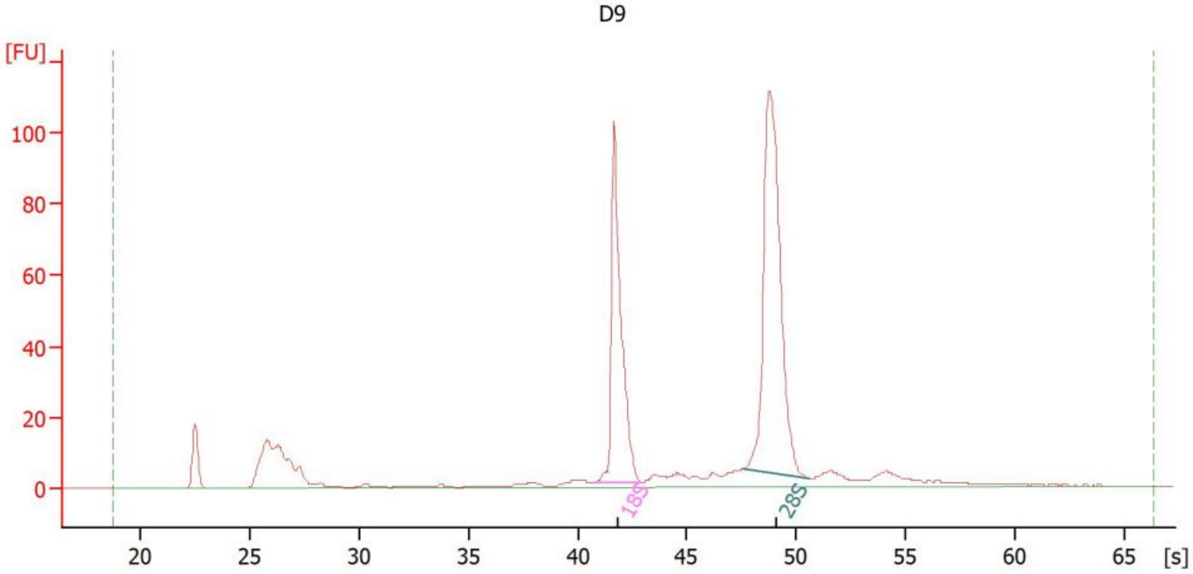

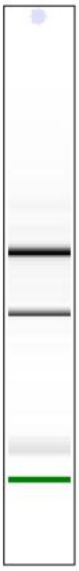

Overall Results for sample 3 : **D9**

RNA Area:	520,9	RNA Integrity Number (RIN):	10 (B.02.08)
RNA Concentration:	219 ng/µl	Result Flagging Color:	
rRNA Ratio [28s / 18s]:	1,6	Result Flagging Label:	RIN:10

Fragment table for sample 3 : **D9**

Name	Start Time [s]	End Time [s]	Area	% of total Area
18S	40,72	42,93	120,5	23,1
28S	47,60	50,58	196,5	37,7

 © Copyright 2003 - 2009 Agilent Technologies, Inc.

Assay Class:	Eukaryote Total RNA Nano	Created:	08.03.2017 10:30:01
Data Path:	E:\...Eukaryote Total RNA Nano_DE72901710_2017-03-08_10-30-01.xad	Modified:	08.03.2017 10:53:07

Electropherogram Summary Continued ...

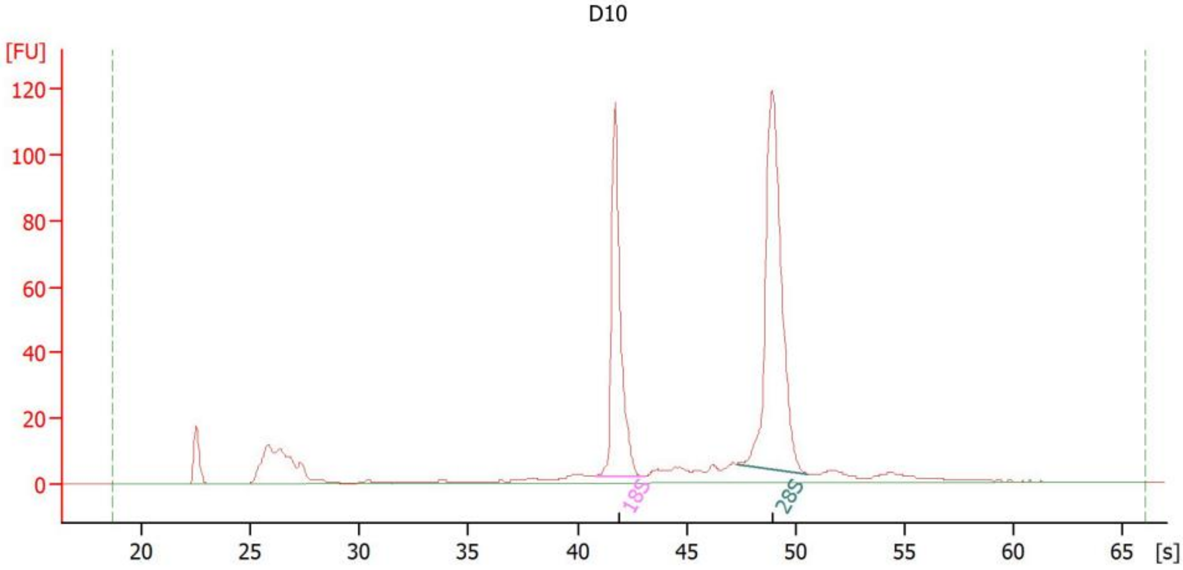

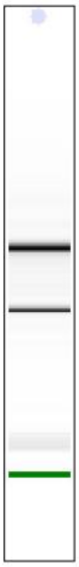

Overall Results for sample 4 : D10

RNA Area:	534,3	RNA Integrity Number (RIN):	9.8 (B.02.08)
RNA Concentration:	224 ng/µl	Result Flagging Color:	
rRNA Ratio [28s / 18s]:	1,6	Result Flagging Label:	RIN: 9.80

Fragment table for sample 4 : D10

Name	Start Time [s]	End Time [s]	Area	% of total Area
18S	40,84	43,04	122,9	23,0
28S	47,35	50,60	197,5	37,0

 © Copyright 2003 - 2009 Agilent Technologies, Inc.

Assay Class:	Eukaryote Total RNA Nano	Created:	08.03.2017 10:30:01
Data Path:	E:\...Eukaryote Total RNA Nano_DE72901710_2017-03-08_10-30-01.xad	Modified:	08.03.2017 10:53:07

Electropherogram Summary Continued ...

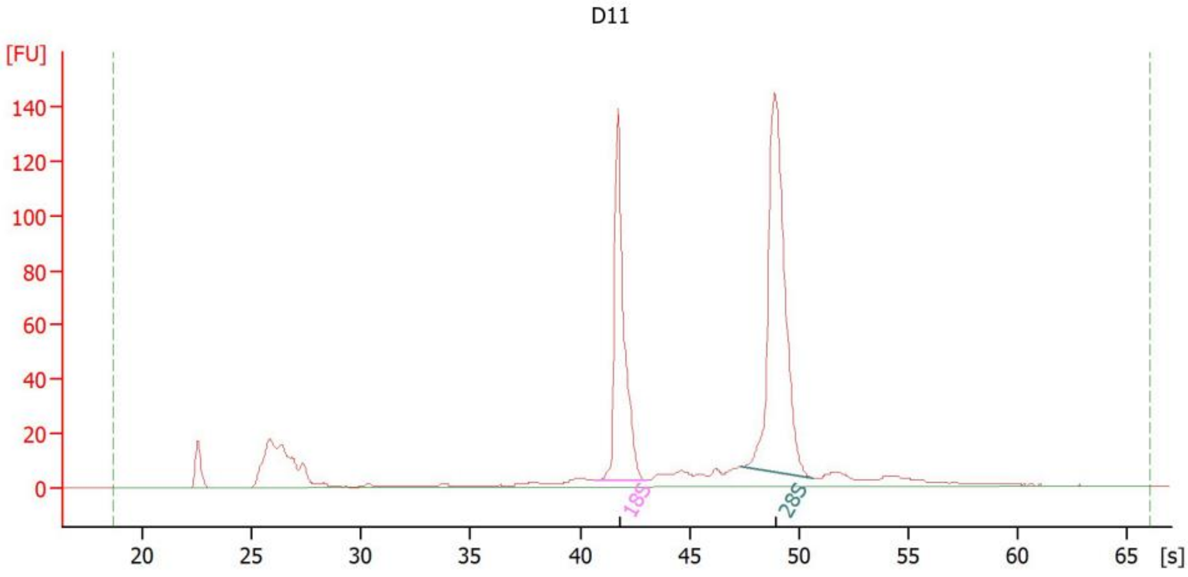

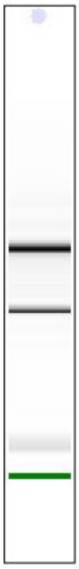

Overall Results for sample 5 : D11

RNA Area:	687,2	RNA Integrity Number (RIN):	9.8 (B.02.08)
RNA Concentration:	289 ng/µl	Result Flagging Color:	
rRNA Ratio [28s / 18s]:	1,6	Result Flagging Label:	RIN: 9.80

Fragment table for sample 5 : D11

Name	Start Time [s]	End Time [s]	Area	% of total Area
18S	40,69	43,09	156,2	22,7
28S	47,39	50,65	247,1	36,0

© Copyright 2003 - 2009 Agilent Technologies, Inc.

Assay Class:	Eukaryote Total RNA Nano	Created:	08.03.2017 10:30:01
Data Path:	E:\...Eukaryote Total RNA Nano_DE72901710_2017-03-08_10-30-01.xad	Modified:	08.03.2017 10:53:07

Electropherogram Summary Continued ...

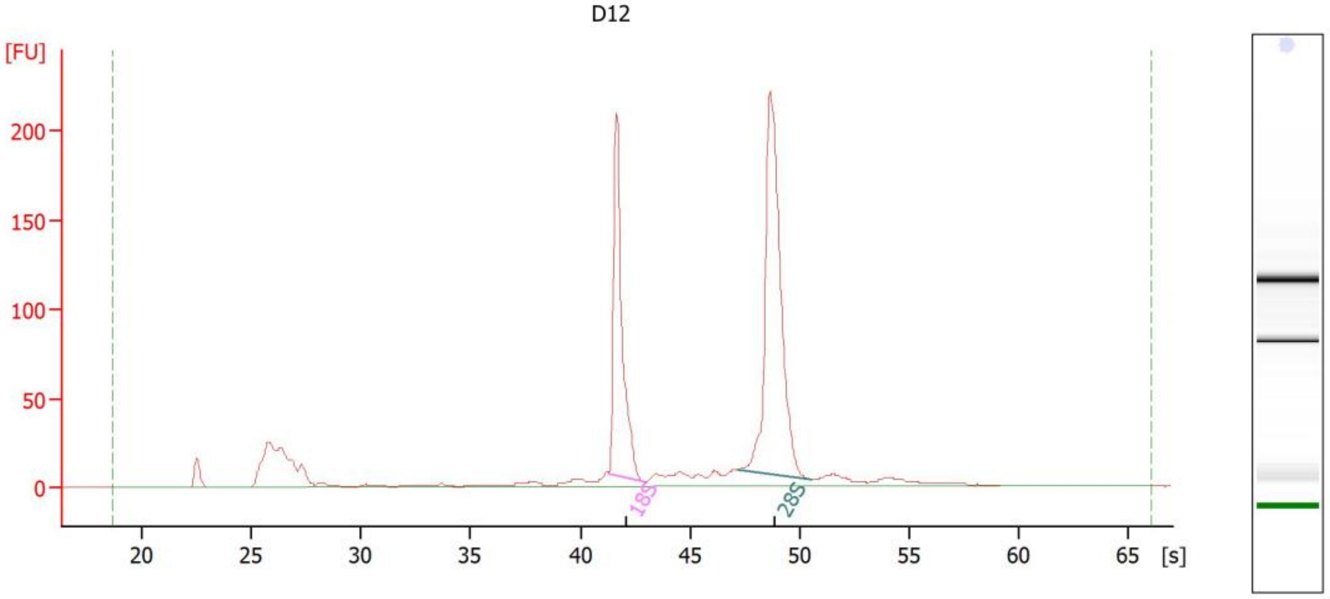

Overall Results for sample 6 : D12

RNA Area:	942,6	RNA Integrity Number (RIN):	9.8 (B.02.08)
RNA Concentration:	396 ng/µl	Result Flagging Color:	
rRNA Ratio [28s / 18s]:	1,7	Result Flagging Label:	RIN: 9.80

Fragment table for sample 6 : D12

Name	Start Time [s]	End Time [s]	Area	% of total Area
18S	41,31	42,89	207,1	22,0
28S	47,15	50,55	357,4	37,9

© Copyright 2003 - 2009 Agilent Technologies, Inc.

Assay Class: Eukaryote Total RNA Nano
Data Path: E:\...Eukaryote Total RNA Nano_DE72901710_2017-03-08_10-30-01.xad
Created: 08.03.2017 10:30:01
Modified: 08.03.2017 10:53:07

Electropherogram Summary Continued ...

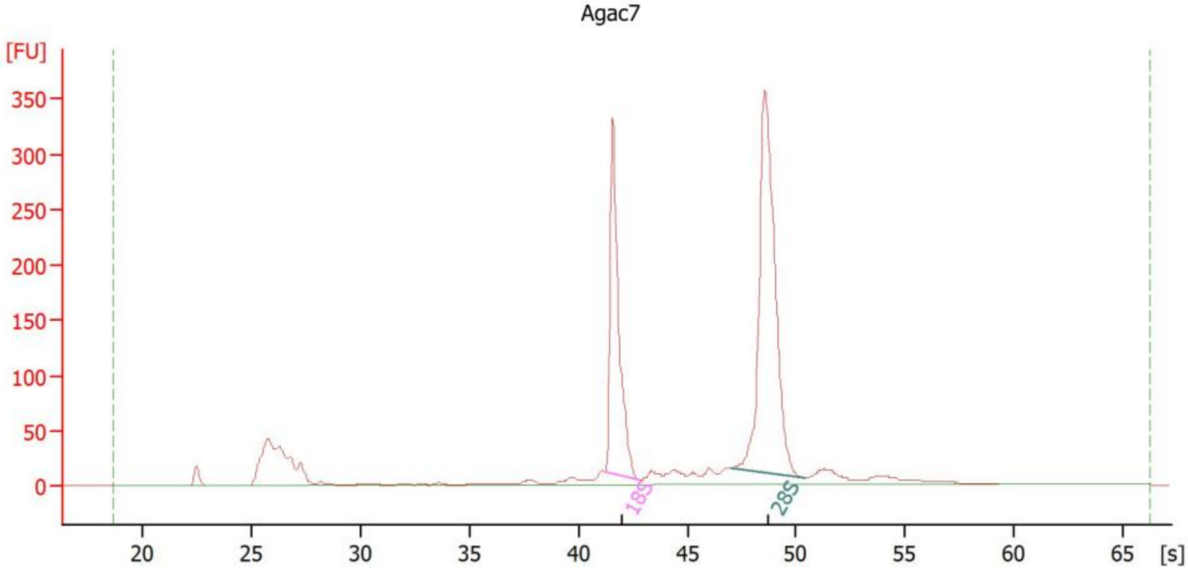

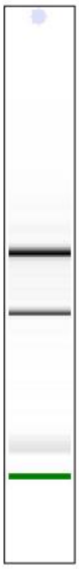

Overall Results for sample 7 : Agac7

RNA Area:	1.565,2	RNA Integrity Number (RIN):	9.9 (B.02.08)
RNA Concentration:	657 ng/µl	Result Flagging Color:	
rRNA Ratio [28s / 18s]:	1,7	Result Flagging Label:	RIN: 9.90

Fragment table for sample 7 : Agac7

Name	Start Time [s]	End Time [s]	Area	% of total Area
18S	41,26	42,79	341,8	21,8
28S	47,01	50,37	595,8	38,1

 © Copyright 2003 - 2009 Agilent Technologies, Inc.

Assay Class: Eukaryote Total RNA Nano
Data Path: E:\...Eukaryote Total RNA Nano_DE72901710_2017-03-08_10-30-01.xad
Created: 08.03.2017 10:30:01
Modified: 08.03.2017 10:53:07

Electropherogram Summary Continued ...

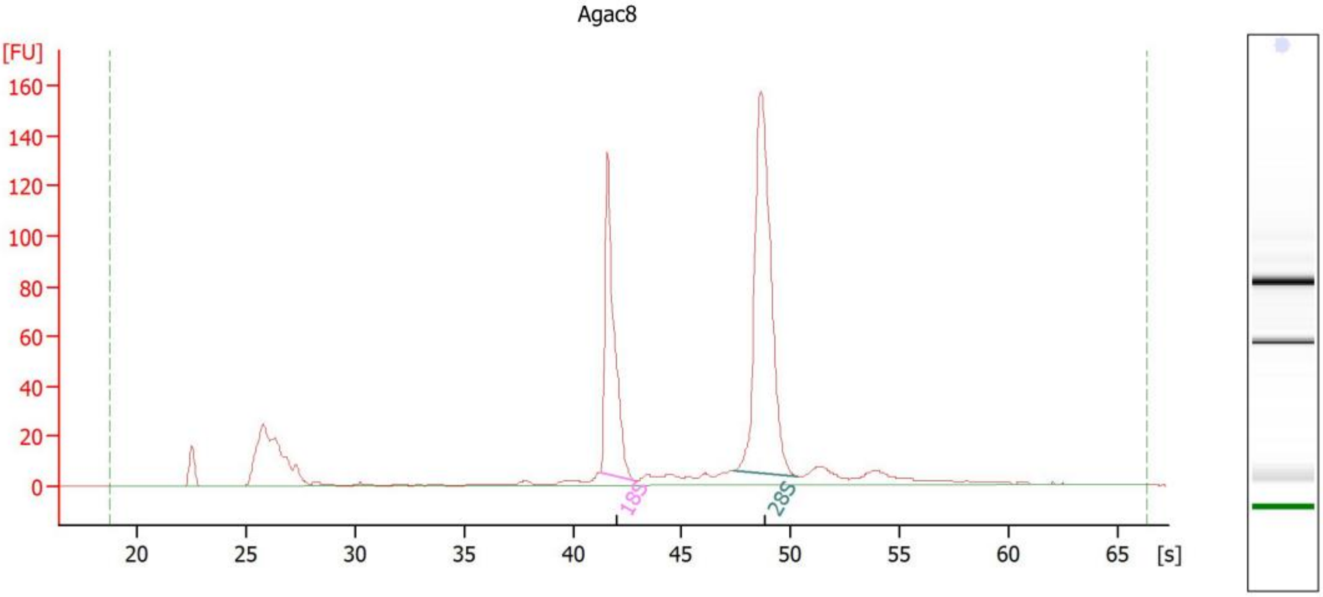

Overall Results for sample 8 : **Agac8**

RNA Area:	713,4	RNA Integrity Number (RIN):	10 (B.02.08)
RNA Concentration:	300 ng/µl	Result Flagging Color:	
rRNA Ratio [28s / 18s]:	1,8	Result Flagging Label:	RIN:10

Fragment table for sample 8 : **Agac8**

Name	Start Time [s]	End Time [s]	Area	% of total Area
18S	41,25	42,84	147,8	20,7
28S	47,36	50,34	267,2	37,5

 © Copyright 2003 - 2009 Agilent Technologies, Inc.

Assay Class: Eukaryote Total RNA Nano Created: 08.03.2017 10:30:01
Data Path: E:\...Eukaryote Total RNA Nano_DE72901710_2017-03-08_10-30-01.xad Modified: 08.03.2017 10:53:07

Electropherogram Summary Continued ...

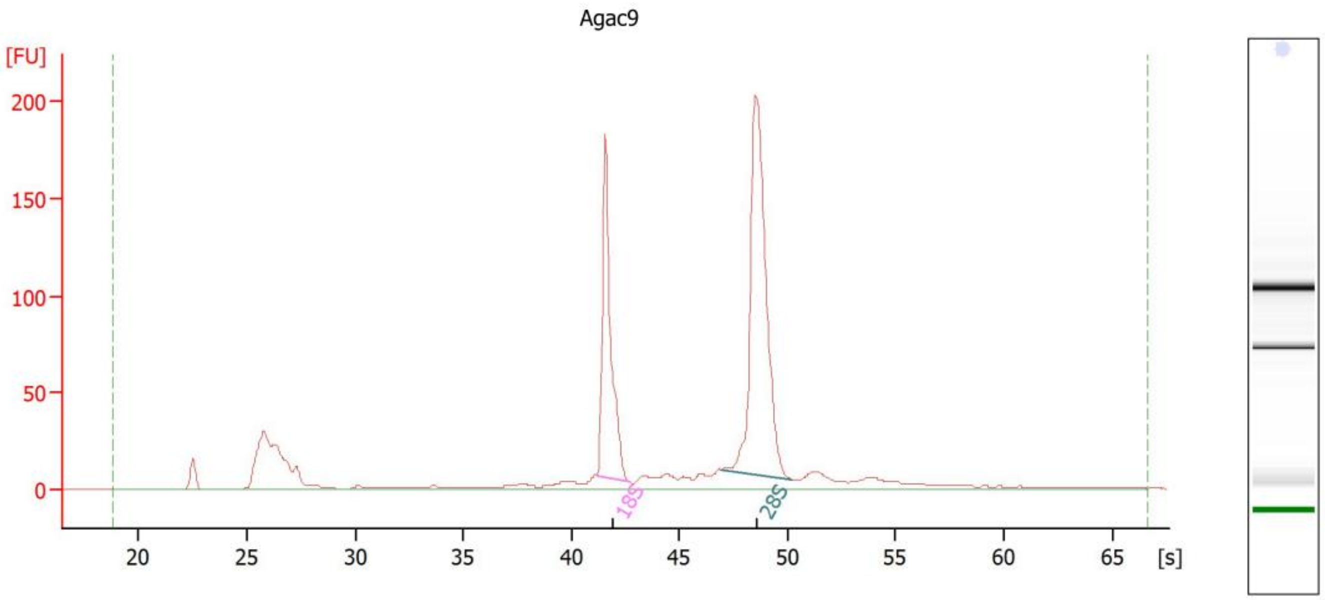

Overall Results for sample 9 : Agac9

RNA Area:	944,4	RNA Integrity Number (RIN):	9.8 (B.02.08)
RNA Concentration:	397 ng/µl	Result Flagging Color:	
rRNA Ratio [28s / 18s]:	1,8	Result Flagging Label:	RIN: 9.80

Fragment table for sample 9 : Agac9

Name	Start Time [s]	End Time [s]	Area	% of total Area
18S	41,23	42,73	186,9	19,8
28S	46,98	50,21	333,0	35,3

Assay Class:	Eukaryote Total RNA Nano	Created:	08.03.2017 10:30:01
Data Path:	E:\...Eukaryote Total RNA Nano_DE72901710_2017-03-08_10-30-01.xad	Modified:	08.03.2017 10:53:07

Electropherogram Summary Continued ...

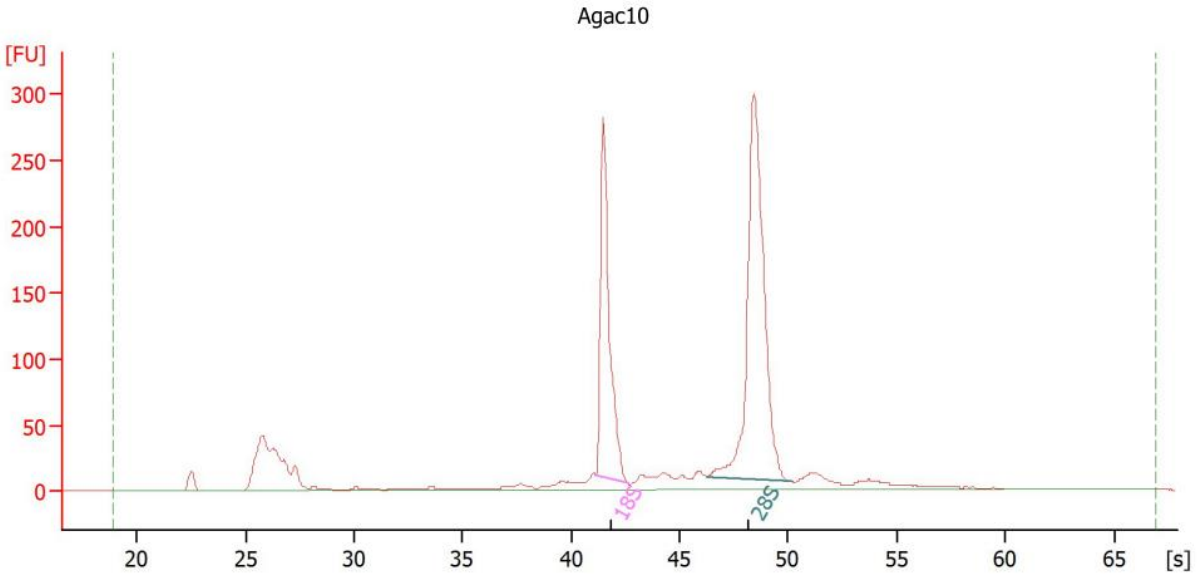

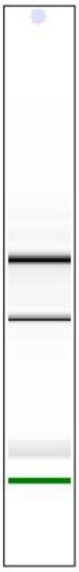

Overall Results for sample 10 : Agac10

RNA Area:	1.401,0	RNA Integrity Number (RIN):	9.8 (B.02.08)
RNA Concentration:	588 ng/µl	Result Flagging Color:	
rRNA Ratio [28s / 18s]:	1,8	Result Flagging Label:	RIN: 9.80

Fragment table for sample 10 : Agac10

Name	Start Time [s]	End Time [s]	Area	% of total Area
18S	41,12	42,62	294,2	21,0
28S	46,21	50,14	527,0	37,6

 © Copyright 2003 - 2009 Agilent Technologies, Inc.

Assay Class: Eukaryote Total RNA Nano
Data Path: E:\...Eukaryote Total RNA Nano_DE72901710_2017-03-08_10-30-01.xad

Created: 08.03.2017 10:30:01
Modified: 08.03.2017 10:53:07

Electropherogram Summary Continued ...

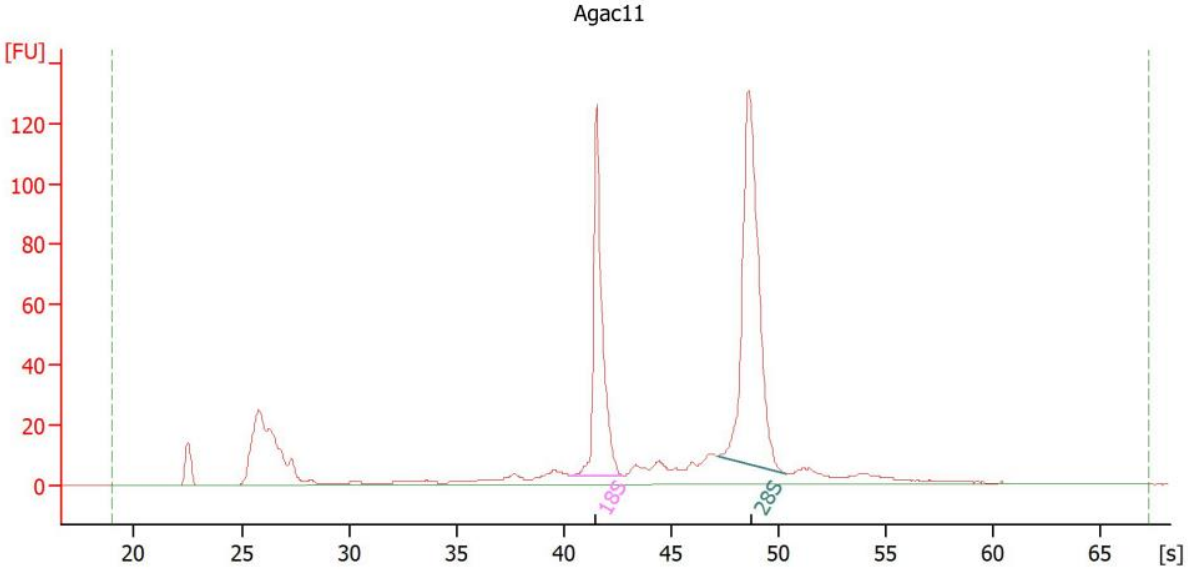

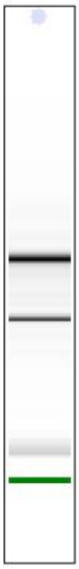

Overall Results for sample 11 : Agac11

RNA Area:	705,5	RNA Integrity Number (RIN):	9.5 (B.02.08)
RNA Concentration:	296 ng/µl	Result Flagging Color:	
rRNA Ratio [28s / 18s]:	1,6	Result Flagging Label:	RIN: 9.50

Fragment table for sample 11 : Agac11

Name	Start Time [s]	End Time [s]	Area	% of total Area
18S	40,32	42,76	133,9	19,0
28S	47,14	50,36	218,9	31,0

 © Copyright 2003 - 2009 Agilent Technologies, Inc.

Assay Class: Eukaryote Total RNA Nano
Data Path: E:\...Eukaryote Total RNA Nano_DE72901710_2017-03-08_10-30-01.xad

Created: 08.03.2017 10:30:01
Modified: 08.03.2017 10:53:07

Electropherogram Summary Continued ...

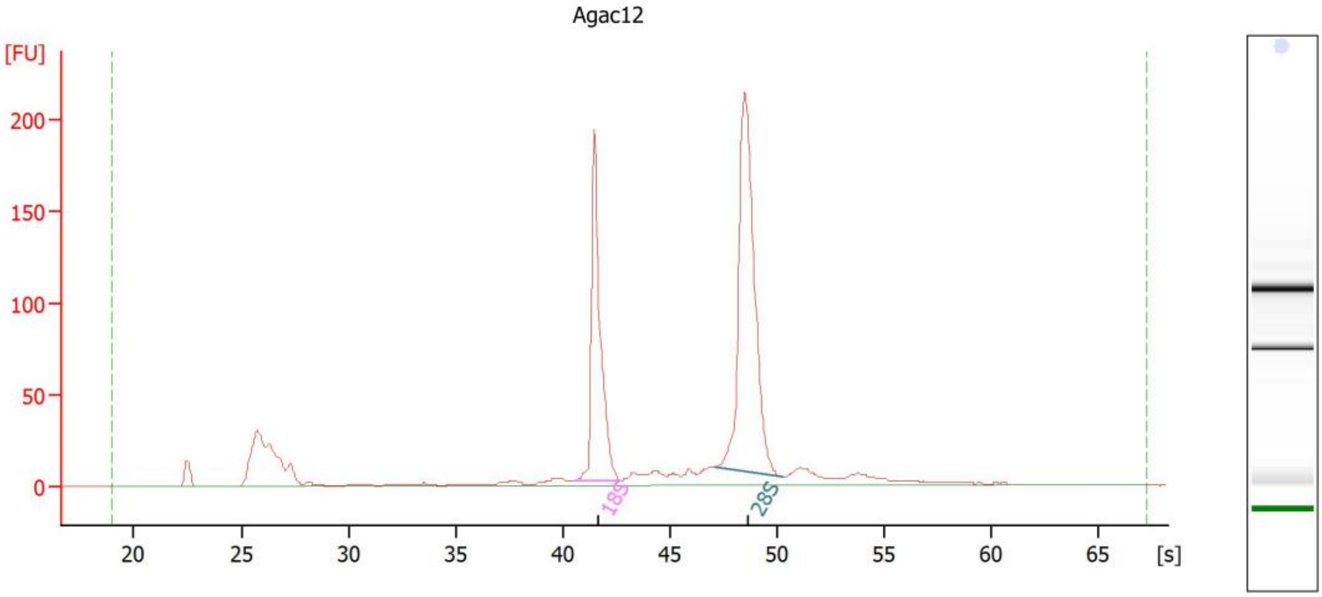

Overall Results for sample 12 : Agac12

RNA Area:	985,1	RNA Integrity Number (RIN):	9.9 (B.02.08)
RNA Concentration:	414 ng/µl	Result Flagging Color:	
rRNA Ratio [28s / 18s]:	1,6	Result Flagging Label:	RIN: 9.90

Fragment table for sample 12 : Agac12

Name	Start Time [s]	End Time [s]	Area	% of total Area
18S	40,52	42,71	216,0	21,9
28S	47,09	50,26	354,0	35,9

© Copyright 2003 - 2009 Agilent Technologies, Inc.

Assay Class: Eukaryote Total RNA Nano
Data Path: E:\...Eukaryote Total RNA Nano_DE72901710_2017-03-08_10-30-01.xad
Created: 08.03.2017 10:30:01
Modified: 08.03.2017 10:53:07

Gel Image

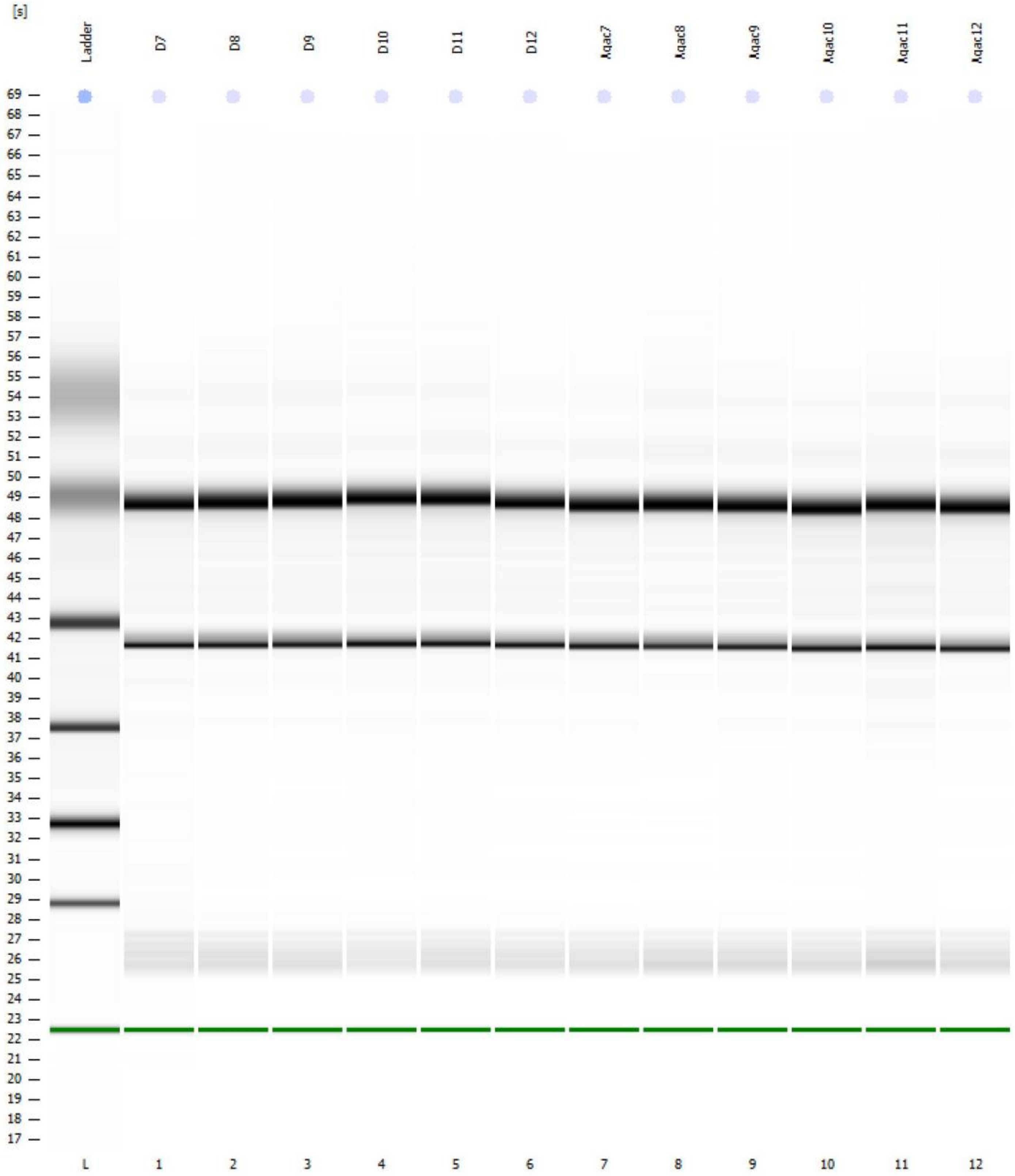

 © Copyright 2003 - 2009 Agilent Technologies, Inc. Printed: 08.03.2017 10:39:38

Supplementary Data 3. Amplification plot and Melting curve analysis (RT-qPCR).

qPCR program (used in all qPCR runs)

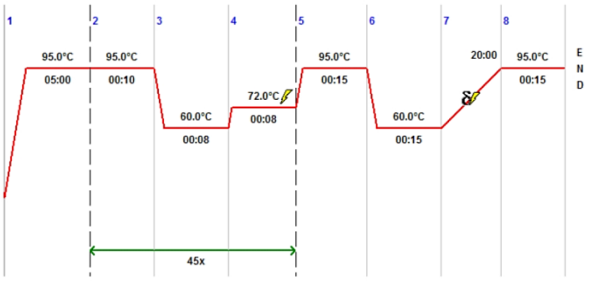

GAPDH (main qPCR)

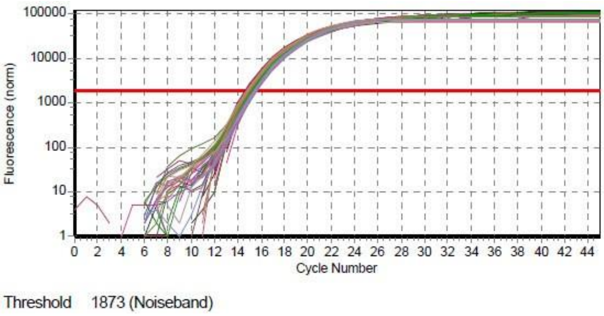

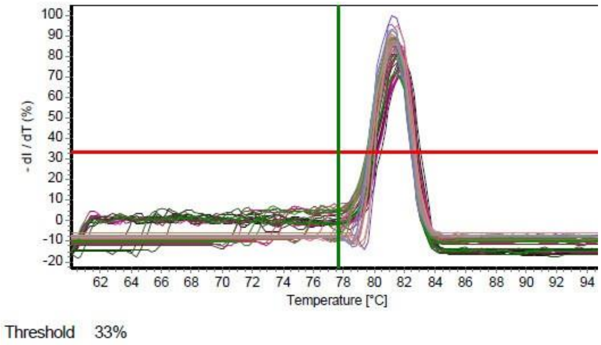

Amplification plot

Melting curve

PPIB (main qPCR)

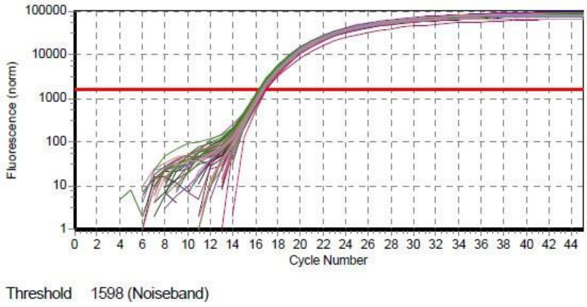

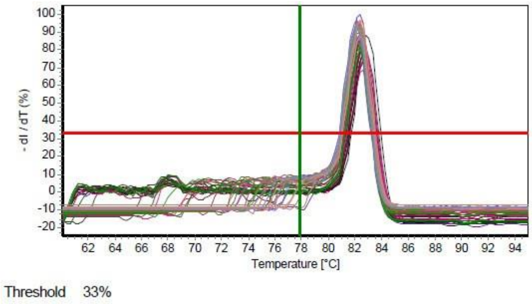

Amplification plot

Melting curve

YWHAZ (main qPCR)

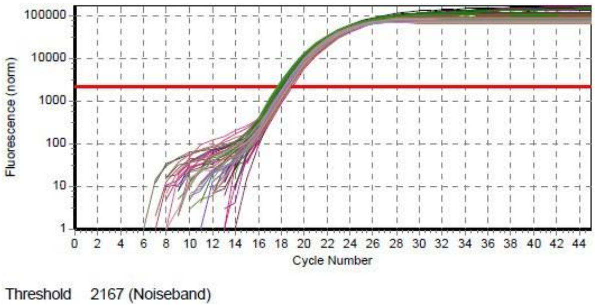

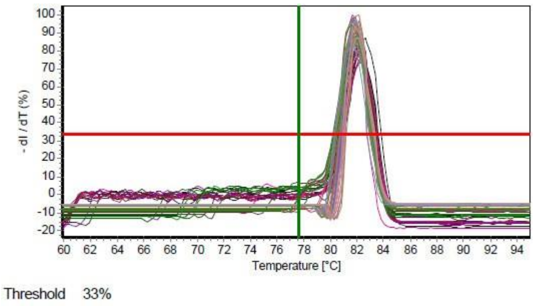

Amplification plot

Melting curve

POLR2A (main qPCR)

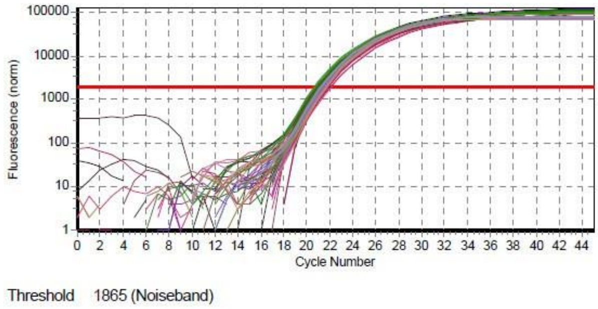

Amplification plot

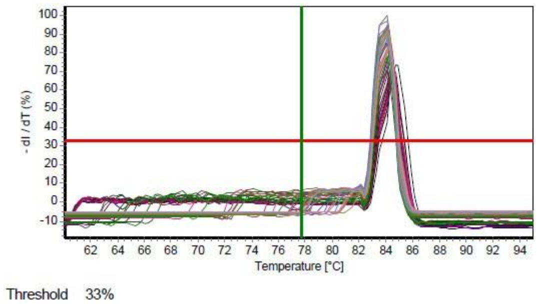

Melting curve

TBP (main qPCR)

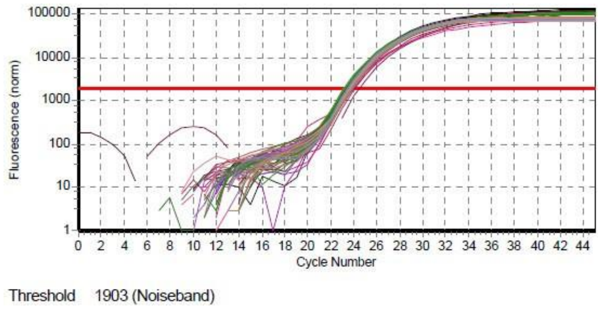

Amplification plot

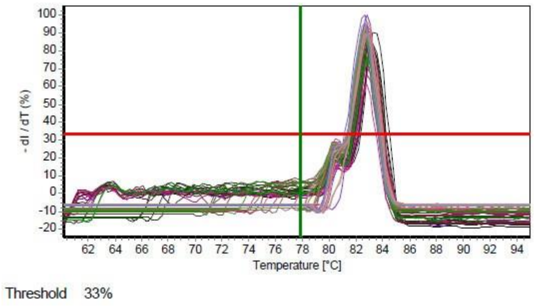

Melting curve

RPL22 (main qPCR)

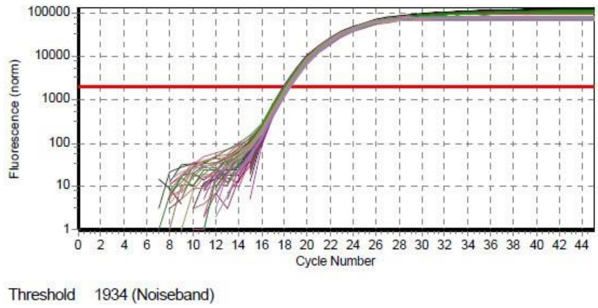

Amplification plot

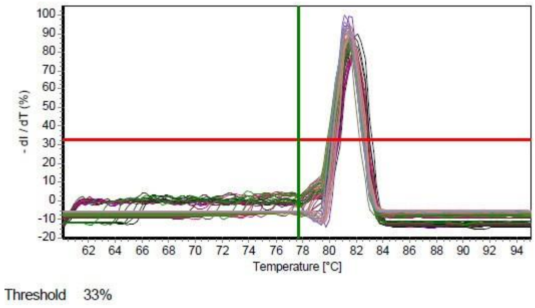

Melting curve

EEF1A1 (main qPCR)

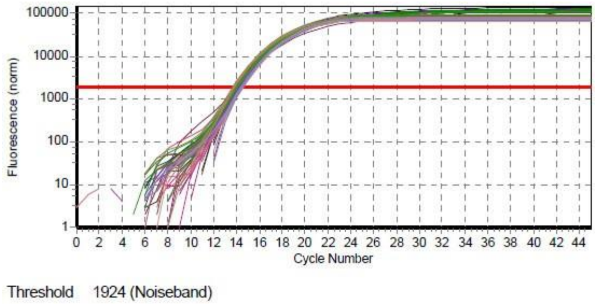

Amplification plot

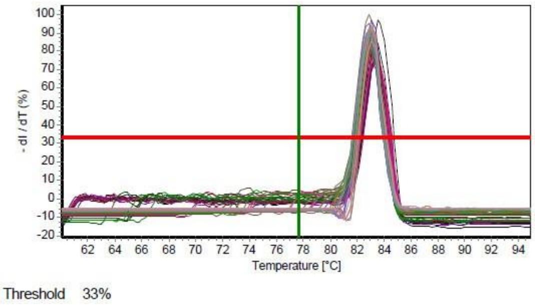

Melting curve

RPLP0 (main qPCR)

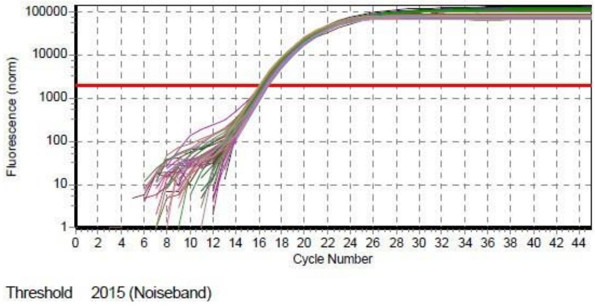

Amplification plot

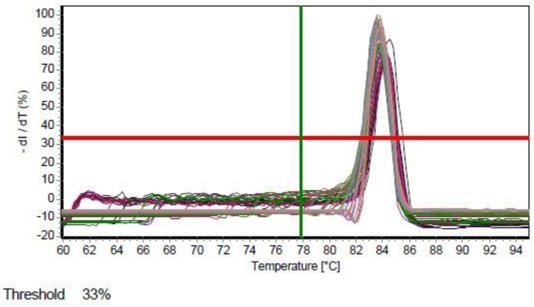

Melting curve

RNA18S5 (main qPCR)

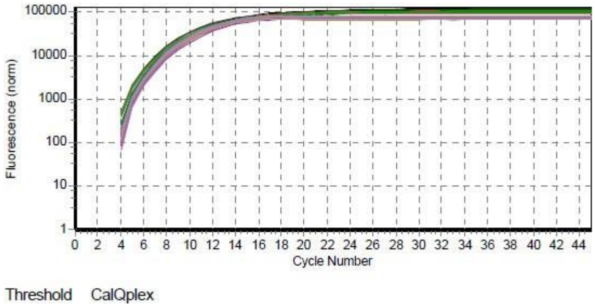

Amplification plot

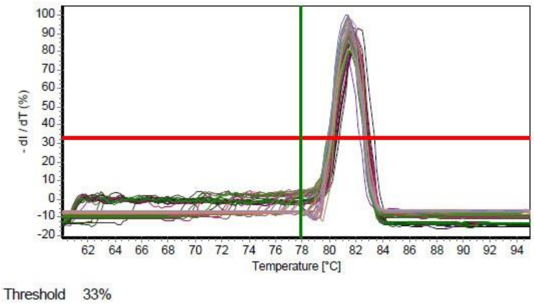

Melting curve

GAPDH (efficiency qPCR – standard curve)

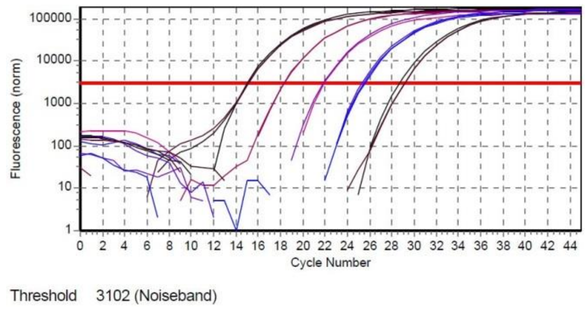

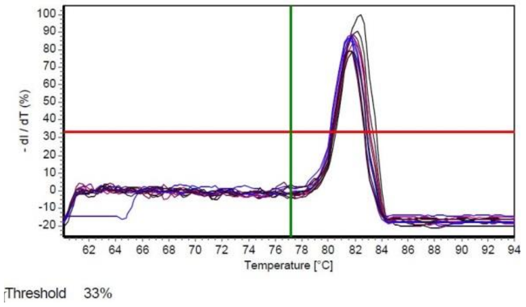

Amplification plot

Melting curve

PPIB (efficiency qPCR – standard curve)

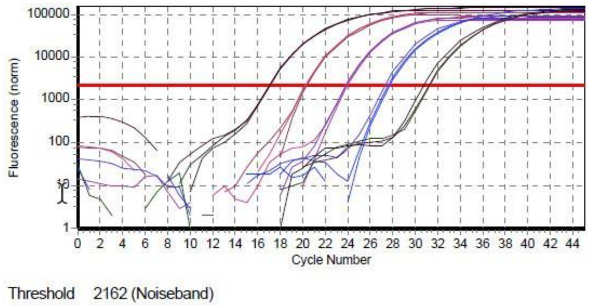

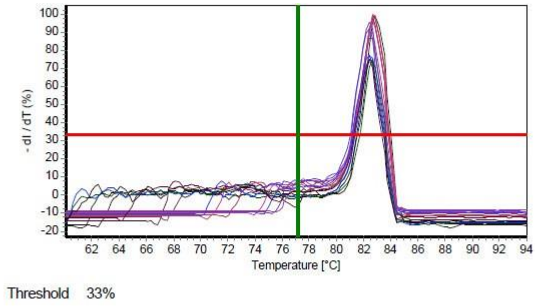

Amplification plot

Melting curve

YWHAZ (efficiency qPCR – standard curve)

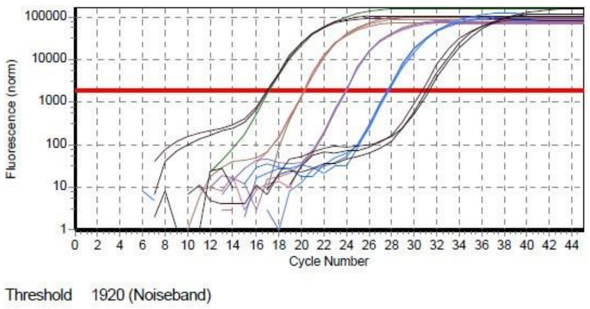

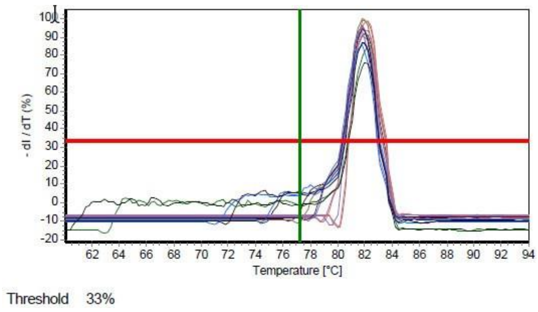

Amplification plot

Melting curve

POLR2A (efficiency qPCR – standard curve)

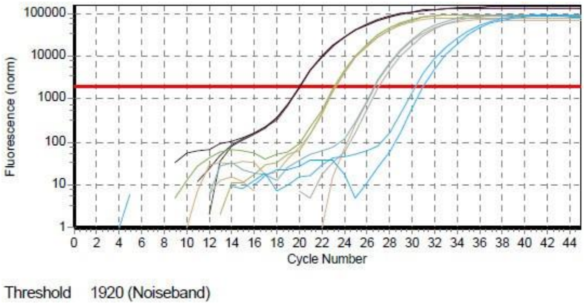

Amplification plot

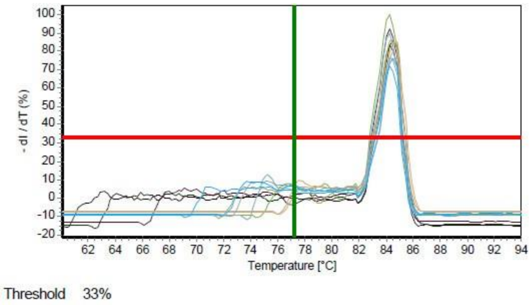

Melting curve

TBP (efficiency qPCR – standard curve)

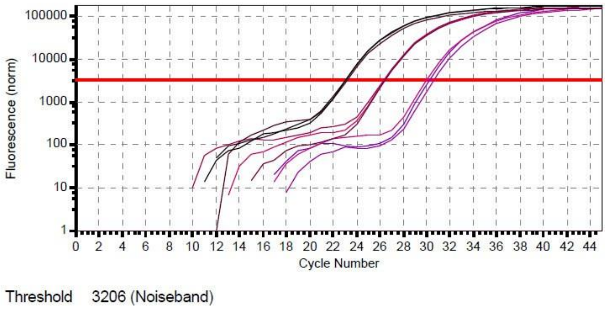

Amplification plot

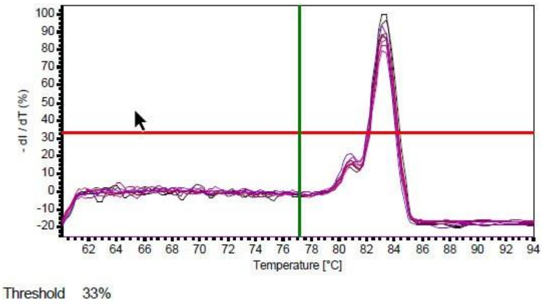

Melting curve

RPL22 (efficiency qPCR – standard curve)

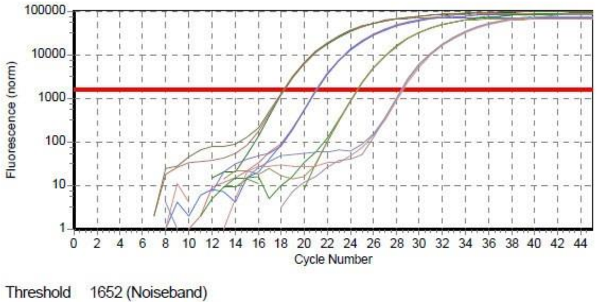

Amplification plot

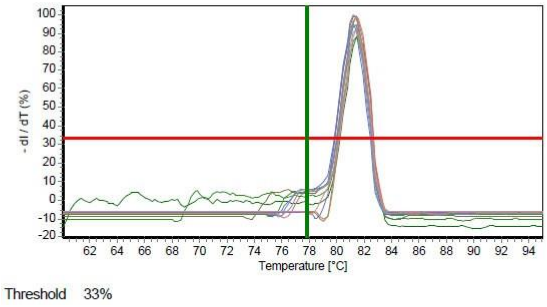

Melting curve

EEF1A1 (efficiency qPCR – standard curve)

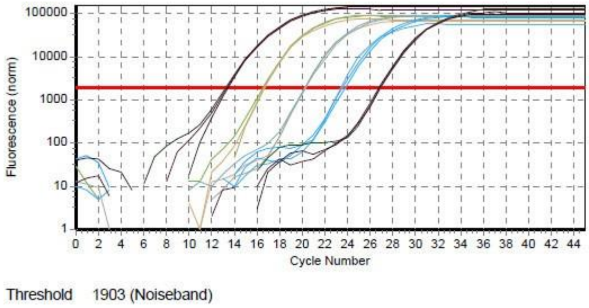

Amplification plot

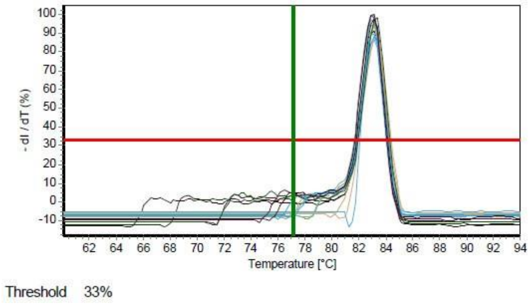

Melting curve

RPLP0 (efficiency qPCR – standard curve)

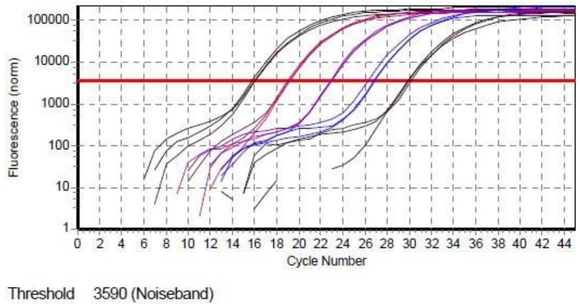

Amplification plot

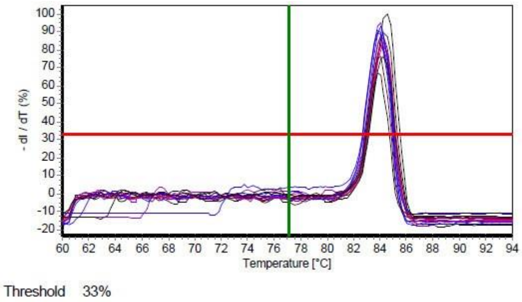

Melting curve

RNA18S5 (efficiency qPCR – standard curve)

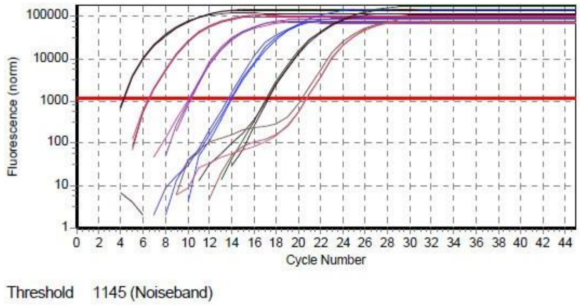

Amplification plot

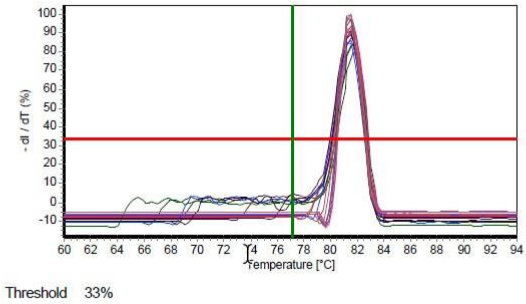

Melting curve

Supplementary Data 4. Evaluation of qPCR primer efficiency (factor-specific). Log_{10} serial dilution of cDNA stock solution (1,000,000 pg RNA equivalent) was performed in triplets. From the resulting C_q values a standard curve was created by linear regression.

<u>GAPDH</u> (factor-specific primer efficiency)

Gene	RNA equivalent [pg]	C_q Triplet	C_q 95%CI	C_q Mean	C_q SD	cDNA dilution
GAPDH	100000	14.60	14.56 /14.69	14.627	0.025	1:10
GAPDH	100000	14.65				
GAPDH	100000	14.63				
GAPDH	10000	17.91	17.74 /18.15	17.947	0.081	$1:10^2$
GAPDH	10000	17.89				
GAPDH	10000	18.04				
GAPDH	1000	21.56	21.11 /21.76	21.433	0.130	$1:10^3$
GAPDH	1000	21.30				
GAPDH	1000	21.44				
GAPDH	100	24.90	24.74 /25.29	25.017	0.111	$1:10^4$
GAPDH	100	25.03				
GAPDH	100	25.12				
GAPDH	10	28.27	27.95 /29.14	28.547	0.240	$1:10^5$
GAPDH	10	28.69				
GAPDH	10	28.68				
GAPDH	1	33.26	29.80 /34.53	32.163	0.952	$1:10^6$
GAPDH	1	31.55				
GAPDH	1	31.68				
GAPDH	NTC	39.62				

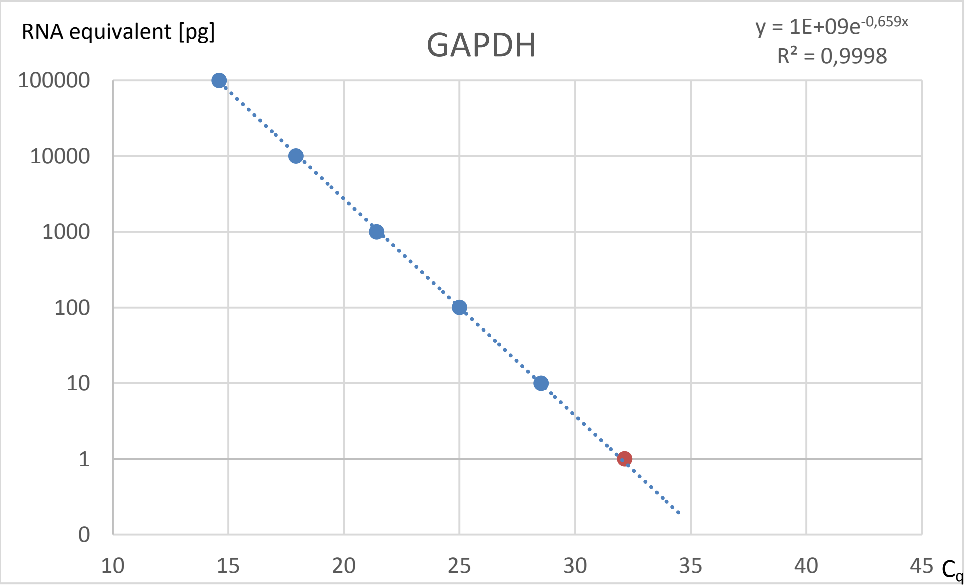

Slope [95% CI]	% Efficiency [95% CI]	LDR (dilution range)	LOD (dilution)
-3.480 [-3.661/-3.299]	93.8 [87.6/101.0]	$1:10 – 1:10^5$	$\leq 1:10^6$

SD = standard deviation; NTC = no-template control; LDR = linear dynamic range; LOD = limit of detection; R^2 = coefficient of determination; CI = confidence interval

<u>PPIB</u> (factor-specific primer efficiency)

Gene	RNA equivalent [pg]	C_q Triplet	C_q 95%CI	C_q Mean	C_q SD	cDNA dilution
PPIB	100000	16.91	16.88 /16.92	16.900	0.010	1:10
PPIB	100000	16.90				
PPIB	100000	16.89				
PPIB	10000	20.21	20.00 /20.29	20.143	0.058	$1:10^2$
PPIB	10000	20.11				
PPIB	10000	20.11				
PPIB	1000	23.72	23.60 /23.90	23.750	0.061	$1:10^3$
PPIB	1000	23.82				
PPIB	1000	23.71				
PPIB	100	27.19	26.90 /27.94	27.420	0.210	$1:10^4$
PPIB	100	27.47				
PPIB	100	27.60				
PPIB	10	31.14	30.21 /31.66	30.933	0.291	$1:10^5$
PPIB	10	30.60				
PPIB	10	31.06				
PPIB	1	33.62	31.18 /39.97	35.573	1.770	$1:10^6$
PPIB	1	36.03				
PPIB	1	37.07				
PPIB	NTC	35.92				

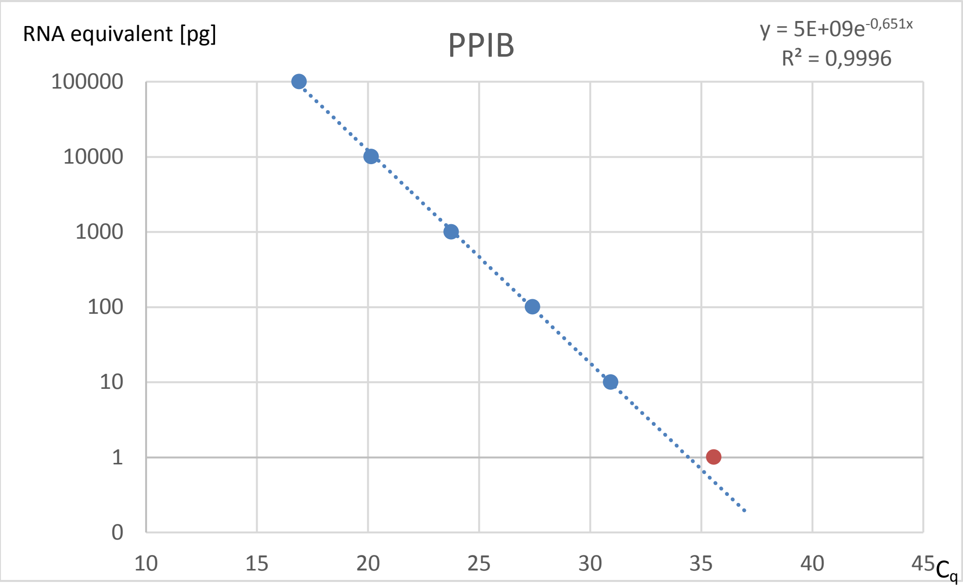

Slope [95% CI]	% Efficiency [95% CI]	LDR (dilution range)	LOD (dilution)
-3.509 [-3.808/-3.209]	92.7 [83.1/104.9]	$1:10 – 1:10^5$	$\leq 1:10^6$

SD = standard deviation; NTC = no-template control; LDR = linear dynamic range; LOD = limit of detection; R^2 = coefficient of determination; CI = confidence interval

YWHAZ (factor-specific primer efficiency)

Gene	RNA equivalent [pg]	C_q Triplet	C_q 95%CI	C_q Mean	C_q SD	cDNA dilution
YWHAZ	100000	17.20	16.85 /17.35	17.100	0.100	1:10
YWHAZ	100000	17.10				
YWHAZ	100000	17.00				
YWHAZ	10000	20.33	20.21 /20.39	20.300	0.036	$1:10^2$
YWHAZ	10000	20.26				
YWHAZ	10000	20.31				
YWHAZ	1000	24.04	23.94 /24.08	24.010	0.030	$1:10^3$
YWHAZ	1000	23.98				
YWHAZ	1000	24.01				
YWHAZ	100	27.72	27.53 /27.93	27.733	0.081	$1:10^4$
YWHAZ	100	27.66				
YWHAZ	100	27.82				
YWHAZ	10	31.34	30.26 /31.85	31.053	0.319	$1:10^5$
YWHAZ	10	31.11				
YWHAZ	10	30.71				
YWHAZ	1	36.90	30.73 /39.16	34.943	1.696	$1:10^6$
YWHAZ	1	34.03				
YWHAZ	1	33.90				
YWHAZ	NTC	-				

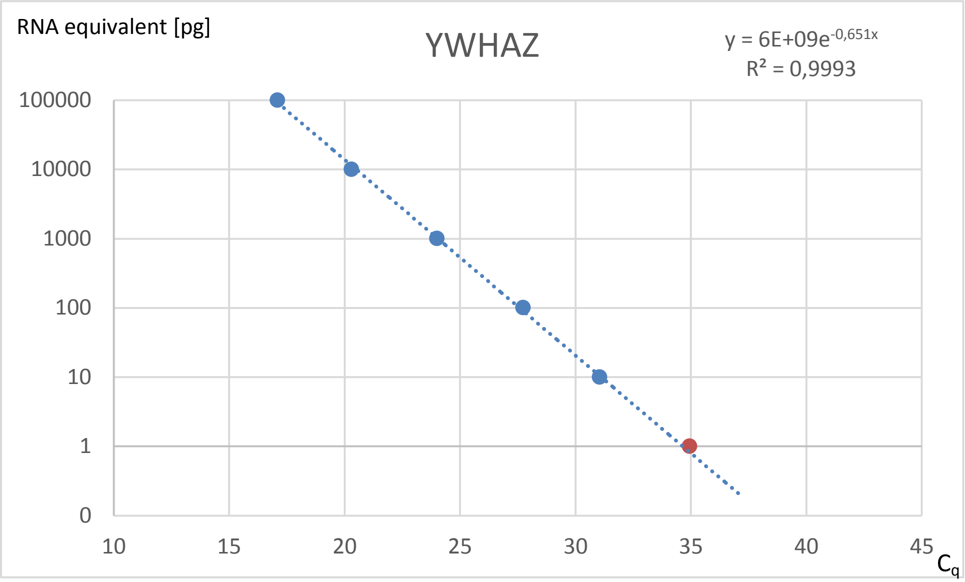

Slope [95% CI]	% Efficiency [95% CI]	LDR (dilution range)	LOD (dilution)
-3.488 [-3.915/-3.062]	93.5 [80.1/112.1]	$1:10 – 1:10^5$	$\leq 1:10^6$

SD = standard deviation; NTC = no-template control; LDR = linear dynamic range; LOD = limit of detection; R^2 = coefficient of determination; CI = confidence interval

<u>POLR2A</u> (factor-specific primer efficiency)

Gene	RNA equivalent [pg]	C_q Triplet	C_q 95%CI	C_q Mean	C_q SD	cDNA dilution
POLR2A	100000	20.01	19.93 /20.13	20.033	0.040	1:10
POLR2A	100000	20.08				
POLR2A	100000	20.01				
POLR2A	10000	23.11	23.01 /23.37	23.193	0.072	$1:10^2$
POLR2A	10000	23.23				
POLR2A	10000	23.24				
POLR2A	1000	26.72	26.31 /27.31	26.810	0.201	$1:10^3$
POLR2A	1000	27.04				
POLR2A	1000	26.67				
POLR2A	100	30.51	29.72 /31.47	30.593	0.352	$1:10^4$
POLR2A	100	30.29				
POLR2A	100	30.98				
POLR2A	10	36.85	30.14 /39.74	33.985	1.407	$1:10^5$
POLR2A	10	32.99				
POLR2A	10	34.98				
POLR2A	1	37.83	34.50 /39.49	36.993	1.004	$1:10^6$
POLR2A	1	35.88				
POLR2A	1	37.27				
POLR2A	NTC	37.11				

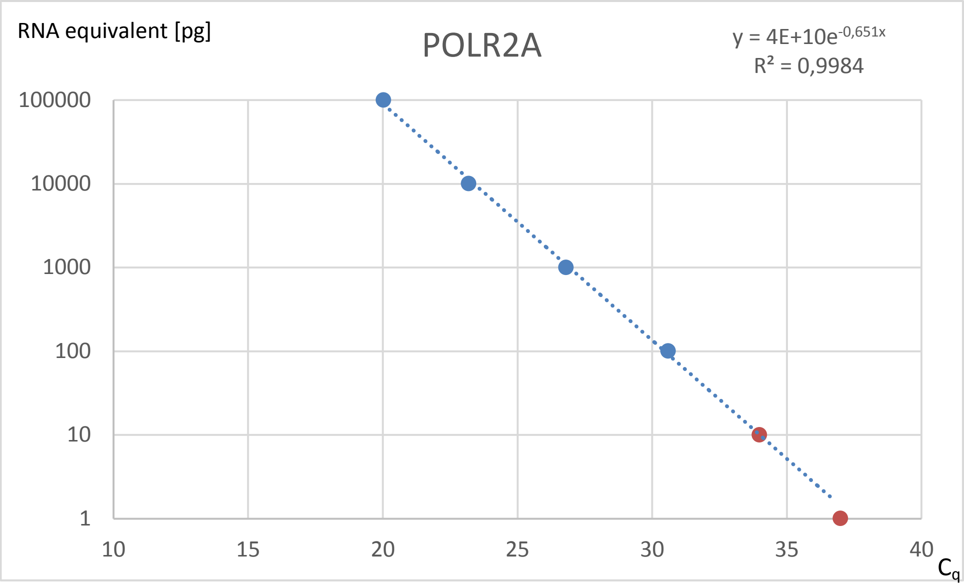

Slope [95% CI]	% Efficiency [95% CI]	LDR (dilution range)	LOD (dilution)
-3.520 [-4.321/-2.719]	92.3 [70.4/133.2]	$1:10 – 1:10^4$	$\leq 1:10^6$

SD = standard deviation; NTC = no-template control; LDR = linear dynamic range; LOD = limit of detection; R^2 = coefficient of determination; CI = confidence interval

TBP (factor-specific primer efficiency)

Gene	RNA equivalent [pg]	C_q Triplet	C_q 95%CI	C_q Mean	C_q SD	cDNA dilution
TBP	100000	26.53	26.46 /26.64	26.550	0.035	1:10
TBP	100000	26.53				
TBP	100000	26.59				
TBP	10000	29.80	29.72 /29.83	29.773	0.023	$1{:}10^2$
TBP	10000	29.76				
TBP	10000	29.76				
TBP	1000	33.35	32.96 /34.30	33.627	0.270	$1{:}10^3$
TBP	1000	33.89				
TBP	1000	33.64				
TBP	100	35.19	34.28 /37.07	35.677	0.561	$1{:}10^4$
TBP	100	36.29				
TBP	100	35.55				
TBP	10	-	-	41.110	-	$1{:}10^5$
TBP	10	41.11				
TBP	10	-				
TBP	1	-	-	39.080	-	$1{:}10^6$
TBP	1	39.08				
TBP	1	-				
TBP	NTC	-				

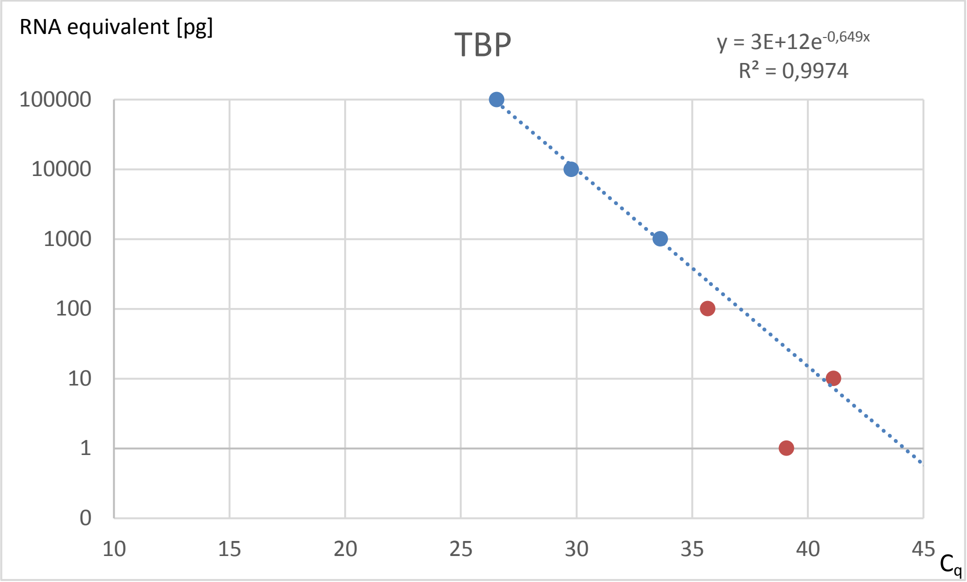

Slope [95% CI]	% Efficiency [95% CI]	LDR (dilution range)	LOD (dilution)
-3.538 [-7.540/0.465]	91.7 -	$1{:}10 – 1{:}10^3$	$1{:}10^4$

SD = standard deviation; NTC = no-template control; LDR = linear dynamic range; LOD = limit of detection; R^2 = coefficient of determination; CI = confidence interval

<u>RPL22</u> (factor-specific primer efficiency)

Gene	RNA equivalent [pg]	C_q Triplet	C_q 95%CI	C_q Mean	C_q SD	cDNA dilution
RPL22	100000	18.09	17.88 /18.19	18.037	0.061	1:10
RPL22	100000	17.97				
RPL22	100000	18.05				
RPL22	10000	20.86	20.77 /20.91	20.840	0.026	$1:10^2$
RPL22	10000	20.81				
RPL22	10000	20.85				
RPL22	1000	24.37	24.33 /24.40	24.367	0.015	$1:10^3$
RPL22	1000	24.38				
RPL22	1000	24.35				
RPL22	100	28.18	28.04 /28.45	28.247	0.083	$1:10^4$
RPL22	100	28.22				
RPL22	100	28.34				
RPL22	10	32.68	31.59 /33.24	32.413	0.333	$1:10^5$
RPL22	10	32.04				
RPL22	10	32.52				
RPL22	1	34.16	-	34.160	0.000	$1:10^6$
RPL22	1	34.16				
RPL22	1	34.16				
RPL22	NTC	-				

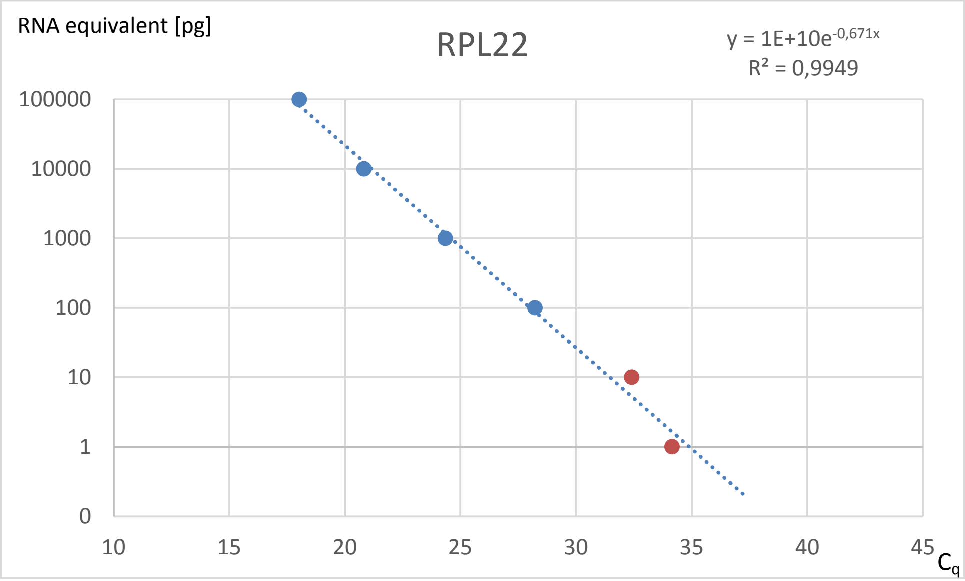

Slope [95% CI]	% Efficiency [95% CI]	LDR (dilution range)	LOD (dilution)
-3.403 [-4.767/-2.039]	96.7 [62.1/209.3]	$1:10 – 1:10^4$	$\leq 1:10^6$

SD = standard deviation; NTC = no-template control; LDR = linear dynamic range; LOD = limit of detection; R^2 = coefficient of determination; CI = confidence interval

<u>EEF1A1</u> (factor-specific primer efficiency)

Gene	RNA equivalent [pg]	C_q Triplet	C_q 95%CI	C_q Mean	C_q SD	cDNA dilution
EEF1A1	100000	16.72	16.25 /17.20	16.727	0.190	1:10
EEF1A1	100000	16.92				
EEF1A1	100000	16.54				
EEF1A1	10000	19.16	18.76 /19.61	19.187	0.172	$1:10^2$
EEF1A1	10000	19.37				
EEF1A1	10000	19.03				
EEF1A1	1000	22.54	22.21 /23.44	22.820	0.248	$1:10^3$
EEF1A1	1000	23.01				
EEF1A1	1000	22.91				
EEF1A1	100	25.53	24.81 /27.44	26.127	0.529	$1:10^4$
EEF1A1	100	26.31				
EEF1A1	100	26.54				
EEF1A1	10	30.23	29.38 /30.60	29.987	0.245	$1:10^5$
EEF1A1	10	29.74				
EEF1A1	10	29.99				
EEF1A1	1	33.03	31.70 /33.71	32.703	0.405	$1:10^6$
EEF1A1	1	32.83				
EEF1A1	1	32.25				
EEF1A1	NTC	-				

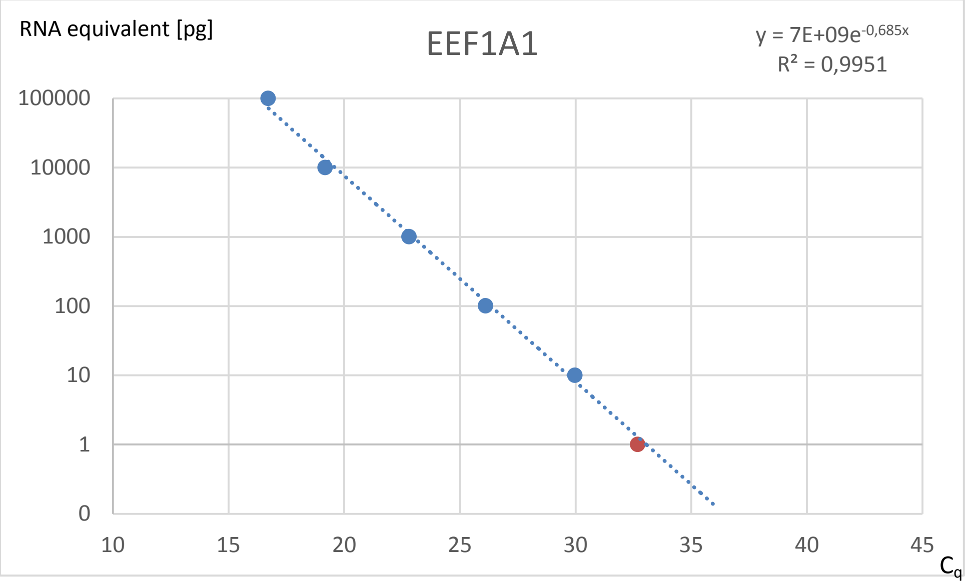

**Slope** [95% CI]	**% Efficiency** [95% CI]	**LDR** (dilution range)	**LOD** (dilution)
-3.315 [-4.291/-2.339]	100.3 [71.0/167.6]	$1:10 – 1:10^5$	$\leq 1:10^6$

SD = standard deviation; NTC = no-template control; LDR = linear dynamic range; LOD = limit of detection; R^2 = coefficient of determination; CI = confidence interval

<u>RPLP0</u> (factor-specific primer efficiency)

Gene	RNA equivalent [pg]	C_q Triplet	C_q 95%CI	C_q Mean	C_q SD	cDNA dilution
RPLP0	100000	15.15	14.94 /15.70	15.317	0.153	1:10
RPLP0	100000	15.35				
RPLP0	100000	15.45				
RPLP0	10000	18.52	18.26 /18.76	18.507	0.101	$1:10^2$
RPLP0	10000	18.40				
RPLP0	10000	18.60				
RPLP0	1000	22.30	22.18 /22.41	22.297	0.045	$1:10^3$
RPLP0	1000	22.34				
RPLP0	1000	22.25				
RPLP0	100	25.61	25.13 /26.93	26.027	0.363	$1:10^4$
RPLP0	100	26.20				
RPLP0	100	26.27				
RPLP0	10	29.30	28.94 /29.77	29.353	0.167	$1:10^5$
RPLP0	10	29.22				
RPLP0	10	29.54				
RPLP0	1	32.49	31.85 /32.73	32.290	0.176	$1:10^6$
RPLP0	1	32.16				
RPLP0	1	32.22				
RPLP0	NTC	39.62				

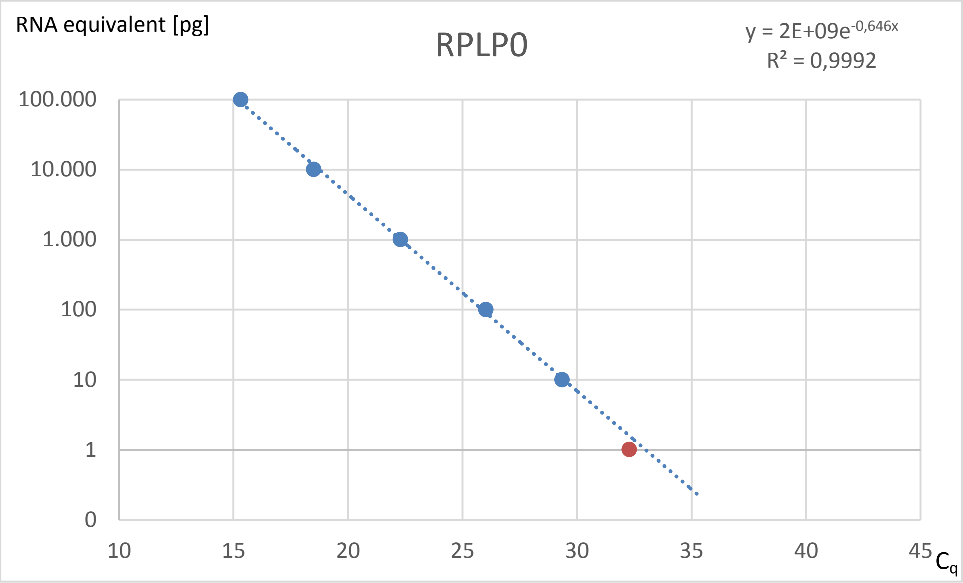

Slope [95% CI]	% Efficiency [95% CI]	LDR (dilution range)	LOD (dilution)
-3.509 [-3.980/-3.038]	92.7 [78.3/113.4]	$1:10 – 1:10^5$	$\leq 1:10^6$

SD = standard deviation; NTC = no-template control; LDR = linear dynamic range; LOD = limit of detection; R^2 = coefficient of determination; CI = confidence interval

RNA18S5 (factor-specific primer efficiency)

Gene	RNA equivalent [pg]	C_q Triplet	C_q 95%CI	C_q Mean	C_q SD	cDNA dilution
RNA18S	100000	4.65	4.57 /4.71	4.640	0.026	1:10
RNA18S	100000	4.66				
RNA18S	100000	4.61				
RNA18S	10000	7.06	7.00 /7.13	7.063	0.025	$1:10^2$
RNA18S	10000	7.09				
RNA18S	10000	7.04				
RNA18S	1000	10.68	10.54 /10.95	10.747	0.083	$1:10^3$
RNA18S	1000	10.84				
RNA18S	1000	10.72				
RNA18S	100	14.23	13.98 /14.88	14.433	0.182	$1:10^4$
RNA18S	100	14.49				
RNA18S	100	14.58				
RNA18S	10	17.66	17.50 /18.05	17.777	0.111	$1:10^5$
RNA18S	10	17.88				
RNA18S	10	17.79				
RNA18S	1	21.00	20.73 /21.74	21.233	0.202	$1:10^6$
RNA18S	1	21.35				
RNA18S	1	21.35				
RNA18S	NTC	35.26				

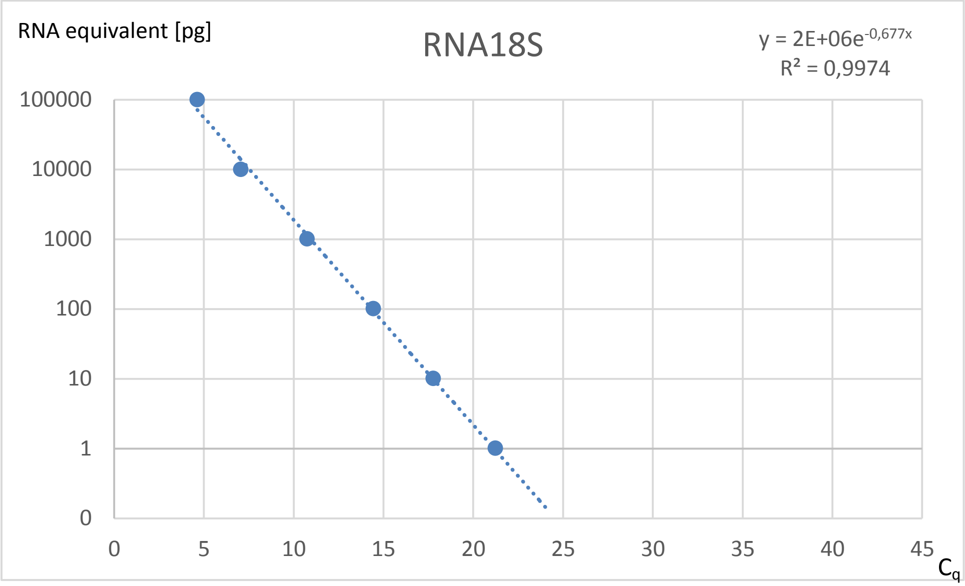

Slope [95% CI]	% Efficiency [95% CI]	LDR (dilution range)	LOD (dilution)
-3.319 [-3.967/-2.670]	100.1 [78.7/136.9]	$1:10 – 1:10^6$	$\leq 1:10^6$

SD = standard deviation; NTC = no-template control; LDR = linear dynamic range; LOD = limit of detection; R^2 = coefficient of determination; CI = confidence interval

Supplementary Data 5. Evaluation of a commercially available primer pair for TUBB (Qiagen, PPH17836A). Primer specifity was evaluated and confirmed by melting curve analysis (**a,b**) and agarose gel electrophoresis (**c**). **d** To calculate primer efficiency E_P, which was within the pre-specified acceptable range, a serial log_{10} dilution of cDNA stock solution (1,000,000 pg RNA equivalent) was performed in triplets. From the resulting C_q values a standard curve was created by linear regression. The 1:10 dilution used for qPCR for all genes/primers, however, was beyond the linear dynamic range LDR. **e** Amplification efficiency E_A was calculated with LinRegPCR and within the pre-specified acceptable range. **f** Raw qPCR C_q values for TUBB (triplet means). **g** Reference gene stability rankings including TUBB as 10th candidate reference gene indicate low intergroup expression stability in hPDL experiments on orthodontic tooth movement and periodontitis.

(a) Melting curve analysis and Amplification plot (main qPCR)

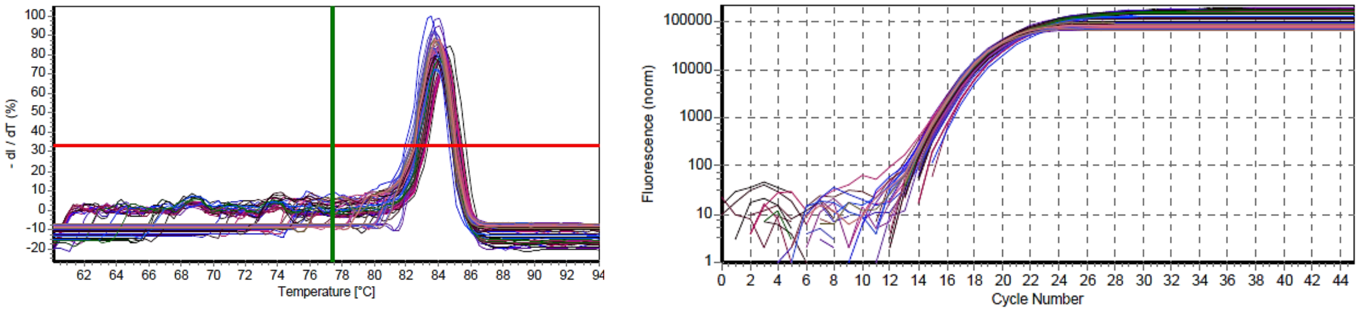

(b) Melting curve analysis and Amplification plot (efficiency qPCR – standard curve)

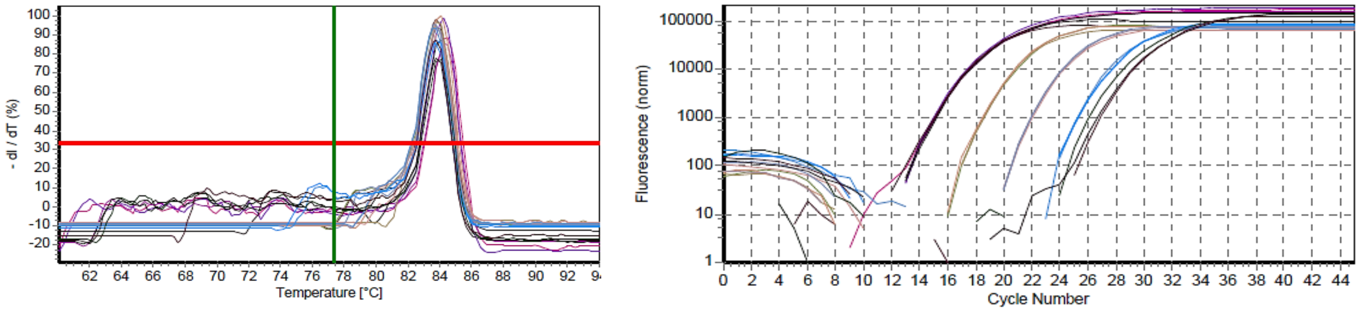

(c) Agarose gel electrophoresis (main qPCR)

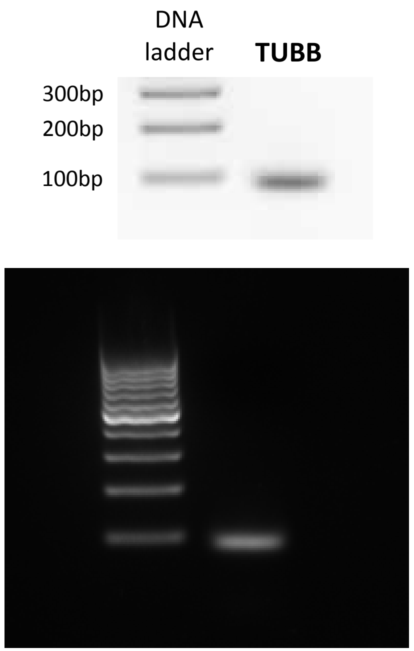

(d) TUBB Primer efficiency (factor-specific)

Gene	RNA equivalent [pg]	C_q Triplet	C_q 95%CI	C_q Mean	C_q SD	cDNA dilution
TUBB	100000	19.65	19.38 /19.79	19.583	0.083	1:10
TUBB	100000	19.49				
TUBB	100000	19.61				
TUBB	10000	19.45	17.21 /20.34	18.777	0.631	1:10^2
TUBB	10000	18.68				
TUBB	10000	18.20				
TUBB	1000	21.67	21.07 /21.93	21.500	0.175	1:10^3
TUBB	1000	21.32				
TUBB	1000	21.51				
TUBB	100	24.87	24.81 /25.03	24.920	0.046	1:10^4
TUBB	100	24.93				
TUBB	100	24.96				
TUBB	10	28.16	27.80 /29.14	28.467	0.270	1:10^5
TUBB	10	28.57				
TUBB	10	28.67				
TUBB	1	30.40	29.87 /31.75	30.807	0.379	1:10^6
TUBB	1	31.15				
TUBB	1	30.87				
TUBB	NTC	42.23				
TUBB	-RT	32.31				

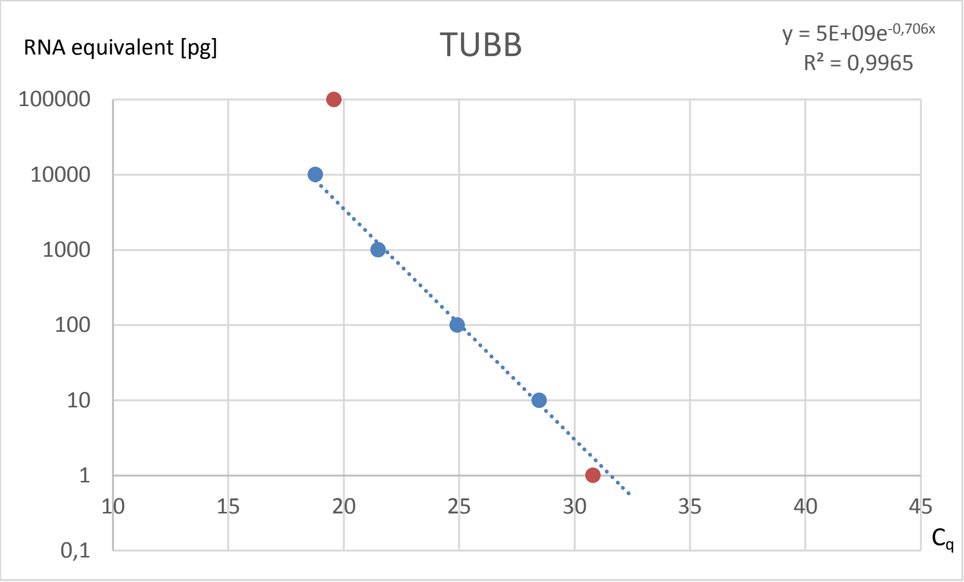

Slope [95% CI]	% Efficiency [95% CI]	LDR (dilution range)	LOD (dilution)
-3.230 [-4.33/-2.13]	104.0 [52.2/165.0]	1:10^2 – 1:10^5	≤1:10^6

SD = standard deviation; NTC = no-template control; -RT = control without reverse transcriptase; LDR = linear dynamic range; LOD = limit of detection; R^2 = coefficient of determination; CI = confidence interval

(e) Primer efficiency (factor-specific) and coefficients of determination derived from a standard curve for TUBB (6x log_{10} dilution of cDNA stock solution, random untreated sample) as well as technical repeatability (intraassay reliability, n = 18) and amplification efficiency (sample-specific), calculated using LinRegPCR software (http://LinRegPCR.HFRC.nl; n = 18 in triplets).

Gene symbol	Slope	Primer efficiency E_P [%] ($2^{E_P/100\%}$)	Coefficient of determination R^2	Intraassay reliability SD of mean of C_q* (mean, min./max.)	Amplification Efficiency E_A [%] ($2^{E_A/100\%}$)
**TUBB**	-3.230	104.0 (2.056)	0.9965	0.26<br>0.05 / 0.63	91.3 (1.883)

* of three technical replicates (triplet) among all biological replicates (n = 18). CI = confidence interval

(f) Raw C_q values (triplet means) of TUBB RT-qPCR for the three experimental groups.

**RAW C_q values**<br>**Sample**	 **Group**	Gene<br>**TUBB**
Control K7	1	20.10
Control K8	1	20.71
Control K9	1	19.58
Control K10	1	19.53
Control K11	1	19.54
Control K12	1	19.30
Compression D7	2	18.84
Compression D8	2	19.06
Compression D9	2	19.88
Compression D10	2	19.10
Compression D11	2	18.55
Compression D12	2	19.50
Agac7	3	19.30
Agac8	3	18.49
Agac9	3	18.29
Agac10	3	18.53
Agac11	3	19.19
Agac12	3	18.38
**C_q SD Control**	1	0.52
**C_q SD Compression**	2	0.47
**C_q SD Agac**	3	0.43

C_q = quantification cycle; SD = standard deviation of group mean.
Agac = Aggregatibacter actinomycetemcomitans (periodontitis)

(g) Reference gene stability ranking including TUBB for hPDL experiments on orthodontic tooth movement (compressive orthodontic force vs. untreated control), experiments on periodontitis (Agac, toxins/bacterial lysate vs. untreated control) and pooled/overall experimental conditions as calculated by the algorithms geNorm, NormFinder, comparative ΔC_q and BestKeeper. A higher rank denotes lower expression stability.

Rank	Total (of 4 methods)		geNorm		NormFinder			comparative deltaC$_q$		BestKeeper			
	Ranking order	Rank sum	Ranking order	Stability value (M)	Ranking order	Stability value (ρ_{ig}/σ_i)	Standard error	Ranking order	Stability value (mean SD of mean ΔC_q)	Ranking order	Stability value (r)	SD (+/- C_q)	CV (% C_q)
**hPDL untreated + compressive orthodontic force** (experiments on orthodontic tooth movement, n = 12)													
1.)	**RPL22**	**6**	**RPL22**	**0.263**	**RPL22**	**0.043**	**0.033**	**RPL22**	**0.271**	**RNA18S5**	**0.910**	**0.259**	**3.110**
2.)	**PPIB**	**13**	**PPIB**	**0.286**	**EEF1A1**	**0.092**	**0.031**	**PPIB**	**0.296**	**YWHAZ**	**0.905**	**0.373**	**1.728**
3.)	***RPLP0***	***17***	***RPLP0***	***0.296***	***RPLP0***	***0.097***	***0.031***	***RPLP0***	***0.307***	***RPL22***	***0.856***	***0.121***	***0.665***
4.)	TBP	18	TBP	0.299	PPIB	0.099	0.031	EEF1A1	0.311	***TBP***	***0.657***	***0.202***	***0.860***
5.)	EEF1A1	18	EEF1A1	0.302	TBP	0.121	0.034	TBP	0.314	***PPIB***	***0.627***	***0.187***	***1.128***
6.)	RNA18S5	19	RNA18S5	0.347	RNA18S5	0.152	0.039	RNA18S5	0.350	POLR2A	0.533	0.357	1.681
7.)	YWHAZ	29	GAPDH	0.350	GAPDH	0.166	0.041	GAPDH	0.365	EEF1A1	0.505	0.114	0.814
8.)	POLR2A	30	POLR2A	0.399	POLR2A	0.230	0.053	POLR2A	0.423	RPLP0	0.364	0.098	0.601
9.)	GAPDH	31	YWHAZ	0.424	YWHAZ	0.234	0.054	YWHAZ	0.449	TUBB	0.187	0.420	2.157
10.)	TUBB	39	TUBB	0.678	TUBB	0.449	0.097	TUBB	0.664	GAPDH	-0.154	0.117	0.776
**hPDL untreated + Agac toxins/bacterial lysate** (experiments on periodontitis, n = 12)													
1.)	**PPIB**	**8**	**PPIB**	**0.250**	**PPIB**	**0.066**	**0.031**	**PPIB**	**0.255**	**RNA18S5**	**0.815**	**0.269**	**3.261**
2.)	**TBP**	**10**	**TBP**	**0.259**	**TBP**	**0.077**	**0.031**	**TBP**	**0.265**	**POLR2A**	**0.599**	**0.176**	**0.836**
3.)	POLR2A	18	EEF1A1	0.269	GAPDH	0.116	0.034	EEF1A1	0.270	YWHAZ	0.567	0.318	1.488
4.)	***RPL22***	***20***	***RPL22***	***0.284***	POLR2A	0.121	0.035	***RPL22***	***0.290***	TBP	0.514	0.121	0.518
5.)	EEF1A1	21	POLR2A	0.286	***RPL22***	***0.122***	***0.035***	GAPDH	0.295	PPIB	0.449	0.099	0.602
6.)	GAPDH	21	RPLP0	0.286	EEF1A1	0.128	0.036	RPLP0	0.296	GAPDH	0.420	0.159	1.044
7.)	RNA18S5	25	GAPDH	0.287	RPLP0	0.140	0.038	ROLR2A	0.297	***RPL22***	***0.343***	***0.164***	***0.902***
8.)	RPLP0	29	RNA18S5	0.380	RNA18S5	0.166	0.042	RNA18S5	0.381	TUBB	0.324	0.558	2.897
9.)	YWHAZ	30	YWHAZ	0.420	YWHAZ	0.203	0.049	YWHAZ	0.439	EEF1A1	0.313	0.167	1.170
10.)	TUBB	38	TUBB	0.815	TUBB	0.554	0.119	TUBB	0.792	RPLP0	0.291	0.172	1.049
**hPDL pooled/overall** (experiments on orthodontic tooth movement and periodontitis n = 18)													
1.)	**PPIB**	**7**	**PPIB**	**0.296**	**PPIB**	**0.076**	**0.026**	**PPIB**	**0.306**	**RNA18S5**	**0.859**	**0.266**	**3.199**
2.)	**TBP**	**12**	**TBP**	**0.304**	**RPL22**	**0.093**	**0.026**	**RPL22**	**0.313**	**YWHAZ**	**0.759**	**0.381**	**1.777**
3.)	***RPL22***	***12***	***RPL22***	***0.304***	***TBP***	***0.096***	***0.026***	***TBP***	***0.316***	***TBP***	***0.625***	***0.173***	***0.735***
4.)	RPLP0	19	RPLP0	0.326	RPLP0	0.135	0.030	RPLP0	0.338	PPIB	0.587	0.158	0.955
5.)	RNA18S5	19	EEF1A1	0.357	RNA18S5	0.159	0.033	EEF1A1	0.362	POLR2A	0.525	0.280	1.320
6.)	EEF1A1	24	POLR2A	0.373	EEF1A1	0.171	0.035	RNA18S5	0.383	RPL22	0.485	0.141	0.776
7.)	POLR2A	25	RNA18S5	0.379	POLR2A	0.184	0.037	POLR2A	0.391	RPLP0	0.262	0.149	0.913
8.)	YWHAZ	29	GAPDH	0.384	GAPDH	0.195	0.038	GAPDH	0.400	EEF1A1	0.261	0.181	1.277
9.)	GAPDH	34	YWHAZ	0.465	YWHAZ	0.253	0.047	YWHAZ	0.491	TUBB	0.236	0.501	2.605
10.)	TUBB	39	TUBB	0.744	TUBB	0.491	0.085	TUBB	0.726	GAPDH	0.057	0.189	1.245

C_q = quantification cycle; SD = standard deviation; CV = coefficient of variation; r = Pearson's correlation coefficient
